# Molluscan Compounds Provide Drug Leads for the Treatment and Prevention of Respiratory Disease

**DOI:** 10.3390/md18110570

**Published:** 2020-11-19

**Authors:** Kate Summer, Jessica Browne, Lei Liu, Kirsten Benkendorff

**Affiliations:** 1 Marine Ecology Research Centre, School of Environment, Science and Engineering, Southern Cross University, GPO Box 157, Lismore, NSW 2480, Australia; kate.summer@scu.edu.au; 2 School of Health and Human Sciences, Southern Cross University, Terminal Drive, Bilinga, QLD 4225, Australia; jessica.browne@scu.edu.au; 3 Southern Cross Plant Science, Southern Cross University, GPO Box 157, Lismore, NSW 2480, Australia; ben.liu@scu.edu.au; 4 National Marine Science Centre, Southern Cross University, 2 Bay Drive, Coffs Harbour, NSW 2450, Australia

**Keywords:** marine natural products, ethnomedicine, coronavirus, hemocyanin, Mollusca, pulmonary, lung

## Abstract

Respiratory diseases place an immense burden on global health and there is a compelling need for the discovery of new compounds for therapeutic development. Here, we identify research priorities by critically reviewing pre-clinical and clinical studies using extracts and compounds derived from molluscs, as well as traditional molluscan medicines, used in the treatment of respiratory diseases. We reviewed 97 biomedical articles demonstrating the anti-inflammatory, antimicrobial, anticancer, and immunomodulatory properties of >320 molluscan extracts/compounds with direct relevance to respiratory disease, in addition to others with promising bioactivities yet to be tested in the respiratory context. Of pertinent interest are compounds demonstrating biofilm inhibition/disruption and antiviral activity, as well as synergism with approved antimicrobial and chemotherapeutic agents. At least 100 traditional medicines, incorporating over 300 different mollusc species, have been used to treat respiratory-related illness in cultures worldwide for thousands of years. These medicines provide useful clues for the discovery of bioactive components that likely underpin their continued use. There is particular incentive for investigations into anti-inflammatory compounds, given the extensive application of molluscan traditional medicines for symptoms of inflammation, and shells, which are the principal molluscan product used in these preparations. Overall, there is a need to target research toward specific respiratory disease-related hypotheses, purify bioactive compounds and elucidate their chemical structures, and develop an evidence base for the integration of quality-controlled traditional medicines.

## 1. Introduction

### 1.1. Respiratory Disease Pathology and Epidemiology

The role of the respiratory system is to allow continuous O_2_ and CO_2_ exchange with the environment [1]. In doing so, it incidentally permits exposure to airborne particles, chemicals, and infectious organisms with each breath [1,2]. As such, the nature and types of respiratory disease are many and physiological defense mechanisms must be well regulated. Also known as lung or pulmonary diseases, respiratory diseases are conditions affecting the lungs and other tissues of the respiratory system impairing normal gas exchange [3].

Respiratory diseases are among the leading causes of death and disability worldwide [2]. Asthma, chronic obstructive pulmonary disease (COPD), lung cancer, and communicable infections impose a particularly immense burden on global health with staggering rates of morbidity and mortality [4] (Table 1). COPD affects over 200 million people at any one time, while lung cancer is the most common type of malignancy and responsible for the highest number (19%) of cancer-related deaths [4] (Table 1). Around 340 million people are afflicted with asthma and 24 million disability-adjusted life years (DALYs) are lost to the condition each year [5] (Table 1). Acute bacterial and viral respiratory infections are considered the greatest single contributor to the overall burden of disease worldwide; they are the leading cause of death in developing countries and in children under five [4,6] (Table 1), and can also occur in epidemics and pandemics, as we were made poignantly aware by outbreaks of severe acute respiratory distress syndrome (SARS) in 2002 [7], Middle East respiratory syndrome (MERS) in 2012 [8], and the novel coronavirus in 2019 (COVID-19) [9] (Table 1).

Acute respiratory conditions, such as acute respiratory distress syndrome (ARDS) and pneumonia, are typically sudden and severe and may be caused by infection, trauma, or hypersensitivity [10,11] (Table 1). Conversely, chronic conditions, such as COPD, cystic fibrosis, and cancer, are ongoing, progressive, and generally attributed to lifestyle (e.g., air pollution and smoking) or genetic factors [4,12,13] (Table 1). However, the distinction between acute and chronic is not always discrete. Chronic conditions may have acute episodes (e.g., asthma attacks) or be exacerbated by acute conditions (e.g., COPD with infection [14]), while acute conditions can progress into more chronic ones if persistent, recurrent, or severe (e.g., unresolving pneumonia [15]; ARDS causing pulmonary fibrosis [11]) (Table 1). Despite their varied aetiologies and symptoms, all respiratory diseases evoke a strong immune response characterized by inflammation.

The inflammatory pathway is the universal physiological basis underpinning immune responses to tissue damage, infection, and other insults [16] and is therefore a feature of every respiratory disease. A common spectrum of genes and endogenous mediators are involved, although the precise physiological and biochemical components that are induced vary in different conditions [17]. A typical response consists of stimulants (e.g., pathogenic microbial patterns, allergens, free radicals released from damaged cells, tumor antigens), the specialised cells that identify them (e.g., granulocytes, mast cells, macrophages, dendritic cells, memory lymphocytes), chemical mediators (e.g., complement, cytokines, chemokines, eicosanoids, amines, antibodies), and functional changes in target tissues and cells that enable elimination of the stimulant (e.g., vasodilation, neutrophil infiltration, phagocytosis, mucus secretion) [16]. This is a natural, and for the most part beneficial, response but tight regulation is critical [10]. A hyper-inflammatory response can be immediately life-threatening (e.g., [18,19]) and if the inducer is not quickly overcome or resolution is incomplete, cellular and tissue damage may ensue, leading to a chronic disease state (e.g., [20]). Respiratory disease pathogenesis and immune mechanisms are described elsewhere in detail [10,21,22,23,24,25] to assist in structure-target identification.

**Table 1:** Epidemiological summary of respiratory diseases imposing a major burden on health worldwide.

Respiratory Disease *	Disease Classification	Causative/Risk Factors	Predominant Symptoms	Estimated Worldwide Morbidity (Annual) (P: Prevalence, I: Incidence)	Estimated Worldwide Mortality (Annual)	Conventional Treatments	Trends	Ref
Infectious diseases (e.g., TB; NTM; influenza; pneumonia †; corona viruses)	- Communicable ‡ - Acute - May become chronic if unresolving or recurrent	- Opportunistic bacterial; viral; fungal or parasitic invasion - Risk factors: low immunization rates; overpopulation; conditions of compromised immunity	Inflammation; cough; increased mucus; fever; dyspnea; tachypnea; malaise; muscle and join pain; sore throat; secondary infections	- TB: 10 million (P) - NTM: 40 per 100,000 population (P) - Influenza: 5–15% of population; 3–5 million severe cases (I) - RSV: 34 million child episodes (I) - COVID-19: >56 million ^§^ (P)	- >4 million total ^¶^ - TB: 1.5 million - Pneumonia: >1.3 million children <5 y (15% of all deaths) - Seasonal influenza: 290,000–650,000 - COVID-19: 1,500,000 ^§^	- Antibiotics (bacterial) - Neuraminidase inhibitors (viral) - Symptomatic treatment - Immunization	- Consistently within top 3 causes of death - TB incidence declining by 2%/y, NTM increasing 40%/y - Increasing epidemics and drug resistant strains - Highest impact in developing countries	[2,26,27,28]
Chronic obstructive pulmonary disease (COPD)	- Non-communicable ‡ - Chronic with acute episodes - Progressive - Irreversible	- Tobacco smoke and other inhaled environmental pollutants - Frequent/chronic lower respiratory infections, asthma and abnormal lung development - Genetic factors	Chronic parenchymal and airway inflammation; persistent airflow restriction; dyspnea; wheeze; cough; decreased airway elasticity; airway remodeling; mucociliary dysfunction; co-morbidities	- >250 million (P) - Usually becomes apparent in people >40 yes of age	- 3.2 million - Third leading cause of death	- Cessation of smoking - Symptomatic treatment - Inhaled corticosteroids; long-acting β agonists; leukotriene modifiers - Immunization against infectious diseases	- Increasing prevalence and mortality rates	[2,27,29]
Asthma	- Non-communicable - Chronic with acute episodes - Reversible	- Genetic factors - Environmental triggers - Airway hyperresponsiveness	Airflow restriction; wheeze; dyspnea; cough; airway remodeling	- >339 million (P) - 24.8 million DALYs lost	- Relatively low mortality rate - 420,000 (2016)	- Medications for: rescue (e.g., fast-acting β agonists), maintenance (e.g., inhaled corticosteroids; long-acting β agonists; leukotriene modifiers) and allergies - Avoidance of triggers	- Increasing incidence - Highest mortality (80%) in developing countries	[2,5,27,30,31]
Lung cancer	- Non-communicable - Chronic - Progressive	- Tobacco smoke and other inhaled environmental pollutants - Physical carcinogens (e.g., ionizing radiation) - Other chronic respiratory diseases - Genetic factors	Dyspnea; hoarseness; hemoptysis; pain; loss of appetite; weight loss; fatigue; persistent cough	- 2.09 million (P) - 1.8 million (I)	- 1.76 million	- Surgery - Radiation and chemotherapy - Palliative care and psycho-social support	- 15% of diagnosed cancers - Most fatal cancer - 19% of deaths	[2,6,27]
Acute respiratory distress syndrome (ARDS)/acute lung injury (ALI)	- Non-communicable - Acute	- Trauma - Pulmonary infection - Non-pulmonary sepsis - Certain medical procedures	Severe dyspnea and tachypnea; pulmonary hemorrhage; edema; hypertension; hypoxemia; tissue damage; fibrosing alveolitis	- ARDS: 58.7–75 per 100,000 people (I) - ALI: 78.9 per 100,000 people (I)	- In-hospital mortality 38% for ALI; up to 46.1% for ARDS	- Corticosteroids and other anti-inflammatories; vasodilators - Mechanical ventilation - Hemodynamic management - Surfactant therapy	- 10% of all patients in intensive care treated for ARDS - Survival rates improving	[11,19]
Cystic fibrosis	- Non-communicable - Chronic with acute episodes - Progressive	- Autosomal recessive genetic factors (CFTR mutation) - Exacerbated by environmental triggers and infection ^#^	Bronchiecstasis; persistent airway infection and inflammation; excessive, thick mucus and poor clearance; pneumothorax; hemoptysis; tissue damage; gastrointestinal, metabolic and reproductive manifestations	- 90,000 (P) (likely underestimated) - 1000 (I)	- More than half of patients die before the age of 18	- Antibiotics - Immunisations - Nebulised hypertonic saline; dornase alfa; mannitol - CFTR modulators (e.g., Ivacaftor) - O_2_ therapy; pulmonary rehab. - Management of co-morbidities and nutrition - Lung transplant	- Survival rates improving	[32]

Abbreviations: DALYs: disability-adjusted life years- a metric that estimates the amount of active and productive life lost due to a condition; TB: tuberculosis; NTM: non-tuberculosis mycobacteria; RSV: respiratory syncytial virus; COVID-19: 2019 novel corona virus; CFTR: cystic fibrosis transmembrane conductance regulator; NO: nitric oxide. * Other important respiratory diseases not described in this table include sleep disordered breathing, pulmonary hypertension, and pulmonary embolism. † Typically caused by *Streptococcus pneumoniae*, *Haemophilus influenzae* type b, respiratory syncytial virus; *Pneumocystis jiroveci* (in HIV infected). ‡ Communicable refers to diseases caused by infectious agents that can be transmitted from one person (or animal) to another via. direct or indirect contact. Non-communicable diseases are not transmitted from one person (or animal) to another. ^§^ From December 2019 to time of publication. ^¶^ The annual mortality rate from respiratory infection is difficult to quantify, particularly in developing countries, and estimates vary considerably e.g., van der Poll & Opal (2009) estimate mortality rate of 4 million caused by pneumococcal pneumonia alone. ^#^ Particularly of *Pseudemonas aeruginosa, Burkholdaria cepacia, Staphylococcus aureus,* often multi-drug resistant.

### 1.2. Conventional Treatments and the Need for Alternatives

The discovery of antibiotics, vaccines, chemotherapeutic agents, and non-steroidal anti-inflammatory drugs (NSAIDs) revolutionised the treatment and prevention of respiratory diseases [33] and remarkably improved life expectancy from 30.9 years in 1900 to 46.7 years in 1940 and 61.1 years in 1980 [34]. These drugs are now the most consumed worldwide [35,36]. Nonetheless, they have not come without pitfalls, which now pose immense challenges to modern medicine, the most serious being the development of undesirable side-effects and drug resistance (e.g., [37,38,39,40]). These issues unfortunately decrease the arsenal of available therapeutic agents against an ever-increasing range of diseases, at a high social and economic cost [41]. Further, there are dire ecotoxicological consequences associated with the widespread use and distribution of synthetic pharmaceutical substances throughout the environment [42,43]. 

In light of the ominous emergence of new and resistant pathogens, the rising incidence of respiratory diseases, and impacts of conventional drugs, there is an urgent need for the discovery of novel, ideally safer, compounds for therapeutic development. This need is widely acknowledged among the scientific community, health professionals, and policy makers alike (e.g., [4,40,44,45]). Natural products play a pivotal role in drug development and research programs considering their incomparable chemical diversity and novel mechanisms of action [46]. They also generally exhibit lower systemic and environmental toxicity than their synthetic pharmaceutical counterparts [47,48,49]. Marine organisms represent an especially rich source of structurally diverse natural products by virtue of their vast phylogenetic diversity and environmental conditions under which they have evolved [50]. These compounds are evidently valuable as drugs leads: to date, over 30 marine compounds have entered clinical trials for drug development, of which 12 have been approved for use by the Food and Drug Administration (FDA) and international counterparts [51,52]. 

### 1.3. Molluscs: A Wealth of Potential Therapeutic Compounds

Mollusca represents the second-most species-rich phylum with an estimated 150,000–200,000 extant species divided into eight classes [53,54]. Molluscs have diversified to occupy almost every ecosystem on earth, from arid deserts to alpine regions, yet the majority occur in the sea where they account for around a quarter of all known species [55]. Such vast biological diversity affords Mollusca a rich chemical diversity [49,53,56]. Irrespective of the presence of a shell, all molluscs are essentially soft-bodied and lack adaptive immune systems with antigen-specific cell-mediated mechanisms of protection [53]. Many are also slow-moving or sessile and thus seemingly predisposed to biofilm formation [53,57]. Molluscs could, therefore, be vulnerable to infection, yet reside in intimate coexistence with pathogenic microbes [53,57]. Seawater is known to contain up to 10^6^ bacteria and 10^9^ virus per mL [58,59], and soil up to 10^10^ bacteria and 10^9^ virus per gram [60]. The survival and success of molluscs in microbially rich environments is attributed to robust chemical defenses and innate humoral immune components, including hemocyanins (Hcs) and antimicrobial peptides (AMPs) [53,57,61]. Molluscs have also been shown to use chemical means of communication, predator-prey interaction and behavior [62,63,64,65], further demonstrating their diverse functional chemistry.

The chemical investigation of molluscs has led to the isolation of a wide variety of bioactive primary and secondary metabolites. These may be synthesized by the molluscs themselves, accumulated from dietary sources, or produced by symbionts [66]. Benkendorff [53,67] established that, as of 2014, more than 1145 compounds had been isolated from marine molluscs, including peptides, sterols, terpenes, polyproprionates, macrolides, fatty acid derivatives, nitrogenous compounds, and alkaloids. An additional 145 molluscan compounds have since been documented in annual Marine Natural Product Reports covering years 2014–2018 [68,69,70,71,72]. 

Bioassay-guided investigations and in vivo models have demonstrated the antimicrobial (e.g., [45,73,74]), anticancer [48,75,76], anti-inflammatory [49,77,78], antispasmodic [79,80], neuromuscular blocking [81], wound-healing [82], and immunogenic [83,84] properties of molluscan extracts and purified compounds. At least 19 compounds of molluscan origin are currently part of the global marine pharmaceutical clinical pipeline and four are now FDA approved [51,85], including the powerful analgesic Ziconotide derived from the venom of *Conus magus* [86], a carnivorous cone snail, and three dolastatin derivatives originally sourced from the tissue of the sea hare *Dolabella auricularia*: Brentuximab vedotin [87] and Polatuzumab vedotin [88] for the treatment of hematologic cancers (e.g., Hodgkins lymphoma) and Enfortumab vedotin for urotherial cancer [89]. Nonetheless, more than half of the known molluscan secondary metabolites have never been screened for potential bioactivity, let alone entered in vivo studies or clinical trials, and <1% of molluscan species have been investigated [67]. Indeed, the therapeutic potential of natural products derived from this phylum is enormous.

A surprisingly large number of traditional medicines incorporating molluscs have been used to treat respiratory-related illness in cultures worldwide from antiquity to present [90,91] (Table 2, Tables S1 and S2). In most cases, there is little data to support the application of traditional medicines [90,92]. However, they provide useful clues for the discovery of novel compounds that may underpin some claims [90] and adequate testing could see their integration into mainstream healthcare in a complementary capacity at least. To illustrate this point, the isolation of ephidrine from *Ephedra sinica* (Ma Huang), a plant used in Traditional Chinese Medicine (TCM) for over 2000 years to treat asthma, cough and respiratory congestion, led to the synthesis of salbutamol (Ventolin) in 1969, a fast acting β2-adrenergic receptor agonist now considered the first-line clinical intervention for acute asthma and COPD [93,94]. Moreover, molluscs are rich in essential nutrients and polyunsaturated fatty acids (PUFAs) [95] and high dietary intake of shellfish and PUFAs has been associated with lower risk of chronic respiratory conditions and improved health outcomes [96,97,98,99,100,101].

There is mounting evidence that molluscs comprise of pharmacologically active compounds that could be optimized for the treatment and prevention of respiratory disease. The aim of this review was to critically analyse in vitro and in vivo studies, human clinical trials, and approved therapies using molluscan extracts/compounds, as well as reports on the traditional medicinal uses of molluscs, used in the treatment of respiratory diseases or symptoms thereof. This pertinent review may help to identify research needs and priorities for the future development of therapeutic agents and approaches used to address respiratory diseases mediated by pathogenic infection, inflammation, malignancy, and hypersensitivity.

## 2. Literature Search Methods and Evaluation

We undertook an extensive scoping review of published biomedical and ethnomedical literature using scientific databases Scopus, Web of Science, and PubMed. Databases were searched by article title, abstract, and keywords without date limits (as of October 2020). Full search criteria are provided as Supplementary Information. All records were screened by title and abstract before full-text articles were assessed.

Over 900 biomedical articles were screened based on their relevance to respiratory disease and focus on bioactivity of the molluscan extract or compound/s. In vitro studies were accepted if the test suite included at least one respiratory pathogenic microorganism or cell line of respiratory origin. Other non-respiratory microorganisms, cell types, and models that may also have been tested are not described herein, unless results were useful for comparison to respiratory microorganisms, cell types, or models. In vivo animal models and human clinical trials were accepted if the disease was specific to the respiratory system (e.g., asthma, infection, COPD, lung cancer) and the bioactivity of the molluscan extract/compound was evident in the results. Search results returned copious studies using Hc as a vaccine adjuvant or conjugate which were only accepted if treatments (or controls) used Hc alone or the antigen both with and without Hc. In vivo immunological studies using Hc as a model antigen were separated from those using Hc as a treatment or treatment component and accepted if the investigation was respiratory related. Key review articles [49,53,56,66,90,102,103] were searched for relevant references therein, which were then screened by abstract. Many of the studies included in previous reviews provide further insight into the bioactivity and immunogenicity of molluscan extracts/compounds and their potential to treat respiratory diseases, however are not specific to these diseases and therefore not included herein. In total, 97 peer-reviewed biomedical articles are included in this review. Those with substantial in vivo and in vitro components were regarded as separate studies in tables and analyses (total: 54 in vitro studies, 25 in vivo studies, 11 human clinical trials and 16 in vivo human/animal studies in which Hc was used as a model antigen).

For literature regarding traditional medicinal applications, database searches returned 113 results, minus duplicates. Key review papers by Ahmad et al. [49] and Benkendorff et al. [90] provided important references to books and monographs not recorded in databases. The Chinese *Marine Materia Medica* [104] was translated by the third author of this review. All texts (published in English or otherwise translated) were screened for mollusc-derived remedies relating to the treatment of respiratory disease or symptoms, and appear directly as translated with contemporary revisions provided in footnotes. In total, 22 traditional medicine studies/texts are included in this review. The phrase “other traditional medicines” used throughout refers to traditional medicines other than TCMs. We use the term traditional or molluscan “medicine” to refer to any concoction traditionally used to treat human ailments that contain molluscan products functioning as potential active pharmaceutical ingredients (APIs).

Data were collated and descriptive statistics calculated in Excel. Summary tables are provided throughout the review and as Supplementary Files. Table 2 summarises the number of different remedies and species traditionally used to treat specific symptoms of respiratory disease; full details of traditional medicines, including species, preparations, and indications are provided in Supplementary Tables S1 and S2. Table 3 provides a taxonomic breakdown of the number of species in each molluscan class as well as their division by terrestrial, freshwater, and marine habitat, that are used in traditional medicines and compares these to biomedical studies, whereas Table 4 provides the number of studies and species tested for different bioactivities. In both cases, the species, extract or compound, experimental design, effective concentrations and outcomes are detailed in Table 5, Table 6, Table 7 and Table 8 to inform research direction and provide evidence that molluscan compounds could be the APIs in traditional medicines. Studies using molluscan Hcs as vaccine conjugates or model antigens are included in summary Table 2 and Table 3 and detailed in Supplementary Tables S3 and S4. Selected patents and purified compounds that have been tested with relevance to respiratory disease are provided in Supplementary Tables S5 and S6, respectively. Taxonomic nomenclature of the mollusc species used throughout this review is corrected according to the World Register of Marine Species [105]. Where corrected, reference to species names used in the cited publications is provided in footnotes.

## 3. Uses of Molluscs in Traditional Medicines for Respiratory Disease

### 3.1. Traditional Molluscan Respiratory Medicines

**Figure 1:**
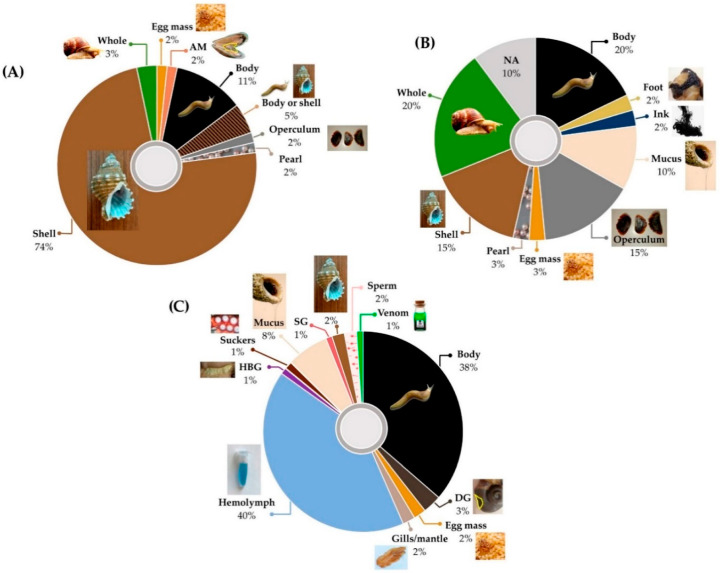
Molluscan body parts used to derive extracts/compounds in (**A**) Traditional Chinese Medicines (n = 61 marine remedies; [104] Table S2), (**B**) other traditional medicines (n = 39 remedies; Table S1) (**C**) in vitro, in vivo and clinical biomedical studies (n = 97 articles; Table 5, Table 6, Table 7 and Table 8 and Table S3; not including studies using Hc as a model antigen Table S4). NA: not available, AM: adductor muscle, HBG: hypobranchial gland, DG: digestive gland, SG: salivary gland; shell includes cuttlebone; whole includes those listed as “whole animal” or “body and shell”.

Molluscs are a valued resource providing food, dye, shells, forming currencies and medicines, as well as being of symbolic and ritualistic importance to many historical and contemporary cultures around the world [90,106,107,108]. Respiratory diseases have always concerned human health and sophisticated traditional medicine systems formed the basis of care long before the advent of modern pharmaceuticals. Many traditional respiratory medicines feature diverse preparations of the flesh, shell, opercula, eggs and secretions of different mollusc species, mostly sourced from local marine environments (Table 2, Table 3 and Table S1, Figure 1). Records of molluscan respiratory medicines date back at least as far as Ancient Greece (800 BC), when the boiled flesh of *Octopus vulgaris* was used to relieve heavy nasal congestion with fever, treat infectious diseases, and strengthen the body’s immune system [109] (Table S1). During the Middle Eastern Medieval Period (500–1500 AD), the sea hare *Aplysia depilans* was used in traditional Arabic medicine to alleviate dyspnea (difficult or labored breathing), dry cough, and hemoptysis (coughing up blood) (Table S1). Traditional Chinese Medicine (TCM) is considered one of the oldest (>3500 years old) and most well-documented systems of traditional medicine in the world [49]. Most TCMs are derived from plant/terrestrial natural products, although marine species are important with an entire volume dedicated to marine-derived remedies [104]. Of the marine TCMs, 111 are derived from molluscs and 61 of these are used to treat various respiratory ailments [104] (Table S2). References to two additional molluscan (1 marine, 1 terrestrial) TCMs that are not listed in the *Marine Materia Medica* [104] but are used for respiratory disease were found [110,111] (Table S1).

Several recurrent themes emerge regarding the application of molluscan traditional medicines for certain respiratory diseases (Table 2). Many are indicated for the treatment of tuberculosis (TB), both pulmonary and extrapulmonary (Table 2, Tables S1 and S2). The disease has been a permanent challenge over the course of human history, given the primordial origins of *Mycobacterium tuberculosis* [112] and it stands to reason that traditional treatments for TB infection (and the cough and inflammatory symptoms it causes) are ubiquitous. Alves et al. [113] documented the use of bivalve mollusc flesh and shells used to treat TB throughout South America; in India, the flesh of marine *Turbinella* sp. and freshwater *Plia globosa* is cooked and eaten [114,115]; “snail water” and “snail syrup” prepared from terrestrial *Helix* sp. were prescribed in Europe since the 1700s (Lemery, 1738 in [91]) and decoctions of numerous marine mollusc species occur in TCM (Tables S1 and S2). Traditional molluscan medicines are also indicated for other infectious respiratory diseases, including influenza, pneumonia, bronchitis, measles, otitis media, and general respiratory tract infections (Table 2, Tables S1 and S2) and biomedical studies have targeted responsible pathogens (Table 4, Table 5 and Table S3). Two biomedical studies have shown good in vitro activity of molluscan compounds against *M. tuberculosis*– analogues of kahalalide F [116] from a tropical sea slug, and lobophorins from *Streptomyces* sp. associated with a gastropod from the Philippines [117]. However, in both cases, effective concentrations exceed toxicity estimates, rendering these compounds unsuitable as therapeutic candidates against *M. tuberculosis* without modification to reduce toxic side effects (Table 5). Non-tuberculosis mycobacteria (NTM) infection (arising from mycobacteria other than *M. tuberculosis*) is a clinically severe and rapidly increasing problem worldwide (Table 1) [28]. No reference to this disease was found in traditional or contemporary literature and it should be a subject of further investigation.

**Table 2:** Summary of traditional molluscan medicines used to treat respiratory diseases or symptoms. Details available in Supplementary Tables S1 and S2.

Respiratory Disease or Symptom	Words/Phrases Used in the Literature to Describe Symptom or Disease	No. of Remedies *	No. of Species	Mollusc Parts Used	Cultures/Traditional Medicine Systems	Ref.
Allergy	Allergy; hypersensitivity; ENT or pulmonary allergies	10	2	Egg masses; flesh; whole animal; ink; shell	Europe; China	[104,118,119,120]
Asthma	Asthma; shortness of breath; dyspnea; wheeze; asthmatic cough; dyspnea with cough	19	46	Body; foot; shell; pearl; eggs	China; India; South America; Middle East	[78,91,110,113,114,115,118,119,120,121,122,123,124,125]
Cancer	Cancer; tumor; neoadjuvant treatment	4	17	Flesh; shell; operculum	India; South America; Egypt; China	[104,113,114,126,127]
Cough	Cough; chesty cough; croup; hemoptysis; laryngismus; whooping cough; cough associated with infection or fever; cough associated with inflammatory conditions; nervous cough; cough with chest stuffiness and dyspnea; xeropulmonary cough; cough and regurgitation	28	127	Adductor muscle; egg masses; flesh; mucus; pearl; shell	China; Europe; India; South America; Middle East; Nigeria	[91,113,114,123,124]
Ear problems	Ear problems; ear pain; ear inflammation; ear ache; ottorhoea; otitis media; parotid gland swelling and hearing loss; ear and eye diseases	9	14	Flesh; mucus; shell; operculum	China; Europe; India; Egypt; Nigeria	[104,109,114,118,119,120,123,126,127]
Fever	Fever; high fever; low fever; fever in children; fever and convulsion in children; high fever; feverish sensation in chest; night sweating; heat; heat toxicity	15	59	Adductor muscle; flesh; pearl; shell; whole animal	China; Europe; India; Korea	[91,104,109,114,128]
Low immunity	Strengthens immune system	3	3	Flesh; shell	Europe	[109]
Infection †	Infection; pneumonia; measles; flu; bronchitis; anthrax; upper respiratory tract infections in children; infectious diseases; bronchitis; measles; conjunctive congestion with swelling and pain	18	56	Flesh; shell; mucus; whole animal	China; Europe; South America; India	[91,109,113,114,118,119,120,129]
Respiratory inflammation	Inflammation; sinus inflammation; inflammatory conditions; parotid gland swelling; acute and chronic chest ailments; edema; swelling and pain; acute and chronic sinusitis	10	36	Flesh; shell; mucus; whole animal; operculum	China; Europe; India	[91,109,114,123]
Mucus	Mucus; excessive mucus; phlegm; congestion; nasal congestion; used as expectorant; retention of phlegm and fluid; phlegmatic heat; retention of fluid in chest	22	101	Adductor muscle; flesh; operculum; pearl; shell	China; Europe; India	[109,114,118,119,120]
Sore throat	Sore throat; pharyngitis; hoarseness; tonsillitis; tracheitis; pharynalgia	10	21	Flesh; mucus; shell; whole animal; pearl	China; Europe; South America; India	[91,114,130]
Tuberculosis ‡	Tuberculosis; pthisis; scrofula; pulmonary tuberculosis; tuberculosis of lymph nodes	47	237	Adductor muscle; egg masses; flesh; shell; mucus; whole animal; pearl	China; Europe; India; South America	[91,113,114,115]
Other ^§^	Chest and abdomen heat and pain; pain in sternum; bleeding from five aperture or subcutaneous tissue (e.g., eye; ear; nose; teeth; tongue)	6	35	Flesh; shell; pearl	China	[104]

* Containing at least one, usually several different, mollusc species; one remedy may be used to treat various conditions in which case it has been counted more than once; a remedy is included once if various words/phrases were used in the cited text relating to the same condition. † Not including tuberculosis. ‡ Pulmonary and extrapulmonary. § Included if remedy used for at least one specified respiratory condition.

Asthma represents another universal respiratory disease, albeit of a completely different nature to TB, being non-communicable and a function of inherent immune dysregulation, with both genetic and environmental factors (Table 1). Records of eight TCMs and 19 other traditional molluscan medicines refer to the treatment of asthma (Table 2, Tables S1 and S2). Notable ones include tea of toasted cuttlefish bones or octopus arms taken among traditional Brazilian communities [122], soup prepared from the foot of freshwater snail *Filopaludina* sp. in India, and preparations of mollusc flesh eaten in China [78], India [114], and South America [113] (Table S1). 

Most TCMs and other traditional medicines are applied for multiple, possibly related, respiratory conditions (Table 2, Tables S1 and S2). For example, the TCM “Yan Qiu Bei” derived from the decocted shell of the cowrie *Naria erosa* (Cypraeidae) is used for extrapulmonary TB, shortness of breath, excessive mucus, and conjunctive congestion [104] (Table S2), while Indian remedies using *Turbinella* sp. are not only used for TB, but for cough, excessive mucus, sore throat, fever, earache, and asthma as well [114] (Table S1). It must be noted that most of the TCMs and other traditional medicines used for respiratory diseases (Tables S1 and S2) are commonly used for other, non-respiratory conditions (e.g., gynecological problems, gastrointestinal disorders and cardiovascular diseases), which may reflect general anti-inflammatory and anti-angiotensin properties. 

There are few references to cancer in general, or respiratory cancer specifically, among traditional molluscan medicines (Table 2, Tables S1 and S2). Those in this review were included because they are/were also used for at least one other definitively respiratory condition: *Crassostrea rhizophorae* flesh and shell used in South America [113] for cancer, TB, flu and pneumonia, opercula of the *Muricid* mollusc *Chicoreus virgineus* used in Medieval Egypt for tumor and eye/ear diseases [126,131], and the TCM “Mu Li” comprising the flesh and shell of 12 Ostreidae/Grypaeidae sp. for cancer and TB [104] (Table 2, Tables S1 and S2). Descriptions of the preparation and application of each of these medicines is unavailable or ambiguous (Tables S1 and S2), in alignment with Cragg et al. [93,132] who advises that claims for cancer treatments should be viewed with some skepticism because the disease is likely to be poorly defined in traditional medicine. Respiratory cancer, insidious and inconspicuous by nature, may be masked by other symptoms described in the literature (e.g., pneumonia, hemoptysis, shortness of breath, tightness or fullness in chest; Tables S1 and S2). Nonetheless, there exists plenty of biomedical evidence regarding the anticancer activity of molluscan extracts and compounds, some with specificity to respiratory cancers, discussed later in this review (Table 4, Table 6, Table 7 and Table 8).

Among different traditional medicine systems, certain molluscan families, species and body parts are commonly used suggesting shared properties. For example, mucus of terrestrial snails has been widely consumed as a cough remedy with records of use in Africa (*Limicolaria aurora*, *Lanistes ovum*) [123], China (*Limax* sp.) [78], and throughout European history (reviewed by Bonnemain [91]) (Tables S1 and S2). Intertidal periwinkles (Littorinidae) are used in South America [123] and Africa [123] for symptoms of respiratory inflammation (Table S1). Different parts of the cuttlefish *Sepia officinalis* were used in Ancient Greece [109] and more recently in India [114] and Europe [120], while the remedy “Sepia”, manufactured from *S. officianalis* ink [120] has been part of the *Homeopathic Materia Medica* since the 1800s [133]. However, this and other homeopathic remedies are typically prescribed at dilutions of 6C or 30C (equivalent to 10^−12^ or 10^−60^, respectively). At such high dilutions the presence and bioactivity of the original compounds is conceivably negligible [90]. 

The shell (and whole body, including shells) is the molluscan part most frequently used in traditional medicines for respiratory diseases (Table 2, Tables S1 and S2; Figure 1). In TCM, shells are ground into powder or decocted (heated to extract essence) and ingested (Table S2). Ashes of burned mollusc shells or cuttlefish bones, prepared into pills, pastes, and solutions, feature in several traditional respiratory medicines originating in India [114] and Ancient Greece [109]; shells are also used in South American medicine [113,121] and homeopathy [118,119,120]. Only three biomedical studies have investigated shell extracts with relevance to respiratory disease: powdered cowry (*Monetaria moneta*) shell [134] and chitosan from cuttlefish (*S. officianalis*) bone [135,136], both which show antimicrobial properties in vitro (Figure 1; Table 5). 

**Figure 2:**
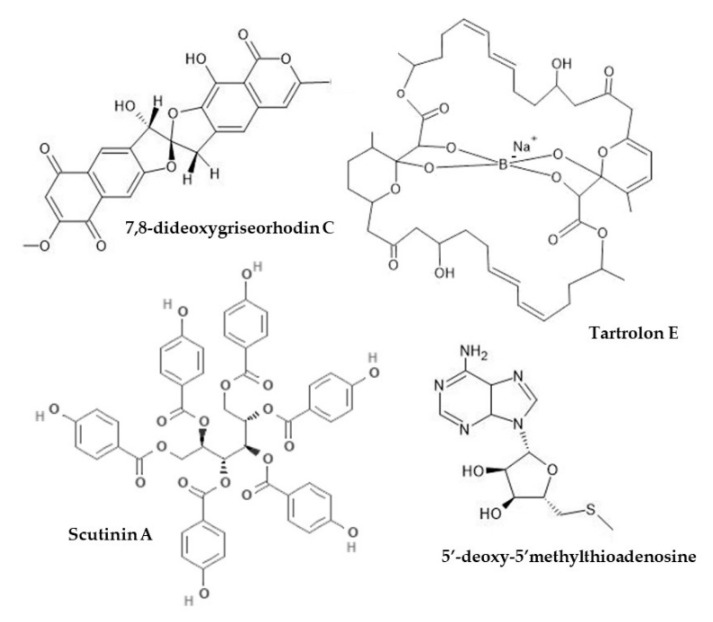
Examples of molluscan compounds with defined chemical structures showing antimicrobial activity against respiratory bacteria. Produced with information in [169,171,172,176].

**Figure 3:**
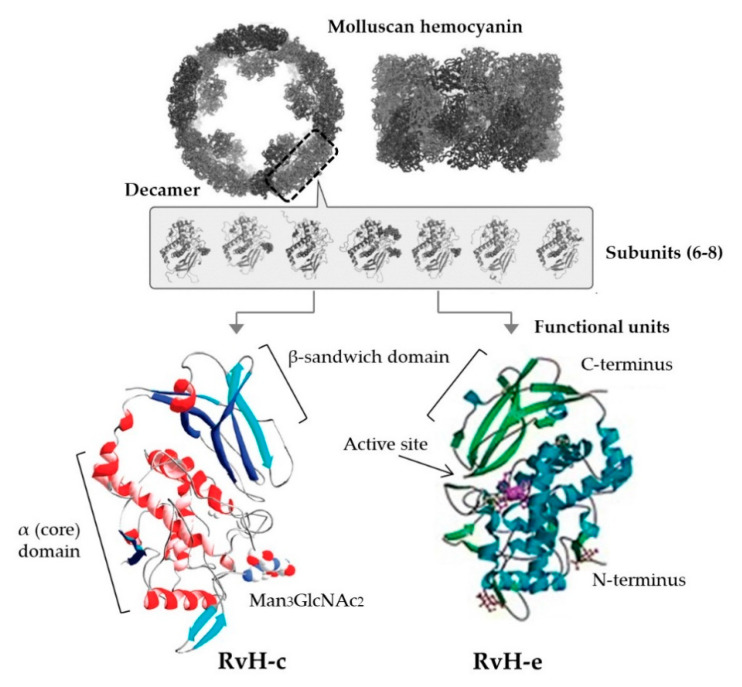
General structural levels of molluscan hemocyanins and two functional units (c and e) of deoxygenated hemocyanin from *Rapana venosa* (RvH) (Mollusca: Muricidae; originally listed as *R. thomasiana*) with bioactivities (antiviral, anticancer, immunomodulatory) relevant to respiratory disease. Adapted from [76,197,199].

The shell-less body (or “flesh”, and “whole animal” including the body) is the second most utilized molluscan part in traditional medicines for respiratory diseases of every nature (Table 2, Tables S1 and S2; Figure 1). Flesh may be cooked and eaten [109,115] or applied externally [104,109] (Tables S1 and S2). In Ancient Greece, flesh of cephalopods (*O. vulgaris, S. officianalis*) and the giant trumpet shell (*Charonia tritonis*) were eaten for conditions of low immunity in addition to infectious or inflammatory respiratory diseases [109] (Table S1). Among Nigerian tribes, the flesh is “punctured to obtain the fluid” ([123] p. 491). These accounts could be related to circulating hemolymph (equivalent to blood), which represents at least 20% of molluscan body weight [137]. Hemolymph is composed largely of hemocyanin (Hc), which has a range of bioactivities and is among the most potent of immunogens used in respiratory disease-related studies, as discussed later in this review (Tables S3 and S4). Many other bioactive compounds (e.g., AMPs, proteins, peptides, and polyketides) have been derived from molluscan body tissue (Table 5, Table 6, Table 7 and Table 8 and Table S6, Figure 2 and Figure 3).

### 3.2. Supporting Evidence for the Bioactivity of Traditional Molluscan Respiratory Medicines

The bioactivity and therapeutic value of traditional molluscan medicines is for the most part speculative. However, there are growing bodies of substantiating evidence by virtue of biomedical studies using derivatives of hemolymph, mucus, body extracts, and specialized glands (Figure 1, Table 3, Table 4, Table 5, Table 6, Table 7 and Table 8). European 18–19th century physicians and pharmaceutical texts proclaimed the unsurpassed benefits of helicidine (mucus of the terrestrial snail *H. pomatia*) for alleviating various respiratory conditions such as whooping cough, TB, influenza, pneumonia, chronic bronchitis, and asthma [91] (Table S1). In 1953, Quevauviller [138] reviewed the pharmacology of helicidine relating its effectiveness to demonstrated sedative, mucolytic and bacteriolytic activities, and in 1999, Pons et al. [79] established that the broncho-relaxant effect of helicidine in vitro was mediated by the release of prostaglandin E_2_ and inhibited by pre-treatment with a cyclooxygenase (COX) inhibitor, indomethacine (Table 6). A double-blind, placebo controlled clinical trial followed in 2001 [80] whereby 30 patients with COPD were treated with daily doses of helicidine (10%) over five days, resulting in a significant reduction in nightly coughing episodes (4.7–5.1 pre-treatment, 2.7–4.9 placebo, 1.3 helicidine group) and duration of coughing periods (Table 8). 

Other terrestrial land snails, of the *Limax* genus, originally appeared in the Chinese *Compendium of Materia Medica* in 1578 and continue to be used in TCM for respiratory wheeze, phlegm, and pharyngitis [78] (Table S2). In a recent robust murine model of cigarette-smoke induced COPD undertaken by Liang et al. [78], aqueous body extract of *Limax* sp. significantly improved pulmonary function and reduced key inflammatory mediators (Table 7). The effect is attributable to enhancement of peroxisome proliferator-activated receptor-γ (PPAR-γ), which acts to downregulate pro-inflammatory transcription factors and mucin synthesis, and suppression of p38 and ERK1/2 mitogen-activated protein kinase (MAPK) pathways, which activate the production of pro-inflammatory mediators [17], and also play a role in malignant transformation [139], in structural and immune cells of the lung. In other models, systemically administered *Limax* sp. powder-water suspensions have been shown to inhibit Lewis lung carcinoma growth in mice [111], and reduce the onset of asthma symptoms and production of inflammatory markers in guinea pigs more effectively than the clinical bronchodilator Aminophylline [140] (Table 7).

**Figure 4:**
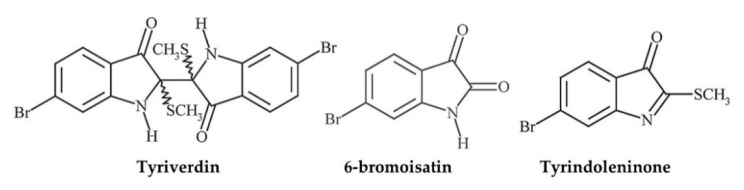
Examples of brominated indole/isatin derivatives showing anti-inflammatory, anticancer, and antimicrobial activity relevant to respiratory disease.

The flesh and shell of Muricidae sp. occur in eight TCMs [104] and their operculum were ingested as a medicinal oils in Ancient Greece [109], Ancient India [126,131], and Medieval Egypt [126,127] for various infectious and inflammatory respiratory diseases, and cancer (Table 2, Tables S1 and S2). Considerable attention has been devoted to Tyrian purple (6,6′dibromoindigo and related compounds), a historically important textile dye obtained from the Muricidae family, which was first described in the *Historia Naturalis* (1669) [141,142,143,144,145]. Natural product research has since ascertained the broad spectrum antibacterial, anti-inflammatory, and anticancer activity of Tyrian purple precursor compounds, minor pigments, and modified derivatives [49,53,75,77,141,146,147,148]. Extracts of an Australian whelk *Dicathais orbita* containing these bioactive compounds have shown preliminary antimicrobial activity against respiratory pathogens in vitro [149] and anti-inflammatory activity in a murine model of acute lung inflammation [77] (Table 7). Some bioactive compounds are also detectable in lipophilic extracts of *D. orbita* operculum [126], with relevance to ancient medicinal oil preparations [127,131] (Table S1). Nonetheless, the only Muricidae sp. that have been the focus of investigations pertaining directly to respiratory disease are *D. orbita* (brominated compounds) [77] and *Rapana venosa* (Hcs) [76,150] (Table 7 and Table S3, Figure 3 and Figure 4), while at least 30 different Muricidae sp. are used in traditional respiratory medicines (Tables S1 and S2).

### 3.3. Taxonomic and Geographic Trends

The majority of traditional medicines and biomedical studies are based on extracts and compounds from the Gastropoda and Bivalvia (Table 3), which is unsurprising given that these classes comprise the majority (>90%) of molluscan biodiversity [53]. There are 21 families of Bivalvia used in traditional medicines that have not yet been investigated scientifically. Only one study has investigated the anti-inflammatory activity of Cephalopoda extracts [151], despite their widespread traditional use as anti-inflammatory medicines [109,120,121,122] (Tables S1 and S2). The remaining four classes are all marine and relatively minor in terms of their ecological diversity and representation in both biomedical and ethnomedical literature (Table 3) [53]. However, the Polyplacophora (chitons) are of research interest, also noted by Benkendorff [53], given their ecological abundance and use in TCM for asthma, TB, and bronchitis (Table S2). Chitons also form important South African traditional medicines, but not for respiratory disease [108]. The Scaphopoda (tusk shells) represent a unique evolutionary avenue within the Mollusca [152]; they are the only class of exclusively infaunal molluscs and could yield interesting chemistries but remain poorly examined to date (Table 3) [152]. The Aplacophora and Monoplacophora mostly occupy the deep-sea making for challenging access and limited utility, although such extreme adaptive radiation in their biology suggests that they are also likely to be chemically unique [53]. 

**Table 3:** Number of different mollusc families by taxonomic class and habitat represented in traditional medicines (Traditional Chinese Medicines [TCMs]; other traditional medicines [OTMs]) and biomedical studies in which molluscan extracts/compounds have been used for the treatment or investigation of respiratory disease.

	Number of Different Mollusc Families Represented in Each Literature Type					
	Traditional Medicines		Biomedical Studies			
	TCMs *	OTMs	In Vitro	In Vivo	Clinical Trials	Model Antigen †
Mollusc class						
Gastropoda	20	15	49	7	3	2
Bivalvia	23	6	9	3	1	0
Cephalopoda	1	3	4	0	0	0
Polyplacophora	2	0	1	0	0	0
Aplacophora	0	0	0	0	0	0
Monoplacophora	0	0	0	0	0	0
Scaphopoda	0	0	0	0	0	0
Habitat type						
Marine	46	17	55	8	3	1
Freshwater	0	3	2	0	0	0
Terrestrial	0	4	5	2	1	1

* Literature based on marine sp. only [104]. † Hc used as a model antigen in in vivo models and clinical trials.

Regional environmental factors have important bearings on the chemical and biological diversity of mollusc species used as traditional medicines in different cultures. A large proportion of traditional medicines included in this review represent Asian (52%), European (24%), and South American (5%) cultures and species endemic to these regions. By comparison, biomedical research efforts have been focused in Europe (34%), North America (28%), and Asia (China and India, 27%). Only one South American species has been studied [153,154] and Australian species are also underrepresented (Table S1). Numerous sources attest to the importance of molluscs as traditional food sources as opposed to traditional medicines in Australia and throughout the Indo-pacific [155,156]. It is possible that molluscs are also used for medicinal purposes but the knowledge has been retained by Indigenous communities or otherwise lost. Accordingly, species from these regions do not appear in our review of traditional medicines, but are nevertheless likely to have some nutraceutical qualities equivalent to molluscs found elsewhere.

Respiratory diseases have changed over time and modern society now faces pathogens and environmental hazards once unimagined [33]. Nonetheless, traditional medicines have not lost their place and continue to provide stimulus for research. Those included in this review incorporate over 300 different mollusc species (Tables S1 and S2), while only 93 species have been investigated in biomedical studies to date (Tables S3–S8). It is estimated that 80% of people in developing countries still rely on traditional medicines as a primary source of healthcare [157] and well-known systems, including TCM, Indian Ayurveda and homeopathy, are expanding in global popularity throughout the developed world, despite a lack of robust evidence for their safety and efficacy [157,158,159]. Further testing is needed to substantiate the use of traditional molluscan medicines and identify bioactive compounds that could serve as APIs and novel drug leads for respiratory disease.

## 4. Chemistry, Bioactivity and Biomedical Applications of Molluscan Extracts and Compounds Relevant to Respiratory Disease

### 4.1. Overview

At least 97 peer-reviewed biomedical articles published mostly within the past two decades investigate the bioactivity of molluscan extracts and compounds with direct relevance to the treatment, prevention or understanding of respiratory disease (Table 4). A small proportion (less than 2%) of the 327 individual extracts/compounds tested in vitro have progressed though in vivo models of respiratory disease to clinical trials (Table 4). Only two have been approved for use although at least 12 have been patented for development toward respiratory disease (Table S5). Fewer than half of studies have used purified compounds (Table S6). Those compounds that have been purified include polyketides (e.g., Figure 2), proteins, glycoproteins (e.g., Figure 3), PUFAs, and brominated indole/isatin derivatives (e.g., Figure 4) (Table S6).

The in vitro studies included in this review are focused on antimicrobial properties, usually expressed as a minimum inhibitory or bactericidal concentration (MIC or MBC, respectively), representing the minimum concentration of an extract/compound required to inhibit the growth of microbial cells or kill them entirely; and, anticancer properties whereby the indexes IC_50_, EC_50_, GI_50_ or CC_50_ (50% inhibitory concentration, effective concentration, growth inhibition or cytotoxic concentration, respectively) are used often and interchangeably, representing the concentration of an extract/compound that reduces cell growth in vitro by 50% compared to the untreated control. In vitro studies have used extracts/compounds derived from the hemolymph, sperm, tissue associated with symbiotic bacteria, shell, salivary gland, digestive gland, egg mass, body, and mucus of different mollusc species (representing 56 families) (Figure 1, Table 3). Further testing and characterization of crude extracts used in many of these studies may lead to the identification of novel bioactive lead compounds, which could become productive drug leads (Table 5 and Table 6).

In vivo studies are based on mouse models of respiratory disease (COPD, allergic and acute inflammatory airway disease, lung cancer and respiratory infection) treated with molluscan extracts/compounds. More than half (13) of these models use Hc for its immunogenic properties in vaccine preparations targeting respiratory pathogens (Table S3). Another three extracts and 10 compounds are derived from the body, hemolymph, or hypobranchial gland of different mollusc species with a focus on their anti-inflammatory and anticancer properties (Table 4 and Table 7). A separate body of research uses molluscan Hcs as a model antigen in respiratory disease-related immunological studies (Table S4). The focus of these studies is not on the therapeutic value of Hc, rather they provide insight into the bioactivity, safety, and biomedical applications of Hc and functional units.

We identified 11 human clinical trials using purified molluscan compounds as treatments for respiratory disease (Table 4 and Table 8). These trials have used keyhole limpet hemocyanin (KLH) as a vaccine adjuvant/conjugate (n = 4) (Table S3), kahalalide F (and derivatives) for lung cancer (n = 3), helicidine for COPD (n = 1), and Lyprinol for asthma (n = 3) (Table 8). Lyprinol is now FDA approved for use as an alternative anti-inflammatory agent, and helicidine-containing cough medicines have been available over-the-counter in European pharmacies for over 50 years [160]. The overall benefits of other clinically tested molluscan compounds have been marginal (Table 8) such that they are being further developed with structural modifications (Table S5).

**Table 4:** Activity of molluscan extracts and compounds with relevance to respiratory disease reviewed across 97 biomedical publications.

Type of Study	In Vitro	In Vivo Models	Clinical Trials	Model Antigen *	Total Studies	% of Studies
No. of studies	54	25	11	16	106 †	
No. of compounds/extracts ‡	327	15	5	3		
No. of studies reporting bioactivity ^§^						
Anticancer	13	7	3		23	22
Antibacterial	33	1			34	32
Antiviral	4				4	4
Antifungal	7				7	7
Anti-inflammatory ^‖^	1	5	3		9	8
Antitussive	1		1		2	2
Immunogenic ^¶^	2	15	4	16	37	35

* Includes both in vivo animal models and human studies using molluscan hemocyanin as a model antigen as opposed to treatment. † Of the 97 biomedical articles, nine in vivo animal models include substantial in vitro components which are presented separately in this table, hence 105 total studies. ‡ Experimentally purified and laboratory grade hemocyanin considered different; some compounds represented in in vivo studies and clinical trials may not be represented in in vitro studies in this table as assays may have been less specific to respiratory disease. ^§^ Number of studies reporting bioactivity may exceed the total number of studies as some report >1 type of bioactivity. ^‖^ General anti-inflammatory activity in vitro is underrepresented in this table as assays are less specific to respiratory disease, but still relevant; see Ahmad et al. [49]. ^¶^ Vaccine conjugate/adjuvant or immune stimulant.

### 4.2. Antimicrobial Activity

Research on molluscan antimicrobial chemistry and immunology has been driven mainly by development of the commercial aquaculture industry and microbial disease outbreaks among major cultured mollusc species (e.g., oysters, abalone and mussels [161,162,163,164]). However, the bioactivity of molluscan extracts and compounds is not strictly limited to molluscs and the pathogens that affect them. Antimicrobial susceptibility test suites often include bacterial, viral, and fungal pathogens responsible for common respiratory infections in humans (e.g., [65,73,136], Table 5). The prevalence, intrinsic virulence, and progressive antimicrobial resistance of respiratory pathogens continues to drive research interest and some novel molluscan compounds show promising bioactivity. Even so, most antimicrobial work has only been performed in vitro, and to describe a compound or effect as “promising” based on this data alone is to forget that a disease in a living organism is much more complex; hence, the need for in vivo testing. *Pseudomonas aeruginosa* is particularly associated with chronic bacterial infections in COPD, cystic fibrosis, and conditions of compromised immunity (Table 1) and its treatment is complex because of recurrence and multi-drug resistance [165,166]. Given the clinical need, *P. aeruginosa* is commonly included in antibacterial assays (Table 5; 23 studies), although activity against it is generally weak due to the protective lipopolysaccharide outer cell membrane characteristic of this and other Gram-negative bacteria [73,167,168] (Table 5). Reasonable antibacterial activity against *P. aeruginosa* has been observed using some molluscan extracts and compounds (Table 5). Examples include: tyriverdin (MIC 0.005 mg/mL) sourced from egg masses of *D. orbita* [149]; tartrolon E (MIC 0.31 mg/mL) a polyketide isolated from a molluscan gill symbiont [169]; peptides from *O. vulgaris* (MIC 50–300 μg/mL) [170]; Scutinin A isolated from Australian limpet *Scutus antipodes* (MIC 33 μM) [171]; 5’-deoxy-5’-methylthio-adenosine (MTA) from a dorid nudibranch [172]; and body extract of *Drupella margariticola* (MIC 0.07 mg/mL) [173] (Table 5, Figure 2 and Figure 4). Preliminary assays using mucus of *H. aspera* [174] and crude extract of *Babylonia spirata* [175], show antimicrobial activity although weaker than antibiotic controls (Table 5). Notwithstanding, crude extracts would sensibly become more effective if the active compound/s were concentrated by purification. Hence, extracts that show weak activity should not necessarily be overlooked in case the active factor is a minor component.

The capacity to form biofilms (structured communities of bacteria encapsulated within an extracellular polymeric matrix) confers some bacteria, including *P. aeruginosa*, an enormous advantage in establishing and maintaining infections in the respiratory tract [177]. Of interest therefore are studies by Gasu et al. [178,179] using peptide extract of *Olivancillaria hiatula* establishing a relatively low MIC of 0.039 mg/mL against *P. aeruginosa* and 50% inhibition of biofilm formation at the same concentration, though 2.5 mg/mL was required to degrade pre-formed biofilm (Table 5). Further, the peptide extract reduced the expression of virulence factors (pyocyanin, pyoverdine, and protease) by >50% at 0.0195 mg/mL and acted synergistically with standard antibiotics ciprofloxacin and cefotaxime (up to 100% reduction in MICs) (Table 5). As well, Maselli et al. [170] recently reported antibiofilm activity (60% eradication at 80 μM) among peptides derived from the suckers of *O vulgaris*. Preliminary evidence indicates that Hcs from marine crustaceans can also inhibit biofilm formation (at 0.1 mg/mL) [180,181,182] and molluscan Hcs could function similarly.

Community-acquired pneumonia is the leading cause of death from infection worldwide, particularly in infants, and is typically caused by *Streptococcus pneumoniae* [2,183]. Bacteriostatic activity against *S. pneumoniae* has been observed using crude body extracts of *D. margariticola* (MIC 0.07 mg/mL) [184], *Babylonia spirata* [175], and several Cephalopods at high concentrations [185]. Other molluscan extracts/compounds (e.g., Helicidae sp. Hcs [73] and Muricidae sp. brominated compounds [149]) showing activity against Gram-positive bacteria could be investigated targeting this pathogen.

Methicillin-resistant *Staphylococcus aureus* (MRSA) is a common cause of troublesome healthcare-acquired infections, including pneumonia [186]. Bacteriostatic effects on MRSA have been observed using compounds isolated from molluscan bacterial symbionts [169]. The recent work of Miller et al. [176] using 7,8-dideoxygriseorhodin C (DC) is notable: MIC values for DC (0.08–0.12 µg/mL) were lower than the control antibiotic oxacillin (1.59–6.24 µg/mL), and both the number of colony-forming units and MICs for DC and oxacillin were reduced >100 fold (i.e., synergistic effects) when present in combination (Table 5, Figure 2). Extracts from the sperm of the Mediterranean mussel (*Mytilus galloprovincialis*) have displayed bactericidal activity against clinical *S. aureus* strains, as well as negligible toxicity and resistance to acid digestion (i.e., suitable for oral administration) [187]. Wei et al. [188] prepared two recombinant proteins (rSgSABL-1 and -2) from lectins of the razor clam *Solen grandis,* which exhibited strong binding affinity to *S. aureus* peptidoglycan, increased phagocytic and encapsulation activity in vitro, and generated reactive antibodies in vivo (Table 5 and Table 7). Aside from Wei et al. [188], the antimicrobial activity of molluscan extracts/compounds against respiratory pathogens has not been tested in animals or humans aside from the inclusion of Hcs in vaccine preparations (Table S3).

Terrestrial mollusc mucus is a mixture of proteoglycans, glycoprotein enzymes, AMPs, and other minor constituents in ~90% water [189]. Preliminary antibacterial results from disc diffusion assays using some respiratory pathogens are presented by Cilia and Fratini [189], but statistically meaningful data are yet to be derived. de Toledo-Piza et al. (2016) demonstrated impressive anti-viral activity with concentrated mucus from the terrestrial shell-less mollusc *Phyllocaulis boraceiensis* whereby host cell pre-treatment reduced Influenza A (H1N1) and measles virus-induced cytopathic effects by up to 80% with no host cell cytotoxicity [153,154]. Further studies should incorporate bioassay guided fractionation of concentrated mucus extracts to isolate and identify the active factors.

Numerous published reviews discuss Hcs and AMPs as effectors of invertebrate immunity and their potential as antibiotic leads [102,190,191,192,193,194,195]. Regardless of their biological origin, all AMPs are small in size, often cleaved from Hcs, with either a cyclic (encompassing amphipathic surfaces) or linear (containing di-sulfide bridges) structure and broad-spectrum antimicrobial activity [164,193] (Figure 3). Molluscan Hcs and AMPs show some inhibition of respiratory bacteria (mostly Gram-positive) and fungi [73,167,168] (Table 5). Although, they may hold even greater potential as antivirals. The review by Dang at al. [56] presents evidence for antiviral effects of molluscan Hcs and AMPs against a range of human viruses including herpes simplex virus, Epstein-Barr virus (double-strand, lipid-enveloped DNA), simian rotavirus (double-strand, non-enveloped RNA) and poliovirus (single-strand, non-enveloped RNA) [56]. Regarding respiratory disease, virucidal activity has been shown using oyster hemolymph tested against human adenovirus (AdV-5) (EC_50_ 0.05–0.09 mg/mL, cytotoxic at 0.19–0.36 mg/mL) [196] and with Hc subunit c (RvH-c) from *R. venosa,* which inhibited the cytopathic effects of respiratory syncytial virus (by 71.4% at 1 mg/mL) and indicated that some Hc structural units may be more bioactive than others [197] (Table 5, Figure 3). Nonetheless, negligible toxicity toward healthy eukaryotic cells and low potential for the development of resistance [164,198] are clear advantages for the inclusion of Hcs and AMPs in various antimicrobial and anticancer preparations.

Molluscs provide good bioavailable sources of elemental zinc [200]. Zinc was recently shown to inhibit the replication of respiratory viruses (including influenza and COVID-19), reduce host cell infection, improve immune function, and act synergistically with standard antiviral therapies [201,202,203]. Antiviral factors isolated from molluscs could therefore be combined with zinc for improved activity. As well, extracts from whole molluscs, bodies, and shells used in traditional medicines likely contain zinc, which may be in part responsible for some derived benefits (Tables S1 and S2).

### 4.3. Anti-Inflammatory Activity

Scientific and epidemiological evidence suggest a beneficial relationship between dietary PUFA consumption and prevention or alleviation of chronic diseases including asthma, allergic airway disease, cardiovascular disease, and cancer [204,205,206]. Lyprinol™ is a patented extract of the New Zealand green lipped mussel (*Perna canaliculus*) known to inhibit 5′-lipoxygenase and COX pathways responsible for the production of inflammatory eicosanoids, among other less well-characterised involvement in the production of resolvins and protectins [207]. In a murine model of allergic airway disease, Wood et al. [208] found that daily gavage with 100 µL Lyprinol™ significantly reduced eosinophil influx, mucus hypersecretion, and airway hyperresponsiveness (Table 7). Indeed, different forms of marine oils have different bioactivities since they contain different types and amounts of PUFAs as well as a variety of lipid mediators [207]. Lyprinol™ contains a mixture of PUFAs (including eicosapentaenoic acid [EPA], docosahexaenoic acid [DHA] and other n-3 PUFAs), triglycerides, sterol esters, sterols, and polar lipids combined with olive oil and vitamin E, and these multiple nutritional components may contribute to the superior effectiveness of the supplement over fish oil in animal models [208] (Table 7) and clinical trials (e.g., [209,210] [Table 8] compared with [211]).

Ahmad et al. [212] first established evidence for the anti-inflammatory activity of hypobranchial gland (HBG) extract and 6-bromoisatin (among other related compounds) from *D. orbita* based on the in vitro inhibition of pro-inflammatory mediators (NO, TNF-α and NFκB) in mouse cell lines (Figure 4). In this study, effective, non-toxic concentrations were <50 µg/mL and the purified mono-brominated indole and isatin compounds (i.e., 6-bromoisatin) were more bioactive than their non-brominated counterparts and crude HBG extract [212]. Ahmad et al. [77] was led to investigate whether these anti-inflammatory effects could be replicated in a model of acute lung inflammation, in which HBG extract and 6-bromoisatin significantly inhibited the inflammatory pathway (TNF-α and IL-1β, and neutrophil infiltration) and prevented associated lung tissue damage (Table 7, Figure 4). No mortality, ill-health, liver toxicity, or gastro-intestinal damage was observed in this model, nor others (non-respiratory) using the same extracts/compounds administered over 2–14 weeks (0.05–0.1 mg/g) [48,77,213,214,215]. Evidently, brominated compounds from *D. orbita*, and other Muricidae sp., could prove especially valuable for their anti-inflammatory activity and other properties relevant to respiratory disease.

Very few investigations of molluscan extracts and compounds are specific to inflammatory respiratory diseases, regardless of all the traditional uses of molluscs relevant to symptoms of inflammation (Table 2, Tables S1 and S2; [49]) and the prevalence of these conditions (e.g., COPD, asthma, ARDS; Table 1). Those extracts/compounds that have been tested specifically for inflammatory respiratory conditions include: helicidine, studied in vitro with relevance to asthma [79] (Table 5) and in a clinical trial for COPD [80]; *Limax* sp. extract, studied twice in vivo for COPD [78] and asthma [140]; *muricid* sp. brominated compounds, studied in vivo for acute lung inflammation [77]; and Lyprinol^TM^, used in three clinical trials for asthma [207,209,210] (Table 7 and Table 8). Notwithstanding, there is significant work dedicated to the general anti-inflammatory activity of molluscan compounds(recently reviewed by Ahmad et al. [49]).

Molluscan compounds displaying potent anti-inflammatory activity in vitro, but not appearing in studies specific to respiratory disease (and therefore not included in Table 5, Table 6 and Table 7), include: abalone (*Haliotis* sp.) body [216,217] and shell [218] extracts; lipid extracts of the freshwater snail *Filopaludina bengalensis* [219], the Humboldt squid [220], Australian cephalopods [151], and Aplysiidae sp. [221]; and isolated compounds derived from Muricidae sp. [222,223,224,225,226,227] and four Asian marine bivalves (*Magallana bilineata* [originally *Crassostrea madrasensis*, Preston, 1916] [228,229], *Perna viridis* [230], *Anadara kagoshimensis* [originally *Arca subcrenata*, Lischke, 1869] [231], and *Villorita cyprinoides* [232]). Additionally, molluscan compounds displaying potent anti-inflammatory activity in vivo, but not appearing in studies specific to respiratory disease, include: different extracts (foot lipid, extra-pallial fluid, flesh homogenate and purified proteins) of Indian freshwater snail *F. bengalensis* [219,233,234,235]; various extracts of Indian marine gastropods [173,184,236,237,238,239,240] and Sepiidae sp. [241,242]; lipid extracts of three mussel species [243,244,245]; a preparation of the pearl of *Pinctada imbricata* [242]; and melanoprotein from the neon flying squid *Ommastrephes bartramii* [246]. These and other molluscan extracts and compounds clearly need to be studied for respiratory-associated anti-inflammatory activity given the significance of the pathway in respiratory disease, differences in presentation from other sites of inflammation, and the need for alternatives (e.g., COX-2 specific inhibitors) to available anti-inflammatory medications.

### 4.4. Anticancer Activity

The A549 (human alveolar carcinoma) lung cancer cell line is often used to investigate anticancer activity in vitro, enabling comparison between studies (Table 6). Significant inhibition of A549 proliferation has been shown using a recombinant tumor necrosis factor from the Pacific oyster (200 ng/mL) [247], crude methanol extracts of an intertidal snail *Euchelus asper* (40% inhibition at 10 µg/mL) [248], and lipid extract from a squid digestive gland (CC_50_ 260 µg/mL) [249] (Table 6). Liu et al. [250] tested extracts of Ampullarriidae, a family of freshwater snails used in TCM for epilepsy and stomach ache, reporting 31-57% inhibition of A549 between 20–200 µg/mL, and Zhang et al. [251] reports 73-96% inhibition using ethanol extracts of eight Chinese mollusc species, some of which are used in TCM [104]. However, these studies are less robust than others; only three studies included in Table 6 report cytotoxicity against human/animal-derived healthy cell lines, which is critical to ensure specificity [249,252,253] (Table 6).

In assays using a novel peptide (termed Mere15) from the Asiatic clam *Meretrix meretrix* (Veneridae), Wang et al. [252] found that A549 was more susceptible (IC_50_ 31.80 µg/mL) than other cancer (breast, colorectal, liver, pancreas; IC_50_ 43.5–57.4 µg/mL) and healthy (IC_50_ 123.1–149.5 µg/mL) cell lines (Table 6). These results prompted the use of A549 in a mouse xenograft model, whereby 50 mg/kg Mere15 administered by subcutaneous injection inhibited tumor growth by 69%, an effect greater than (although statistically indifferent to) the control chemotherapeutic cyclophosphamide (53% inhibition at the same concentration) [252] (Table 7). The Veneridae are valued in TCM with 42 different species used in 11 remedies for inflammatory respiratory diseases (e.g., asthma, cough, tuberculosis, tracheitis), but not cancer [104] (Table S2); Veneridae sp. are also used as medicines in South America for asthma and influenza [113] (Table S1). Anti-inflammatory activity of extracts and compounds from this family is a topic certainly worth pursuing, in conjunction with anticancer work.

**Figure 5:**
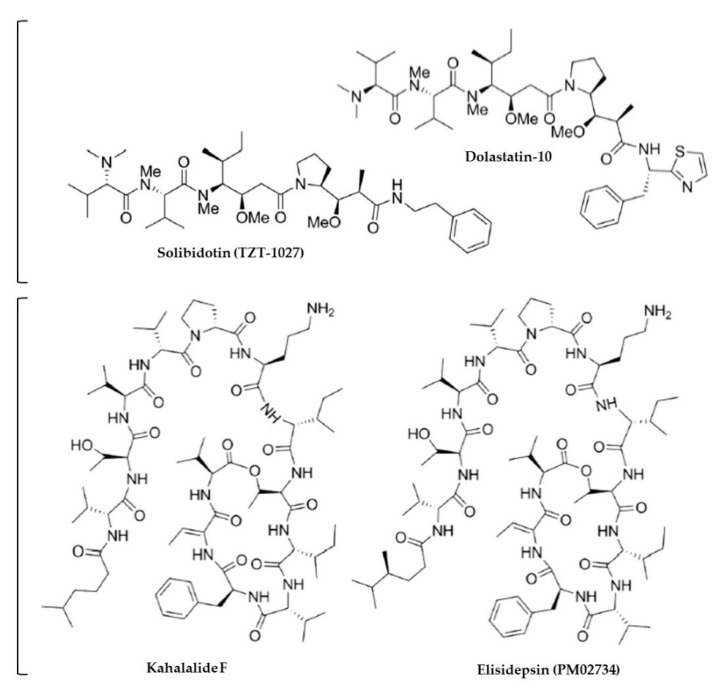
Dolastatin-10, kahalalide F, and derivative compounds of molluscan origin showing potent activity against respiratory cancers. Adapted from [67].

The best available evidence for molluscan anticancer compounds to date comes from kahalalide F (KF) (a cyclic depsipeptide), dolastatin-10 (a linear pentamer with four unique amino acids), and structurally related analogues, which have progressed from in vitro through in vivo studies into Phase I and II clinical trials for respiratory, and other, cancers (Table 6, Table 7 and Table 8, Figure 5). The strong activity of dolastatin 10 is illustrated by Kalemkerian et al. [254], who report low IC_50_s (0.03–0.184 nM) against a panel of lung cancer cell lines (Table 6) and found that two doses of 4.5 mg/kg dolastatin 10 could completely inhibited tumor formation, or significantly reduce the size of established tumors (1635 mg control vs. 44 mg dolastatin-10) and improve survival in a H446 (small cell lung cancer) xenograft model (Table 7). Once a lead compound is identified, a research team may synthetically explore and alter the structure in order to maintain its favourable properties while improving on deficiencies, as did Kobayashi et al. [255] who found that TZT-1027, a synthetically modified dolastatin-10 derivative designed for enhanced bioactivity and lower toxicity, resulted in 84–98% regression of pre-established lung cancer xenograft tumors with just one or two treatments at 1–2 mg/kg (Table 7, Figure 5).

GI_50_/IC_50_ values for KF against A549 range from 0.135–1 µM (0.20–1.48 µg/mL) [253,256,257], which are far lower than cytotoxic concentrations (>4.76 µg/mL [253]) (Table 6). A549 appears more sensitive to KF than other cancer types in vitro (e.g., colon, leukemia, melanoma, ovarian, prostate; [253,257]), although other respiratory cell lines are more resistant (e.g., H460 [256], HOP62 [258]). Three clinical trials involving KF and elisidepsin (a KF derivative; Figure 5) have shown their capacity to stabilize lung cancer (by induction of apoptosis, inhibition of microtubule formation and blockade of cellular growth pathways, as opposed to immunomodulatory mechanisms) at maximum tolerable doses of around 6.5 mg/m^2^ [259,260,261,262] (Table 8). Interestingly, elisidepsin has been shown to act synergistically with paclitaxel, cisplatin, and gemcitabine against lung cancer cell lines in vitro [258] and with Erlotinib in vivo, significantly enhancing the survival of mice with A549 tumors (elisidepsin 54 d, erlotinib 39 d, combination >150 d, control 23 d) [263] (Table 6, Table 7 and Table 8). Such combinations of molluscan compounds with approved drugs may help to overcome chemotherapy resistance. Myelosuppression and elevated transaminase levels are dose-limiting drawbacks of KF and dolastatin compounds in vivo, as per many chemotherapeutic agents [254,260,264,265].

Original sources of KF and dolastatin-10, the tropical ornate leaf sea slug, *Elisia ornata* (Plakobranchidae) and the sea hare, *Dolabella auricularia* (Aplysiidae) respectively, are rarely used in traditional medicine, with the exception of an unknown Aplysiidae sp. body part used in Middle Eastern medicine (for cough, dyspnea, and hemoptysis) and egg masses used in one TCM (for cough, TB and dyspnea) (Tables S1 and S2). Aplysiidae sp. egg masses contain a range of bioactive compounds, but not dolastatins, [266] and there is evidence for acute liver damage upon ingestion [267]. The bias of natural product cancer research toward heterobranch gastropods, in which the shell is often reduced or entirely absent, is not justified by the potential for toxic (anti-predatory) compounds and the fact that traditional molluscan medicines are largely derived from shelled species (Tables S1 and S2, Figure 1) [53].

The lung is the preferred site for metastases of common cancers originating elsewhere (e.g., breast, colon, prostate and bladder) in up to 52% of cases [268] and such metastases are the single most negative factor in the cancer prognosis [269,270]. Gesheva et al. [76] measured lung metastases in a murine model of colon cancer, whereby direct solid C-26 (colon) tumor injection with Hcs from *R. venosa* (RvH; Figure 3) or *Helix pomatia* (HpH) resulted in significant reductions in tumor size, high anti-C-26 antibody levels, marked reductions in surface lung metastases and up to 30% increased survival after >13 weeks (Table 7). The effectiveness of RvH was dependent on priming before tumor inoculation, yet HpH was effective even without it [76], suggesting that the mechanism of action is related to modulation of cellular immunity (i.e., natural killer cells, antigen presenting cells, and specific CD8^+^ and CD4^+^ T cells) by Hc and subsequently, better recognition and elimination of abnormal cells [271,272].

Synergeneic (or allograft) models involve the transplant of immunologically compatible cancer cells into immunocompetent mice [273]. Because of these qualities, these models are useful to study interactions between tumors, functional host immune systems and treatments, and eliminate the potential for tumor rejection that exists in xenograft models unless mice are first immune compromised. The only reproducible syngeneic model for lung cancer to date is the Lewis lung carcinoma model [273], as used by Gomes et al. [274], who measured the formation of lung metastases in mice treated with a heparin-like glycan from *Nodipecten nodusus*, a bivalve mollusc found along the north Atlantic coast of America. In this study, the size and number of metastatic lung foci were significantly reduced, from ten to one per lung (PBS control and molluscan heparin treatment groups, respectively) [274] (Table 7). The in vivo and in vitro components of the study by Gomes et al. [274] demonstrated that molluscan heparin inhibits P-selectin interaction with colon carcinoma (LS180) and reduces inflammatory cell recruitment, platelet-tumor cell complex formation, and heparinase enzymatic activity, thereby attenuating metastases, although not to an extent beyond its mammalian counterpart. Heparin analogues from other mollusc species that possess strong anticoagulant activity [275,276,277,278] are yet to be tested as chemotherapeutics.

Abundant literature has been published on the anticancer activity of brominated indole and isatin derivatives from muricid molluscs in vitro [75,147,251,279,280,281,282,283,284,285] and in in vivo models of cancer treatment [285,286] and prevention [48,214,287,288] (Figure 4). Zhang et al. [251] present the only evidence for Muricidae sp. extracts against respiratory cancer, indicating that they can inhibit A549 proliferation and regulate adaptive immunity in vitro, but further analysis is required to determine significance and identify which, if any, extracts are productive to pursue. The recent review by Ciavatta et al. [66] covers other potentially promising mollusc-derived anticancer agents, including terpenes, steroids, peptides, polyketides, and nitrogenous compounds, currently untested against respiratory cancers.

Depending on the specific mode of action, certain compounds can exhibit multiple related activities with multiple potential therapeutic benefits in the context of respiratory disease. For example, 6-bromoisatin has anticancer [77,212,279,289] as well as anti-inflammatory [84,213] and antibacterial activity [149]; helicidine has both anti-spasmodic and anti-inflammatory properties [79], while KF possesses antimicrobial as well as anticancer activity [253] (Table 5, Table 6, Table 7 and Table 8, Figure 4 and Figure 5). This highlights the importance of comprehensive in vitro screening and structure-target identification, and suggests that the full range of activities are yet to be elucidated for many compounds included in this review.

**Table 5:** Antimicrobial (antiviral, antibacterial, antifungal) activity of molluscan extracts and compounds tested in vitro.

Mollusc Class Family	Derivative Part	Specific Extract/ Compound	Microbial or Cellular Target	Effective Concentrations *	Other Important Findings	Ref
**Bivalvia**						
Mytilidae	Sperm	Crude perchloric acid extract (CE) and 3 isolated protamine-like (PL) proteins	Clinical and lab strains: *Staphylococcus aureus*, *Pseudomonas aeruginosa*, *Klebsiella pneumoniae*; human lymphocytes and red blood cells	MICs 7.8–250 μg/mL; MBCs (μg/mL): CE: 15.7–125, PL-II 15.7–125, PL-III 62.5–250, PIV 62.5–250	Digested and non-digested PL-proteins had same effect; low toxicity to lymphocytes (80–90% viability), no sig. hemolysis; effect re protein membrane binding, cytosolic intrusion and nucleotide leakage	[187]
	Hemolymph (hemocytes)	Myticin C and 9 peptide fragments	*P. aeruginosa, S. aureus, Micrococcus lysodeikticus*	MICs >64 μM for *P. aeruginosa*; MIC 32 μM of 3 peptide fragments for *S. aureus*		[168]
	Hemolymph (hemocytes, plasma)	Myticin A and B peptides	*M. luteus, Bacillus megaterium, S. aureus* (clinical strain)*, Listeria monocytogenes* (G+); *P. aeruginosa* (clinical strain)*, Brucella suis* (G−); Fungi: *Fusarium oxysporum*	MBCs (μM): G+ 2.25- >20 Myt A, 1- >20 Myt B; G− >20 Myt A and B; fungi >20 Myt A, 5–10 Myt B		[167]
Ostreidae	Hemolymph	Cellular (c) and acellular (a) hemolymph fractions (0.2 μm filtration)	Human adenovirus (respiratory strain AdV-5) cultured in Vero and HEp-2 cell lines	CC_50_ 0.19–0.36 mg/mL; EC_50_ 0.05–0.16 mg/mL	*Crassostrea rhizophorae* cellular fraction showed best (64%) viral inhibition, particularly w post-infection treatment; virus preincubation w both fractions protected >90% of cells indicating virucidal activity at non-cytotoxic concs	[196]
Teredinidae	Gill (symbiotic *Teredinibacter turnerae*)	Tartrolon E	*P. aeruginosa*, methicillin-sensitive and methicillin resistant *S. aureus* (MSSa, MRSa)	MICs (mg/mL): 0.31 for *P. aeruginosa*, 0.08 for MSSa, 1.25 for MRSa		[169]
**Cephalopoda**						
Octopodidae	Suckers	Peptide (OctoPartenopin) (crude + 6 HPLC fractions + 5 synthetized fractions)	*S. aureus, P. aeruginosa*	MIC80 (μg/mL) 50–200 *S. aureus*, 50- >300 *P. aeruginosa*; 80 μM peptides inhibit and eradicate up to 60% biofilm formation	Also antifungal activity; improved activity with synthetized peptides	[170]
Sepiidae †	Shell	Chitosan	Bacteria: *K. pneumoniae, Bacillus cereus* (G+), *S. aureus, M. luteus* (G−); Fungi: *Aspergillus niger, Fusarium* sp.	50 mg/mL (preliminary)	*S. officinalis* chitosan stronger activity than shrimp and crab chitosan; ZIs similar to gentamycine for G−, > cyclohemimide for fungi	[135]
	Shell	Chitosan	*Streptococcus pneumoniae, S. aureus* (G+)*, P. aeruginosa, K. pneumoniae* (G−)	MIC (μg/mL): 60–100 (G−), 100 (G+)	Higher MICs for phosphorylated chitosan	[136]
	Salivary glands	PSG toxin (glycopeptide)	*K. pnemoniae, Streptococcus pyogenes*	1–50 μM (preliminary)	Little difference between 1–50 μM concs; low toxicity to zebrafish embryo	[290]
		PSG toxin (glycopeptide)	*K. pnemoniae, S. aureus, P. aeruginosa*	25–100% (preliminary)	Susceptibility: *S. aureus* > *K. pneumonia > P. aeruginosa*; ZIs comparable to Ciprofloxacin	[291]
Sepiidae, Octopodidae	Body	Crude CH_3_OH extracts	Clinical bacterial strains: *P. aeruginosa, K. pnemoniae, S. aureus, S. pneumoniae, Streptococcus* sp., *Vibrio alginolyticus*; Fungi: *Pencillium italicum, Alternaria alternata* (allergen), *Fusarium equisetii*	MIC range 60–100 mg/mL	Extract from *S. kobiensis* showed the best/broadest spectrum activity; no positive control or toxicity data	[185]
**Gastropoda**						
Achatinidae	Mucus	Mytimycin-AF (antimicrobial peptide)	*S. aureus, B. megaterium, K. pneumoniae*	MIC (μg/mL) *S. aureus* 1.9, *B. megaterium* 15*, K. pneumoniae* 30	Better activity than human AMP control for *S. aureus*; minimal hemolysis (max 3.9% at 329 μg/mL)	[292]
Achatinidae, Helicidae	Mucus	Crude mucus and 4 size-separated fractions	*K. pneumoniae, P. aeruginosa* (3 strains), *S. aureus, S. pyogenes, Acinetobacter sp.* (clinical), *Serratia marcescens* (clinical)	1:3 crude mucus:PBS (preliminary)	Crude *H. aspera* mucus inhibited *S. aureus* and *P. aeruginosa*; *A. fulica* mucus inhibited *S. aureus*; other microorganisms unsusceptible	[174]
Babyloniidae	Body	Crude extracts ‡	*P. aeruginosa, K. pneumoniae, S. aureus, S. pneumoniae*; Fungi: *A. flavus*	Crude extract (preliminary)	Ethanol extract had highest antimicrobial activity; most effective against *P. aeruginosa*, least effective against *S. aureus*	[175]
Clathurellidae	Hepatopancreas (symbiotic *Streptomyces* sp.)	CH_3_OH extract (lobophorin compounds)	*Mycobacterium tuberculosis, P. aeruginosa, Burkholderia cepacia;* CEM-TART cell line	MIC_90_: 1.3–24 μM for *M. tuberculosis*, >100 for *P. aeruginosa* and *B. cepacia*	Strong cytotoxicity at similar MIC concentrations (0.3–100 μM) therefore not a suitable therapeutic candidate	[117]
Conidae	Venom	Conotoxin MVIIA and 9 analogues	*S. aureus*	MICs: >500 μM MVIIA, 7–78 μM analogues	MVIIA considered inactive, different activity among analogues re. cyclic structure and side chain modification	[293]
Cypraeidae ^‖^	Shell	Powder	*Micrococcus* sp.	4–5% w/v shell powder in distilled water (preliminary)	Dose-dependent antipyretic effect in vivo (not sig)	[134]
Dorididae	Sperm (also in egg masses)	5’-deoxy-5’-methylthio-adenosine (MTA) and two natural analogues (xylo-MTA and xylo-A)	*S. aureus, Corynebacterium diphtheriae;* Vero and C8166 cell line	MICs: MTA 33 μM, xylo-MTA 200 μM, xylo-A 18 μM	MICs always higher than minimum non-toxic concentrations; xylo-A most toxic, xylo-MTA least toxic; no positive control	[172]
Fissurellidae	Body	Scutinin A and B	*P. aeruginosa*	MIC: 30 μg/mL scutinin A, 100 μg/mL scutinin B		[171]
Helicidae, Muricidae	Hemolymph	Experimentally purified Hc (βc-HaH subunit + 8 FUs, RvH1 + 4 FUs)	*S. aureus, S. pyogenes, P. aeruginosa*	MIC: 6.5 μM βc-HaH; MIC not calculated for RvH1 (1.25–10 μM range)	HaH more effective than RvH; native Hc more effective than subunits from both species; βc-HaH *S. aureus* and *S. pyogenes* 60 and 51% inhibition respectively, RvH1 35% inhibition, relative to control; limited activity against *P. aeruginosa*	[73]
Muricidae	Hemolymph	Experimentally purified Hc (RvH), glycosylated (RvH-c) and non-glycosylated (RvH-b) subunits	Respiratory synctial virus (RSV), cultured in Hep-2 cell line	RvH-c 1 mg/mL	RvH-c effective against replication of RSV (71.4% inhibition at 1 mg/mL), no effect on other tested viruses (poliovirus, cocksackie virus); native RvH and RvH-b no antiviral activity; no cytotoxic effect at highest concs	[197]
	Egg masses	Crude extracts (de, eth, CHCl_3_, CH_3_OH-H_2_O) ^#^ and isolated ty, tv, Tp, 6-b ^§^	*P. aeruginosa* (G−), *S.aureus* (G+)	MICs (mg/mL): 0.0005 tv, 0.5–1.0 ty, 0.1–1.0 6-b, >1 Tp, 1.0–10 CHCl_3_, 0.1 de, 10 eth, >50 CH_3_OH-H_2_O	Lipophilic extracts had better activity; tv bacteriostatic, ty bactericidal	[149]
	Hemolymph	Experimentally purified Hc (11 protein fractions)	*S. aureus, K. pneumoniae*	113–598 μg/mL	Peptides 8, 9, 10 and 11 showed >90% inhibition; *S. aureus* more susceptible; different proteins more/less active against different bacteria; longer protein chains more effective	[294]
	Body	Crude extracts **	*K. pneumoniae, P. aeruginosa, S. pneumoniae, Citrobacter sp., B. cereus*	MICs 0.05–0.12 mg	Acetone extract most effective; similar effectiveness against other (non-resp) pathogens	[184]
Olividae	Body	Acid-acetone peptide extract	*S. aureus, P. aeruginosa, K. pneumoniae*	MIC (mg/mL): 2.5 *S. aureus*, 0.039 *P. aeruginosa*, 1.25 *K. pneumoniae*; MBC (mg/mL): 2.5 *S. aureus*, 1.25 *P. aeruginosa*, >2.5 *K. pneumoniae*	Protein ZIs comparable to control antibiotics; ciprofloxacin and cefotaxime MICs reduced by >100% w protein extract, metronidazole and erythromycin MICs increased; effects re changes in membrane porosity/permeability	[179]
	Body	Acid-acetone peptide extract	*P. aeruginosa*	MIC: 39.06 ug/mL (Gentamycin MIC 1.95 ug/mL)	Bacteriostatic; dose dependent reduction in virulence factors (pyoverdine, pyocyanin, protease)- peptide mix (69%) similar to gentamycin (72%) at 1/2 MIC; 50% reduction in biofilm formation at 39 ug/mL, 2.5 mg/mL required to degrade pre-formed biofilm	[178]
Onchidiidae	Body	Dolabellanin B2 (AMP)	*S. aureus, P. aeruginosa, K. pneumoniae*	MICs: 10–25 ug/mL	Structure-function characterization; better activity against G+; compound identified as one previously isolated from *Dolabella auricularia*	[295]
Patellidae, Donacidae ††	Body	Acid-acetone extract	*S. aureus, S. pneumoniae, K. pneumoniae, P. aeruginosa*	MICs 17–20 mg/mL	ZI’s similar to ciprofloxacin; *Galeta paradoxa* extract stronger antibacterial than *Patella rustica* extract, which showed good antifungal activity	[296]
Pharidae	Body ‡‡	2 sialic acid-binding lectin recombinant proteins (rSgSABL-1, -2)	*Staphylococcus aureus, Micrococcus luteus*; *Solen grandis* (mollusc) hemocytes; mice (n = NA) immunised i.p. 2x w rSgSABL-1 (100 μg/mL) or rSgSABL-2 (180 μg/mL) in CFA ^‖‖^	100 μg/mL (phagocytosis), 90 μg/mL (microbe agglutination, and encapsulation); 100–180 μg/mL (Ab production)	High binding affinity to *S. aureus* peptidoglycan (PAMP) (also LPS, β-glucan); agglutination effect on *M. luteus*; enhanced phagocytosis and encapsulation ability (*p <* 0.05); antisera Ab reactivity with rSgSABL-1 and -2	[188]
Plakobranchidae	Body	Kahalalide F and 8 analogues	Bacteria: *P. aeruginosa,* methicillin resistant *S. aureus, M. tuberculosis* (H37Rv), *M. intracellulare*; Fungi: *Cryptococcus neoformans, A. fumigatus, Fusarium* sp.	MIC 9.4–>16 μg/mL Kahalalide F analogues (*M. tuberculosis*); 30 μM antifungal	>90% inhibition of *M. tuberculosis* and up to 100% fungicidal activity comparable to controls; no activity against *P. aeruginosa* or *S. aureus*; high test concs- cytotoxicity test concs lower than MICs	[253]
	Body	Kahalalide F, analogues KZ1 and KZ2	*Aspergillus* sp., *Fusarium* sp.	20 mg KZ1 and KZ2	Analogue bioactivity profiles comparable to KF; antifungal activity comparable to ketonazole	[257]
Strombidae	Body	Crude extracts ^##^	*S. aureus, P. aeruginosa*	1–100 μg/mL H_2_O extract (preliminary)	Stronger activity against *P. aeruginosa* over *S. aureus*	[297]
Truncatellidae	Body (symbiotic *Streptomyces* sp.)	7,8-dideoxygriseorhodin C (DC)	Methicillin-resistant *S. aureus;* MDCK and AA8 cell lines	MICs (μg/mL): 0.08–0.12 DC, 1.59–6.24 oxacillin; combination DC 0.01–0.02 DC and 0.02–0.298 Oxacillin	DC stronger than oxacillin as single agents; reduction MICs w combination; no cytotoxicity (IC_50_ 15.84 μg/mL and >49.5 μg/mL for MDCK and AA8 cells, respectively)	[176]
Veronicellidae	Mucus	Concentrated crude mucus and 4 fractions (PUFA 39, 40, 49, 50)	Measles virus (Edmonston wild-type), cultured in Vero cell line	60–220 ng/mL mucus/fraction 39 inhibition of viral replication; 2% mucus/fraction 39 inhibition of CPE	Effect attributed to disruption of the virus’ lipoprotein envelope; not cytotoxic to Vero cells (IC_50_ 41 μL crude, 92.6 μL fraction 39)	[154]
	Mucus	Crude concentrated mucus and 3 fractions (PUFAs 39, 40, 49)	Influenza A (H1N1) virus, cultured in MDCK cell line	2% or 60–80 ng/mL crude mucus and fraction 39	Inhibition of viral replication and >80% decrease in viral load in infected cells w crude mucus and frac 39; not cytotoxic (although IC_50_ NA); may interfere w binding of virus to host cell receptor	[153]
23 families ^§§^	Egg masses	Crude homogenised egg material and extracts †††	*P. aeruginosa, S. aureus*	1–10 mg/mL CHCl_3_ and CH_3_OH-H_2_O extracts (preliminary)	*S. aureus* and *P. aeruginosa* inhibited by 79% and 72% of tested egg masses, respectively; no dif in activity between tough and gelatinous egg masses	[65]
16 families ***	Body	CHCl_3_ extracts	Bacteria: *S. aureus, P. aeruginosa, K. pneumoniae;* Fungi: *A. fumigatus*; chicken red blood cells and brine shrimp	Crude extracts (preliminary)	Positive antimicrobial activity; best result w extract of *Conus betulinus*; low toxicity to brine shrimp (LC_50_ 12–42 μg/mL); 10/25 sp. extracts showed hemolytic activity; no antibiotic controls	[298]
5 families ‡‡‡	Body, gill and mantle (GM), digestive gland (DG)	10, 40 and 80% SPE fractions of acidic (HCl) extract in sterile water	*M. luteus* and *B. megaterium*; Vero cell line	Most effective MICs (μg/mL): 43 80% DG extract both bacteria, 63 80% DG extract *M. luteus*, 40 80% G+M extract *B. megaterium,* 2560 *M. luteus*)	40 and 80% fractions from all sp. effective; *Crassostrea edule* extracts showed best activity (also against non resp viruses); <50% cytotoxicity; positive controls (lysozyme and polymyxine B) more effective than extracts	[45]

Abbreviations: Hc: hemocyanin; w: with; TB: tuberculosis; EC_50_: 50% effective concentration; IC_50_: concentration causing 50% growth inhibition; MIC: minimum inhibitory concentration; MBC: minimum bactericidal concentration; PUFA: polyunsaturated fatty acid; CPE: cytopathic effects; ZI: zone of inhibition; PBS: phosphate buffer solution; NA: not available; AMP: antimicrobial peptide. * Description of methods and test concentrations provided in supplementary tables; preliminary data derived from agar disc diffusion methods reporting ZIs. † *Sepiella inermis* originally listed as *Sepia inermis* (Ferussac & d’Orbigny 1835); *Sepioteuthis lessoniana* originally listed as *Sepia lessoniana* (d’Orbigny, 1826); *Amphioctopus aegina* originally listed as *Octopus aegina* (Gray 1849) and *O. dollfusi* (Robson 1928); *A. fangsiao.* originally listed as *O. areolatus* (in Ferussac & d’Orbigny 1839–1841). ‡ Methanol, ethanol, chloroform, and acetone solvents. ^‖^ *Monetaria moneta* originally listed as *Cypraea moneta* (Linnaeus 1758). ^#^ Extracts using chloroform (CHCl_3_), methanol-water (CH_3_OH-H_2_O), diethyl ether (de) and ethanol (eth) solvents. ^§^ Tyrindoleninone (ty), tyriverdin (tv), Tyrian purple (Tp) and 6-bromoisatin (6-b). ** Ethyl acetate, acetone, dichloromethane and methanol solvents and cold-steeped; *Drupella margariticola* originally listed as *Drupa margariticola* (Broderip, 1833). †† Bivalvia. ‡‡ Described as body extract in text although lectins are commonly found in hemolymph. ^‖‖^ Antisera used in Western Blot analysis; rSgSABL-1 and -2 and antisera used in PAMP assay; rSgSABL-1 and -2 used in phagocytosis, agglutination and encapsulation assays. ^##^ Chloroform, methanol, hexane, acetone and water solvents. ^§§^ Major represented families include Muricidae (7 sp.), Aplysiidae (6 sp.), Amphibolidae (2 sp.) Planorbidae (2 sp.), Pleurobranchidae (2 sp.) and Doridae (2 sp.) representing classes Bivalvia and Gastropoda. ††† Chloroform and methanol/water extracts. *** Major represented families include Strombidae (4 sp.), Conidae (3 sp.), Octopodidae (2 sp.), Sepiidae (2 sp.), Veneridae (2 sp.) and Aplysiidae (2p.) representing classes Bivalvia and Gastropoda. ‡‡‡ Cardiidae, Veneridae, Ostreidae, Calyptraeidae and Buccinidae representing classes Bivalvia and Gastropoda.

**Table 6:** Molluscan extracts and compounds showing anticancer and immune modulatory activity in vitro.

Mollusc Class Family	Derivative Part	Specific Extract/ Compound	Microbial or Cellular Target	Effective Concentrations	Other Important Findings	Ref
**Bivalvia**						
Mactridae *	Body	Spisulosine	SW1573 (human alveolar carcinoma) cell line (and 4 other human tumor cell lines)	GI_50_: 1.3 μM	Spisulosine most effective of tested compounds- >positive controls Cisplatin (3.0 μM) and Etoposide (15.0 μM); GI_50′_s 0.7–2.6 μM for range of cell lines- SW1573 intermediate sensitivity; selective CK1ε inhibition	[299]
Ostreidae	Hemolymph (hemocytes)	Tumor necrosis factor (*Cg*TNF-2)	A549 (human alveolar carcinoma)	200 ng/mL recombinant *Cg*TNF-2	*Cg*TNF-2 expression upregulated in response to bacterial PAMPs (incl. *Staphylococcus aureus*), and serum lysosome activity, NO content and antibacterial activity (non-resp) increased (*p <* 0.05)	[247]
Veneridae	Body	(NH_4_)_2_SO_4_ fractionated peptide (‘Mere15′)	A549 and range of other non-respiratory cancer cell lines (breast, cervical, colorectal, pancreatic, liver); benign cells NIH 3T3 and MCF-10A	IC_50_: 31.8 μg/mL	A549 most susceptible among cancer types, therefore used in subsequent assays and animal model; not cytotoxic to benign cells (IC_50_ > 120 μg/mL)	[252]
**Cephalopoda**						
Loliginidae †	Digestive gland/liver	Lipid extract	A549 and Vero cell lines	70% growth inhibition at 960 μL/mL, 55% at 480 μL/mL; CC_50_ 260 μg/mL for A549 (NA for Vero)	Better growth inhibition of A549 (max 70%) compared to Vero (max 7%)	[249]
Gastropoda						
Ampullariidae	Body	“Polysaccharide extract” ‡	A549 cell line	20–200 μg/mL	24-h reduction in tumor growth: 31% at 20 mg/mL, 43% at 50 mg/mL, 46%, at 100mg/mL, 57% at 200 μg/mL; 84% antioxidant at 5 mg/mL	[250]
Aplysiidae	Body	Dolastatin-10	Human SCLC cell lines (NCI-H69, NCI-H82, NCI-H446, NCI-H510)	IC_50_ range 0.03–0.184 nM	>50% G_2_/M phase arrest, bcl-2 phosphorylation; pro-apoptotic mechanism	[254]
Chilondontidae	Body	Crude CHCl_3_ extract (1.25%) in Hanks Balanced Solution	A549 cell line	5–20 μg/mL	30–40% cytotoxicity at 5–20 μg/mL; apoptosis at 10 μg/mL, not increasing w higher doses or exposure time; wound area reduced by 28.3% at 5 μg/mL; all results *p <* 0.05; inhibitory effect on matrix metalloproteinase	[248]
Helicidae	Mucus	Helicidine formula (glycoproteins) (purified NaCl extract)	Tracheas dissected from Dunkin-Hartley guinea pigs; epithelium (E+) and epithelium-free (E−) strips prepared	0.005–0.5 mg/mL (min-max effective)	Dose dependent reduction of contraction by 35% in E+ and 25% in E−; PGE_2_ higher post treatment (*p <* 0.01); related to COX inhibition (*p <* 0.01)	[79]
	Hemolymph	Experimentally purified Hc (HpH)	Influenza (H3N2) immunisation model using Balb/c mice (n = 5–8/group) immunised w 50 μg influenza peptide (IP), IP w CFA, IP w alum, or IP w HpH (16, 40, 100 μg)	100 μg HpH; 50 μg antigen + 100 μg HpH	Ex vivo spleenocytes of mice treated w IP+100 μg HpH showed stronger cytotoxicity against infected cells in vitro compared to all other groups (*p <* 0.0005)	[83]
Plakobranchid-ae	Body	Kahalalide F (KF) and 8 analogues	A549 and NCI-H322M (human bronchioalveolar carcinoma) and Vero cell lines	GI_50_: 0.131–13.7 μM (compound-tumor specific e.g., for A549 analogues 8 and 16 IC_50_ 0.166 and 13.189 μM, 0.165 and 0.167 µM for NCI-H322M)	Some compounds showed higher potency than Paclitaxel; similar anticancer activity among other tested cancer cell lines; no cytotoxicity at 4.76 μg/mL	[253]
	Body (originally)	KF (synthetic)	4x human NSCLC cell lines (A549, SW1573, NCI-H292 and NCI-H460)	IC_50_ 0.1–7.0 μM	A549 and H292 particularly sensitive, H460 least sensitive; inhibition of ErbB andPI3K-Akt signaling at IC_50_ concs and necrosis-like cell death	[256]
	Body (originally)	PM02734 (elisidepsin trifluoroacetate; synthetic KF3 derivative)	HOP62 (human lung adenocarcinoma), A549 (human alveolar carcinoma), DV90 (human metastatic pleural carcinoma) cell lines	Elisidepsin IC_50_ ~4 μM for HOP62, <0.25 μM for A549, ~0.3 μM for DV90	Downregulation of ErbB3, Akt and MAPK pathways in all cell lines; synergistic/additive effects w other cisplatin, paclitaxel and gemcitabine in all cell lines- combination therapy to improve clinical efficiency	[258]
	Body (originally)	PM02734 (elisidepsin trifluoroacetate; synthetic KF3 derivative)	8 x human NSCLC cell lines (H322, A549, H661, H1299, H1975, H358, H460, H1650)	IC_50_ 0.3 μM to >5 μM (0.58 μM for A549)	All cell lines sensitive to PM02734, only 2 cell lines sensitive to erlotinib; positive correlation between ErbB expression and sensitivity to PM02734; erlotinib inhibited EGFR, AKT and ERK1/2 phosphorylation whereas PM02734 strongly inhibited phosphorylation of ErbB3 and AKT and, to a lower extent, EGFR and ERK1/2 hence the efficacy of combined treatment in vivo	[263]
	Mucus (and body)	KF, analogues KZ1 and KZ2, crude CHCl_3_-CH_3_OH extract	A549 (and other non-respiratory cancer) cell lines	A549 IC_50_: 1 μM KZ1, 3 μM KZ2, 1 μM	Analogue bioactivity comparable to KF; low IC_50_ values for lung cancer relative to other tested cancer cell lines; mucus extracts stronger	[257]
7 families ^‖^	Body	95% C_2_H_5_OH extract	A549 cell line; mouse spleenocytes	0.25–1 mg/mL	73–96% tumor growth inhibition at 1 mg/mL, 63–89% at 0.25 mg/mL; molluscan extracts showed stronger anti-tumor properties than other invertebrate extracts and had strong promotion activity on T and B lymphocytes (+25% at 1 μg/mL); low toxicity (not quantified)	[251]

Abbreviations: w: with; NA: not available; CC_50_: 50% cytotoxic concentration; EC_50_: 50% effective concentration; IC_50_/GI_50_: concentration causing 50% growth inhibition; CFA: Complete Freund’s Adjuvant; SCLC: small-cell lung cancer; NSCLC: non-small-cell lung cancer; PAMPs: pathogen associated microbial patterns. * *Mactromeris polynyma* originally listed as *Spisula polynyma* (Stimpson, 1860). † *Uroteuthis* (*Photololigo*) *duvaucelii* originally listed as *Loligo duvaucelii* (in Ferussac & d’Orbigny 1835). ‡ Methanol (CH_3_OH), ethanol (C_2_H_5_OH), acetone (C_3_H_6_O) and ether solvents. ^‖^ Acanthochitonidae, Arcidae, Veneridae, Muricidae (2 sp.), Nacellidae, Naticidae representing classes Bivalvia, Gastropoda and Polyplacophora; *Glossaulax didyma ampla* originally listed as *Neverita ampla* (Phillipi 1849), *Reishia clavigera* originally listed as *Thais clavigera* (Kuster 1860).

**Table 7:** In vivo animal models of respiratory disease using molluscan extracts and compounds showing various bioactivities.

Mollusc Class Family	Derivative Part	Specific Extract/ Compound	Model Design *	Main Findings	Effective Concentrations	Ref
**Bivalvia**						
Mytilidae	Body	Lipid extract (‘Lyprinol’)	Murine model of allergic airway disease using Balb/c mice (n = 3–8/group) fed a low-fat background diet treated w 200 uL Lyprinol (or fish oil control) p.o daily, 14 d prior to challenge w i.n. OVA (10 mg in 0.9% saline) (or PBS alone) on days 12–15	Lyprinol group had lower eosinophil counts and fewer mucus-secreting cells (*p <* 0.05), other inflammatory cells lower (not sig); no sig dif. in Ab levels between fish oil and Lyprinol; lower IL-13, higher IL-4 and IFN-y in Lyprinol group (*p <* 0.05); both fish oil and Lyprinol suppressed airway resistance (*p <* 0.05); Lyprinol efficacy suggested re. synergistic effects between multiple nutritional components/PUFA profile	200 μL	[208]
Pectinidae	Body	Protease extracted polysaccaride heparin sulfate analog (HS)	Lung metastasis model using mice (n = 9) treated w 8 mg/kg HS i.v. (or mammalian heparin or chondroitin controls) 10 min before challenge w i.v. Lewis lung carcinoma cells; separate model of P-selectin-mediated tumor cell-platelet association using labelled LLC cells w or w/out pre-treatment w 200 μg HS †	Molluscan HS inhibited lung metastasis (10 foci/lung control vs. 1 foci/lung HS; *p <* 0.05) w markedly smaller tumor size, and reduced heparinase activity (*p <* 0.05); reduced tumor–platelet complex to 30% (control 70%); similar effect to mammalian heparin at lower molar concentration; blocks both P-selectin-mediated interactions and heparinase activity blunting metastasis and inflammation	8 mg/kg	[274]
Pharidae	Body ‡	2 sialic acid-binding lectin recombinant proteins (rSgSABL-1, -2)	Non-specific pathogen immunisation model using mice (n = NA) immunised i.p. 2x w rSgSABL-1 (100 μg/mL) or rSgSABL-2 (180 μg/mL) in CFA	Antisera antibodies reactive with rSgSABL-1 and -2 in Western Blot Analysis; strong *Staphylococcus aureus* peptidoglycan (PAMP) binding affinity (*p <* 0.05 compared to PBS and pre-serum)	100–180 μg/mL	[188]
Veneridae	Body	(NH_4_)_2_SO_4_ fractionated peptide (‘Mere15′)	Human lung cancer (A549) xenograft model using Balb/c mice (n = 6/group) immunised s.c. w Mere15 12.5, 25.0 or 50.0 mg/kg (or cyclophosphamide [CTX] 50 mg/kg or normal saline) each day for 10 d	Mere15 at 25 and 50 mg/kg doses displayed 51% and 69% growth inhibition (*p <* 0.01), respectively; comparable to (though not sig dif than) CTX causing 53% inhibition; A549 most susceptible among cancer types in vitro therefore used in subsequent assays and animal model	25–50 mg/kg	[252]
**Gastropoda**						
Aplysiidae	Body	Dolastatin-10	Human small cell lung cancer (SCLC) (NCI-H446) xenograft model using CB-17 SCID mice (n = 8–10/group) treated w 450 μg/kg dolastatin 10 i.v. 26 and 36 d (or 7 and 17 d) after tumor inoculation	Treatments at 7 and 17 d completely inhibited tumor formation and increased survival (median 59 d control, >214 d treatment); treatments at 26 and 36 d (after tumor formation) caused tumor shrinkage (mass 1635 mg control, 44 mg treatment), growth delay, and increased survival (median 42 control, 91 treatment); pro-apoptotic mechanism	450 μg/kg	[254]
	Body	TZT-1027, (dolastatin 10 derivative)	Human LX-1 lung carcinoma xenograft model using Balb/c mice treated w 0.5, 1 and 2 mg/kg TZT-1027 (and Cisplatin- 5 and 10 mg/kg) administered i.v. after tumor established at 7 d, or both 7 and 14 d ^‖^	1–2 treatments caused tumor regression of 84–98% at 1 mg/kg, 99% at 2 mg/kg (> cisplatin: 49–52% at 5 mg/kg, 83% at 10 mg/kg); greater regression of lung cancer compared to breast cancer; 10% and 80% de-polymerisation of microtubule proteins 10% at 1.0 μM, 80% at 10 μM	1–2 mg/kg	[255]
Limacidae	Body	Aqueous Limax extract in MEM	COPD model using C57BL/6J mice (n = 8/group) treated w A) normal air + 2.18 g/kg extract; B) cigarette smoke (CS) + purified water; C) CS + 2.18 g/kg extract; CS = 9 cigarettes/h, 4 h per d, 6 d per wk in whole body exposure chamber for 90 d; extract given i.g. 0.5 h before daily CS exposure; cytotoxicity assay 0.01 μg/mL–10 mg/mL extract	CS-exposed Limax-treated mice improved pulmonary function compared to untreated mice (*p <* 0.05); less visual symptoms (e.g., weakness, wheezing), reduced lung damage, hyperplasia, inflammation, alveolar intercept and airway thickness (*p <* 0.01); reduced BALF inflammatory cell count (*p <* 0.01), inflammatory cytokines (*p* = 0.01–0.05) and Muc5AC secretion/expression (*p <* 0.01); suppression of inflammatory signaling cascades (*p <* 0.05); no sig cytotoxicity at effective doses; PPAR-γ enhancement and P38 MAPK pathway suppression	2.18 g/kg	[78]
	Body	Limax lyophilized powder H_2_O suspension	Allergic asthma model using guinea pigs (n = 15/group); sensitization using AlOH_2_ and egg albumin, treated w Limax (189, 63, 21 mg/kg/d) (or Aminophylline 80 mg/kg/d control); inhalation challenge after 7 d	Reduced asthma onset time, mortality, inflammatory markers (BALF/peripheral blood leukocyte count, eosinophil infiltration, IL-2 and IL-4) (*p <* 0.05); 63 mg/kg more effective than Aminophylline at reducing onset time (*p <* 0.05)	63 mg/kg	[140] ^¶^
	Body	Limax powder in H_2_O	Lewis lung carcinoma model using mice (n = 10/group); treatments 800–2500 mg/kg	Inhibitory effect on tumor growth (47% inhibition at 800 mg/kg) and prolonged survival (*p <* 0.01)	800 mg/kg	[111] ^#^
Muricidae	Hypobranchial gland (HBG)	Crude CHCl_3_-CH_3_OH extracts, 6-bromoisatin	Acute lung injury/inflammation model using C57Black/6 mice (n = 5–6/group) treated w HBG extract (0.5 or 0.1 mg/g), or 6-bromoisatin (0.05 or 0.1 mg/g) in 100 μL grape seed carrier oil (or PBS/carrier controls) administered p.o. 48 h, 24 h and 1 h prior to challenge w i.n. LPS (*E. coli*-derived) (1.25 mg/kg in 50 μL PBS)	Lower BALF total cells, neutrophils, TFN-α, IL-1β, and total protein in all treatments (*p <* 0.0001); 6-bromoisatin generally stronger effect (and lower concs used) but no sig difference between treatments; all doses of each compound significantly minimised all indicators of acute inflammatory damage to the lungs (*p <* 0.0001); positive correlation between histopathological scores and inflammatory markers (particularly TNF-α, IL-1β and neutrophils) in BALF (R^2^ 0.53–0.77, *p <* 0.0001)	0.5–0.1 mg/g HBG extract; 0.05–0.1 mg/g 6-bromoisatin	[77]
Muricidae **, Helicidae	Hemolymph	Experimentally purified Hc (RvH or HpH)	Colon cancer (C-26) model measuring lung metastasis in Balb/c mice (n = 20/group) sensitised i.p. w 200 μg RvH or HpH 2 wks before tumor inoculation and 100 μg weekly i.t.t. after solid tumor formation (sensitised) or 100 μg weekly i.t.t. only (non-sensitised); controls: PBS+challenge, RvH/HpH only no challenge	Lower surface lung metastases count in sens RvH, sens HpH and non-sens HpH groups; no sig dif. in cytokine profiles between groups; >anti-C-26 antibodies (*p <* 0.05); higher % survival in sens groups; >body weight in unsens groups (*p =* 0.001-0.05); reduced C-26 tumor size (*p <* 0.01) although all developed small tumors; HpH/RvH control survival NA	100 μg (w/w-out 200 μg dose pre-tumor formation)	[76]
Plakobranchid-ae	Mucus (originally)	PM02734 (Elisidepsin- synthetic KF3 derivative)	NSCLC (A549) model using NUR-NU-F-M mice (n = 5–7/group) treated w PM02734 (0.1 mg/kg × 3/wk for 2 wk i.v.), Erlotinib (50 mg/kg × 5/wk for 2 wks p.o.), combination (PM02734 i.v. 0.1 mg/kg × 3/wk for 2 wks i.v + Erlotinib p.o. 50 mg/kg × 5/wk for 2 wks p.o.), or no treatment; in vitro component used PM02734 0–10 uM	Combination treatment enhanced survival (>150 d) compared to PM02734 (54 d), Erlotinib (39) and control (23) (*p* = 0.0003–0.002); Erlotinib inhibited EGFR, AKT and ERK1/2 phosphorylation in vitro whereas PM02734 inhibited phosphorylation of ErbB3 and AKT and, to a lower extent, EGFR and ERK1/2 hence the efficacy of combined treatment	PM02734 0.1 mg/kg w or w/out 50 mg/kg Erlotinib	[263]

Abbreviations: Ab: antibody; w: with; PBS: phosphate buffer solution; CFA: Complete Freund’s Adjuvant; IFA: Incomplete Freund’s Adjuvant; OVA: ovalbumin; BALF: bronchoalveolar lavage fluid; LPS: lipopolysaccharide; PUFA: polyunsaturated fatty acid; PAMP: pathogen associated molecular pattern; MEM: Modified Eagles Medium; PAMP: pathogen associated microbial pattern. * Administration routes: i.p.- intraperitoneal, i.n.- intranasal, s.c.- subcutaneous, i.v.- intravenous, p.o.- per oral, i.t.t.- intratumoral, i.g.- intragastric. † Also included thioglycolate-induced peritoneal inflammation model and leukocyte rolling models. ‡ Described as body extract in text although lectins are commonly found in hemolymph. ^‖^ Also included a similar breast cancer model. ^¶^ Published in Chinese, translated by L. Liu, abstract available in English. ^#^ Published in Chinese, translated by L. Liu, abstract available in English. ** *Rapana venosa* originally listed as *R. thomasiana* (Crosse 1861).

**Table 8:** Human clinical trials using molluscan extracts and compounds related to the treatment of respiratory disease.

Mollusc Class Family	Derivative Part	Specific Extract/ Compound	Study Type and Design *	Main Findings	Effective Concentrations	Ref
**Gastropoda**						
Helicidae	Mucus	Helicidine	Double-bind, placebo-controlled, parallel-group clinical trial involving 30 COPD patients w history of chronic bronchitis and stabilised nocturnal cough (>20 cough episodes/night) treated w 2x 15-mL doses of 10% helicidine syrup (or placebo syrup) p.o. 3x daily for 3 d over 5-d observation period	Frequency of cough episodes/night reduced: 4.7–5.1 pre-treatment, 2.7–4.9 placebo, 1.3 helicidine group (*p <* 0.05); duration of cough period (during sleep and awakening) also reduced (*p <* 0.05); no sig difference between subjective endpoints (Spiegel questionnaire, CGI)	15-mL 10%	[80]
Plakobranchidae	Body (originally)	Kahalalide F (KF)	Phase I clinical trial and pharmacokinetic study involving 38 cancer patients (13 w lung cancer) administered i.v. 50 μg/mL kahalalide F weekly starting at 266 μg/m^2^ increasing between 25–100% over 21–109 cycles	Tumor shrinkage by 25–50% or stable disease in lung cancer patients; mild-moderate side effects w severe blood transaminase activity being the dose-limiting factor (3 cases); 650 μg/m^2^ recommended for future studies	650 μg/m^2^	[260]
	Body (originally)	KF	Non-randomised, multi-centre phase II clinical trial of KF as a second line therapy in 31 patients w advanced non-small cell lung cancer (NSCLC) administered i.v. 650 μg/m^2^ for 1 h/wk	One partial response observed; stable disease reported in 8 patients; majority of clinical benefit seen in patients with squamous cell carcinoma	650 μg/m^2^	[261,262]
	Body (originally)	PM02734 (Elisidepsin- synthetic KF3 derivative)	Phase 1 clinical trial and pharmacokinetic study involving 42 cancer patients (16 w lung cancer) administered i.v. 0.5 mg/m^2^ escalated at 100% increments (depending on grade of toxicity; median 2 cycles/patient, 3.2 mg/wk)	Disease stabilization in 12 patients (none w lung cancer), 1 patient (with metastatic esophageal adenocarcinoma) complete response; mild-moderate grade toxicities in ~17% of patients, grade 3 toxicities (hematologic, biochemical [transaminase]) in ~15% of patients lasting 7–14 d; necrosis-like cell-death	Max tolerable dose: 6.8 mg/m^2^	[259]
**Bivalvia**						
Mytilidae	Body	Lipid extract (‘Lyprinol’)	Double blind, randomised placebo-controlled parallel-group clinical trial involving 23 atopic asthma (mild-mod) patients (and 23 healthy subjects) treated w 2x 150 mg Lyprinol (or olive oil) capsules p.o. 2x daily for 8 weeks	Mean daytime wheeze and exhaled H_2_O_2_ sig reduced and morning PEF sig higher w Lyprinol treatment (*p <* 0.05); no differences in night awakenings and use of short-acting B2 agonist meds; inhibition of 5’-lipoxygenase and cyclo-oxygenase pathways responsible for production of eicosanoids	150 mg Lyprinol (50 mg extract in 100 mg olive oil)	[210]
	Body	Lipid extract (‘Lyprinol’)	Double blind, randomised placebo-controlled clinical trial using 73 (71 completed) children aged 6–13yrs treated w 2x 150 mg Lyprinol (or olive oil) capsules p.o. 2x daily for 7 months	Reduction in Fluticasone use (< 57.8 μg/d vs. 42.8 μg/d; *p* = 0.27), rescue β-agonist use (42.6% vs. 53.8%; *p* = 0.67); fewer asthma exacerbations (annualised rate of exacerbation 0.5 Lyprinol vs. 0.86 control); higher % reporting little/no trouble w their asthma (97 vs. 76%; *p* = 0.057); many other clinically important though non-significant improvements	150 mg Lyprinol (50 mg extract in 100 mg olive oil)	[209]
	Body	Lipid extract PCSO-524 (‘Lyprinol/OmegaXL’)	Double blind, randomised placebo-controlled clinical trial involving 20 patients w asthma and hyperpnea-induced bronchoconstriction treated w 8x 150 mg Lyprinol capsules p.o. daily for 8 weeks, followed by 2 weeks washout phase (usual diet) followed by 3-week special PCSO-524 diet phase (or usual diet control)	After the final phase, Lyprinol treatment and specific diet caused reduction in bronchodilator use and increase in mean morning and evening PEF (*p <* 0.05); no sif dif. in asthma symptom scores or FEV_1_; lower expired breath NO and urinary markers (*p <* 0.05)	150 mg Lyprinol (50 mg extract in 100 mg olive oil)	[207]

Abbreviations: Ab: antibody; Hc: hemocyanin; w: with; FEV_1_: forced expiratory volume; PEF: peak expiratory flow; COPD: chronic obstructive pulmonary disease; KLH: keyhole limpet hemocyanin. * Administration routes: s.c.- subcutaneous, i.v.- intravenous, i.m.- intramuscular, p.o.- per oral.

## 5. Molluscan Hemocyanins as Therapeutic Adjuvants and Model Antigens

When inoculated into mammals, molluscan Hcs have remarkable immunogenic effects: they generate a strong cellular reaction, promote high antibody levels and Th_1_/Th_2_ cytokine release, and prime antitumor CD8^+^ and CD4^+^ T cells [272] (Figure 3). Because of these versatile properties along with their large size (3.3–13.5 MDa), Hcs are regularly used as vaccine adjuvants and hapten carriers (subunit conjugates) [103]. As biomolecules, they are also considered safer than synthetic adjuvants (e.g., aluminium derivatives) [83]. Keyhole limpet hemocyanin (KLH) from the marine gastropod *Megathura crenulata* (Fissurellidae) is the most extensively used Hc, and is commercially available to biomedical researchers for vaccine development and immunological studies [103] (Tables S3 and S4). KLH has also been prescribed as an adjuvant in superficial bladder carcinoma therapy for over 30 years, and has shown potential in the treatment of other epithelial-derived adenocarcinomas, including of the lungs and respiratory tract [103].

There is ongoing interest in the inclusion of molluscan Hcs (mostly KLH) in vaccine preparations targeting respiratory tumors [300,301,302] and pathogens (including influenza [83,150,303,304,305], *P. aeruginosa* [166,306,307,308], *M. tuberculosis* [309], and respiratory syncytial virus [310]), as well as allergic asthma [311,312] (Table S3). Immunisation with molluscan Hcs conjugated to, or mixed with, a subunit antigen generates a significantly stronger antigen-specific antibody (particularly IgM and IgG) response compared to immunisation with the antigen alone [83,84,150,166,304,309] (Table S3). Hc-only control treatments demonstrate its immunogenicity with high anti-Hc antibody titers [83] and increased B and T lymphocyte proliferation [83], immune cell infiltration [310], cytokine (IL-4, IL-5, IFN-γ) expression [150,311], and opsonophagocytic activity [84] (Table S3). Several authors have proposed mechanisms by which Hcs exert such immunogenic effects [103,198,313].

There also exists a large body of research using molluscan Hcs (again, mostly KLH) as a model protein antigen, which has contributed to the assessment and understanding of immune responses involved in respiratory diseases (Table S4). For example, atopic asthmatics have been shown to produce more serum anti-Hc IgG4 [314] and IgE [30] than normal individuals, indicative of an increased/overactive Th_2_ response, while administration of molluscan Hcs to patients with respiratory cancer has revealed the immunosuppressive effects of chemotherapy and the disease itself [315,316] (Table S4). Sensitisation to KLH has been found to have an effect that is more-so protective than allergenic [317,318], but serum IL-4 and anti-KLH IgG4 and IgE increase in the presence of diesel exhaust particles [319], demonstrating the promotion effect of environmental pollutants on allergic airway diseases (Table S4). KLH has an excellent clinical safety profile [103] with no adverse events related to the use of this, or other Hcs, in any study reviewed for this paper (Table S3).

The versatile biomedical applications of KLH have led to increasing commercial demand and interest in obtaining novel Hcs with better/different immunogenicities [313]. Gesheva et al. [83,150] carried out two models whereby mice were immunised with influenza hemagglutinin subunit with adjuvant Hcs from *H. pomatia* (HpH) and *R. venosa* (RvH; Figure 3): although antibody and cytokine profiles varied, HpH produced immunogenic effects comparable to alum (AlOH_2_); RvH was comparable to KLH and Complete Freund’s Adjuvant, and ultimately superior to KLH in terms of spleenocyte cytotoxicity to virus-infected cells three-months post-immunisation (Table S3, Figure 2). HpH has also been used effectively as a model antigen in studies of asthma [30,320] and bronchial carcinoma [315] (Table S4). Hc from the Chilean abalone (*Concholepas concholepas*) is known to have immunogenic properties equal to or better than KLH; it is also commercially available and currently used in a clinical trial as an adjuvant in a prostate cancer vaccine [272].

## 6. Sustainable Supply and Traditional Knowledge Considerations

A critical question concerning natural product research is the technical and economic feasibility of obtaining large quantities of a given compound in a consistent and ecologically sound manner [321]. The ecology and life-history of a given species should be well-understood prior to intensive study and opportunities for non-lethal harvest should be sought where possible. Chemical synthesis of bioactive compounds is preferred for pharmaceutical supply due to the relative ease of quantification and purity assessment of active factors, although this is generally not suitable for nutraceuticals which imply a natural origin [141]. Sustainable production, with respect to both compound sources and other materials used in experimental processes, will be essential for the registration of new molluscan medicines by the FDA, Therapeutic Goods Administration, and counterpart authorities in developed countries.

The identification and collection of samples based on traditional knowledge adds a layer of legal complexity to the drug discovery process. For many years, it has been recognized that countries and indigenous groups have the right to take control of their biological property, and that the knowledge forming the basis of their traditional medical practices could be protected [322,323]. This is a contentious topic and opinions on its resolution vary widely. Nonetheless, as summarized well by Cordell & Colvard [323], acknowledging and compensating indigenous groups for their knowledge and for providing access to the local environment is a reasonable expectation for those who hold the resources, as well as those who seek them.

## 7. Conclusions: Promising Molluscan Extracts and Compounds for the Treatment and Prevention of Respiratory Disease

- This review highlights that there is a paucity of research on the bioactivity of molluscan extracts and compounds, considering the high diversity of species in this phylum and their merit as traditional medicines. Here, we have demonstrated the links between the anti-inflammatory, antimicrobial, anticancer, and immunomodulatory activity of molluscan extracts and compounds and their therapeutic potential in the prevention and treatment of respiratory diseases.
- At least 100 traditional medicines incorporating over 300 species of Mollusca have been used to treat respiratory diseases for thousands of years. Most of these are yet to receive research attention, and those few that have shown interesting bioactivities that validate some applications. There is a continued need to develop an evidence base toward the integration of quality-controlled traditional medicines.
- We identified particular incentive for biomedical research that elucidates anti-inflammatory factors from mollusc species/families comprising traditional medicines as there is likely to be some chemical basis consistent with their extensive use for alleviating inflammatory symptoms. Shell extracts are widely used but understudied and worthy of further investigation, as are certain taxonomic classes and families used in traditional medicines. The Polyplacophora and Scaphopoda, a wider range of Bivalvia including Veneridae, and shelled Gastropoda including Muricidae are of interest. Respiratory disease-focused ethnomedical studies would also be useful.
- Based on biomedical data, we expect that studies using molluscan compounds isolated from specialized glands, reproductive organs, microbial symbionts, and hemolymph would prove worthwhile.
- The exploration of novel molluscan Hcs holds good potential for the discovery of new antiviral and immunomodulatory agents, and therapeutic alternatives to KLH. Hcs could be tested in combination with other antiviral factors (e.g., zinc) from the same mollusc. Snail mucus should also be further investigated for anti-spasmodic, anti-inflammatory and anti-viral activities.
- Molluscan compounds may be valuable in the treatment of biofilm-associated respiratory infection and improve the efficacy of antibiotics. Further biofilm inhibition/disruption studies are needed and should be inclusive of *S. pneumoniae* and *P. aeruginosa*.
- Derivatives of KF and dolastatin 10 are the most potent molluscan anticancer compounds and of continued interest. Several compounds show anticancer activity and need to be tested against respiratory cancers (e.g., brominated indole/isatin derivatives; others in [66] and structural modifications). In vivo models of various cancer types should include measures of lung metastases and cytotoxicity to healthy cells.
- Compounds with bioactivities relevant to a range of respiratory diseases (e.g., anti-inflammatory activity) should be further explored, as well as combinations of compounds (molluscan-molluscan and molluscan-standard agents) to improve treatment efficacy for a single disease and address issues of chemotherapeutic and antimicrobial resistance.
- Overall, there is a need for more targeted research based on specific hypotheses related to respiratory disease using extracts and compounds derived from molluscs, with consideration to the sustainability of supply and attribution of traditional knowledge.

## Supplementary Materials

The following are available online at https://www.mdpi.com/1660-3397/18/11/570/s1, Table S1: Traditional medicinal uses of molluscs for respiratory related conditions used in ancient and modern cultures around the world appearing in ethnomedical texts and peer-reviewed articles. Table S2: Traditional Chinese medicines (TCMs) derived from molluscs relevant to the treatment of respiratory disease listed in the Chinese *Marine Materia Medica*. Table S3: Human clinical trials and in vivo animal models using molluscan hemocyanins as vaccine adjuvants/conjugates for respiratory disease. Table S4: In vivo animal models and human studies using molluscan hemocyanins as a model antigen for research investigating respiratory diseases. Table S5: Selected patents relating to pharmaceutical compounds derived from molluscs for the treatment of respiratory disease. Table S6: Classes of purified compounds tested against respiratory pathogens, cancers and inflammatory diseases.

## Common Abbreviations

TCMs	traditional Chinese medicines
OTMs	other traditional medicines
Hc	Hemocyanin
COPD	chronic obstructive pulmonary disease
ARDS	acute respiratory distress syndrome
NSAIDs	non-steroidal anti-inflammatory drugs
MIC/MBC	minimum inhibitory/bactericidal concentration
IC_50_/GI_50_/CC_50_	concentration causing 50% inhibition/growth inhibition/cytotoxicity
PUFAs	polyunsaturated fatty acids
FDA	United States Food and Drug Administration
Abs	antibodies

## References

[1] Amin K.A.M. (2015). Allergic respiratory inflammation and remodeling. Turk. Thorac. J..

[2] FIRS (2017). The Global Impact of Respiratory Disease.

[3] Jardins T.D., Burton G.G. (2016). Clinical Manifestations and Assessment of Respiratory Disease.

[4] Ferkol T., Schraufnagel D. (2014). The global burden of respiratory disease. Ann. Am. Thorac. Soc..

[5] Global Asthma Network (2018). The Global Asthma Report 2018.

[6] Forum of International Respiratory Societies (2013). Respiratory Diseases in the World.

[7] Ksiazek T.G., Erdman D., Goldsmith C.S., Zaki S.R., Peret T., Emery S., Tong S., Urbani C., Comer J.A., Lim W. (2003). A novel coronavirus associated with severe acute respiratory syndrome. N. Engl. J. Med..

[8] De Groot R.J., Baker S.C., Baric R.S., Brown C.S., Drosten C., Enjuanes L., Fouchier R.A.M., Galiano M., Gorbalenya A.E., Memish Z.A. (2013). Middle east respiratory syndrome coronavirus (MERS-CoV): Announcement of the coronavirus study group. J. Virol..

[9] Zhou P., Yang X.L., Wang X.G., Hu B., Zhang L., Zhang W., Si H.R., Zhu Y., Li B., Huang C.L. (2020). A pneumonia outbreak associated with a new coronavirus of probable bat origin. Nature.

[10] Moldoveanu B., Otmishi P., Jani P., Walker J., Sarmiento X., Guardiola J., Saad M., Yu J. (2009). Inflammatory mechanisms in the lung. J. Inflamm. Res..

[11] Ware L.B., Matthay M.A. (2000). The acute respiratory distress syndrome. N. Engl. J. Med..

[12] Murrow E.J., Oglesby F.M. (1996). Acute and chronic illness: Similarities, differences and challenges. Orthop. Nurs. Natl. Assoc. Orthop. Nurses.

[13] Riordan J.R., Rommens J.M., Kerem B.S., Alon N.O.A., Rozmahel R., Grzelczak Z., Zielenski J., Lok S.I., Plavsic N., Chou J.L. (1989). Identification of the cystic fibrosis gene: Cloning and characterization of complementary DNA. Science.

[14] Proud D., Chow C.W. (2006). Role of viral infections in asthma and chronic obstructive pulmonary disease. Am. J. Respir. Cell Mol. Biol..

[15] Umeki S. (1992). Re-evaluation of eosinophilic pneumonia and its diagnostic criteria. Arch. Intern. Med..

[16] Medzhitov R. (2010). Inflammation 2010: New adventures of an old flame. Cell.

[17] Kaminska B. (2005). MAPK signalling pathways as molecular targets for anti-inflammatory therapy: From molecular mechanisms to therapeutic benefits. Biochim. Biophys. Acta.

[18] Reber L.L., Hernandez J.D., Galli S.J. (2017). The pathophysiology of anaphylaxis. J. Allergy Clin. Immunol..

[19] Matthay M.A., Zemans R.L., Zimmerman G.A., Arabi Y.M., Beitler J.R., Mercat A., Herridge M., Randolph A.G., Calfee C.S. (2019). Acute respiratory distress syndrome. Nat. Rev. Dis. Primers.

[20] Engels E.A. (2008). Inflammation in the development of lung cancer: Epidemiological evidence. Expert Rev. Anticancer Ther..

[21] Bhatia M., Moochhala S. (2004). Role of inflammatory mediators in the pathophysiology of acute respiratory distress syndrome. J. Pathol..

[22] Störmann P., Lustenberger T., Relja B., Marzi I., Wutzler S. (2017). Role of biomarkers in acute traumatic lung injury. Injury.

[23] Galli S.J., Tsai M., Piliponsky A.M. (2008). The development of allergic inflammation. Nature.

[24] MacNee W. (2001). Oxidative stress and lung inflammation in airways disease. Eur. J. Pharmacol..

[25] Mizgerd J.P. (2008). Acute lower respiratory tract infection. N. Engl. J. Med..

[26] Worldometer COVID-19 Coronavirus Pandemic. https://www.worldometers.info/coronavirus/.

[27] World Health Organisation Fact Sheets. https://www.who.int/news-room/fact-sheets.

[28] Koh W.J. (2017). Nontuberculous Mycobacteria-Overview. Microbiol Spectr.

[29] Price D., Yawn B., Brusselle G., Rossi A. (2013). Risk-to-benefit ratio of inhaled corticosteroids in patients with COPD. Prim. Care Respir. J..

[30] Weller F.R., Kallenberg C.G.M., Jansen H.M., Torensma R., Klaassen R.J.L., Weller H.H., Orie N.G.M., The T.H. (1985). The primary immune response in bronchial asthma. I. A kinetic study of *Helix pomatia* hemocyanin-specific IgE, IgG, IgA, and IgM antibody responses in patients with asthma and in matched controls. J. Allergy Clin. Immunol..

[31] Vissers J.L.M., van Esch B.C.A.M., Hofman G.A., van Oosterhout A.J.M. (2005). Macrophages induce an allergen-specific and long-term suppression in a mouse asthma model. Eur. Respir. J..

[32] Bell S.C., Mall M.A., Gutierrez H., Macek M., Madge S., Davies J.C., Burgel P.-R., Tullis E., Castaños C., Castellani C. (2020). The future of cystic fibrosis care: A global perspective. Lancet Respir. Med..

[33] Geddes D. (2016). The history of respiratory disease management. Medicine.

[34] Acemoglu D., Johnson S. (2007). Disease and development: The effect of life expectancy on economic growth. J. Political Econ..

[35] Gonzales R., Malone D.C., Maselli J.H., Sande M.A. (2001). Excessive antibiotic use for acute respiratory infections in the United States. Clin. Infect. Dis..

[36] Laine L. (2001). Approaches to nonsteroidal anti-inflammatory drug use in the high-risk patient. Gastroenterology.

[37] Oyston P., Robinson K. (2012). The current challenges for vaccine development. J. Med. Microbiol..

[38] Vane J.R., Botting R.M. (1998). Anti-inflammatory drugs and their mechanism of action. Inflamm. Res..

[39] Poole K. (2005). Efflux-mediated antimicrobial resistance. J. Antimicrob. Chemother..

[40] Mader J.S., Hoskin D.W. (2006). Cationic antimicrobial peptides as novel cytotoxic agents for cancer treatment. Expert Opin. Investig. Drugs.

[41] Abdelmohsen U.R., Balasubramanian S., Oelschlaeger T.A., Grkovic T., Pham N.B., Quinn R.J., Hentschel U. (2017). Potential of marine natural products against drug-resistant fungal, viral, and parasitic infections. Lancet Infect. Dis..

[42] Santos L.H., Araújo A.N., Fachini A., Pena A., Delerue-Matos C., Montenegro M.C.B.S.M. (2010). Ecotoxicological aspects related to the presence of pharmaceuticals in the aquatic environment. J. Hazard. Mater..

[43] Corcoran J., Winter M.J., Tyler C.R. (2010). Pharmaceuticals in the aquatic environment: A critical review of the evidence for health effects in fish. Crit. Rev. Toxicol..

[44] Australian Government (2019). Australia’s National Antimicrobial Resistance Strategy-2020 and Beyond.

[45] Defer D., Bourgougnon N., Fleury Y. (2009). Screening for antibacterial and antiviral activities in three bivalve and two gastropod marine molluscs. Aquaculture.

[46] Yuan H., Ma Q., Ye L., Piao G. (2016). The traditional medicine and modern medicine from natural products. Molecules.

[47] Harvey A.L. (2007). Natural products as a screening resource. Curr. Opin. Chem. Biol..

[48] Esmaeelian B., Benkendorff K., Le Leu R.K., Abbott C.A. (2018). Simultaneous assessment of the efficacy and toxicity of marine mollusc–derived brominated indoles in an in vivo model for early stage colon cancer. Integr. Cancer Ther..

[49] Ahmad T.B., Liu L., Kotiw M., Benkendorff K. (2018). Review of anti-inflammatory, immune-modulatory and wound healing properties of molluscs. J. Ethnopharmacol..

[50] Jaspars M., De Pascale D., Andersen J.H., Reyes F., Crawford A.D., Ianora A. (2016). The marine biodiscovery pipeline and ocean medicines of tomorrow. J. Mar. Biol. Assoc. UK.

[51] Abadines I.B., Le K., Newman D.J., Glaser K.B., Mayer A.M. (2019). The marine pharmacology and pharmaceuticals pipeline in 2018. FASEB J..

[52] Mayer A.M., Glaser K.B., Cuevas C., Jacobs R.S., Kem W., Little R.D., McIntosh J.M., Newman D.J., Potts B.C., Shuster D.E. (2010). The odyssey of marine pharmaceuticals: A current pipeline perspective. Trends Pharmacol. Sci..

[53] Benkendorff K. (2010). Molluscan biological and chemical diversity: Secondary metabolites and medicinal resources produced by marine molluscs. Biol. Rev..

[54] Pyron M., Brown K., Thorp J.H., Rogers D.C. (2015). Introduction to Mollusca and the class Gastropoda (ch. 18). Ecology and General Biology: Thorp and Covich’s Freshwater Invertebrates.

[55] Bouchet P., Duarte C.M. (2006). The magnitude of marine biodiversity. The Exploration of Marine Biodiveristy, Scientific and Technological Challenges.

[56] Dang V.T., Benkendorff K., Green T., Speck P. (2015). Marine snails and slugs: A great place to look for antiviral drugs. J. Virol..

[57] Coutellec M.-A., Caquet T., Saleuddin S., Mukai S. (2016). Gastropod ecophysiological response to stress (ch. 9). Physiology of Molluscs: A Collection of Selected Reviews (Vol. 1).

[58] Ammerman J., Fuhrman J., Hagström A., Azam F. (1984). Bacterioplankton growth in seawater: I. Growth kinetics and cellular characteristics in seawater cultures. Mar. Ecol. Prog. Ser..

[59] Whitman W.B., Coleman D.C., Wiebe W.J. (1998). Prokaryotes: The unseen majority. Proc. Natl. Acad. Sci. USA.

[60] Raynaud X., Nunan N. (2014). Spatial ecology of bacteria at the microscale in soil. PLoS ONE.

[61] Hooper C., Day R., Slocombe R., Handlinger J., Benkendorff K. (2007). Stress and immune responses in abalone: Limitations in current knowledge and investigative methods based on other models. Fish Shellfish. Immunol..

[62] Cummins S.F., Nichols A.E., Schein C.H., Nagle G.T. (2006). Newly identified water-borne protein pheromones interact with attractin to stimulate mate attraction in *Aplysia*. Peptides.

[63] Bornancin L., Bonnard I., Mills S.C., Banaigs B. (2017). Chemical mediation as a structuring element in marine gastropod predator-prey interactions. Nat. Prod. Rep..

[64] Jiang M., Zhao C., Yan R., Li J., Song W., Peng R., Han Q., Jiang X. (2019). Continuous inking affects the biological and biochemical responses of cuttlefish *Sepia pharaonis*. Front. Physiol..

[65] Benkendorff K., Davis A.R., Bremner J. (2001). Chemical defense in the egg masses of benthic invertebrates: An assessment of antibacterial activity in 39 mollusks and 4 polychaetes. J. Invertebr. Pathol..

[66] Ciavatta M.L., Lefranc F., Carbone M., Mollo E., Gavagnin M., Betancourt T., Dasari R., Kornienko A., Kiss R. (2017). Marine mollusk-derived agents with antiproliferative activity as promising anticancer agents to overcome chemotherapy resistance. Med. Res. Rev..

[67] Benkendorff K., Cosmo A.D., Winlow W. (2014). Chemical diversity in molluscan communities: From natural products to chemical ecology. Neuroecology and Neuroethology in Molluscs: The Interface between Behaviour and Environment.

[68] Blunt J.W., Copp B.R., Keyzers R.A., Munro M.H., Prinsep M.R. (2017). Marine natural products. Nat. Prod. Rep..

[69] Blunt J.W., Copp B.R., Keyzers R.A., Munro M.H., Prinsep M. (2016). Marine natural products. Nat. Prod. Rep..

[70] Blunt J.W., Carroll A.R., Copp B.R., Davis R.A., Keyzers R.A., Prinsep M.R. (2018). Marine natural products. Nat. Prod. Rep..

[71] Carroll A.R., Copp B.R., Davis R.A., Keyzers R.A., Prinsep M.R. (2019). Marine natural products. Nat. Prod. Rep..

[72] Carroll A.R., Copp B.R., Davis R.A., Keyzers R.A., Prinsep M.R. (2020). Marine natural products. Nat. Prod. Rep..

[73] Dolashka P., Dolashki A., Van Beeumen J., Floetenmeyer M., Velkova L., Stevanovic S., Voelter W. (2016). Antimicrobial activity of molluscan hemocyanins from *Helix* and *Rapana* snails. Curr. Pharm. Biotechnol..

[74] Benkendorff K., Davis A.R., Rogers C.N., Bremner J.B. (2005). Free fatty acids and sterols in the benthic spawn of aquatic molluscs, and their associated antimicrobial properties. J. Exp. Mar. Biol. Ecol..

[75] Vine K.L., Locke J.M., Ranson M., Benkendorff K., Pyne S.G., Bremner J.B. (2007). In vitro cytotoxicity evaluation of some substituted isatin derivatives. Bioorg. Med. Chem..

[76] Gesheva V., Chausheva S., Mihaylova N., Manoylov I., Doumanova L., Idakieva K., Tchorbanov A. (2014). Anti-cancer properties of gastropodan hemocyanins in murine model of colon carcinoma. BMC Immunol..

[77] Ahmad T.B., Rudd D., Benkendorff K., Mahdi L.K., Pratt K.A., Dooley L., Wei C., Kotiw M. (2017). Brominated indoles from a marine mollusc inhibit inflammation in a murine model of acute lung injury. PLoS ONE.

[78] Liang X., Wang J., Guan R., Zhao L., Li D., Long Z., Yang Q., Xu J., Wang Z., Xie J. (2018). Limax extract ameliorates cigarette smoke-induced chronic obstructive pulmonary disease in mice. Int. Immunopharmacol..

[79] Pons F., Koenig M., Michelot R., Mayer M., Frossard N. (1999). The bronchorelaxant effect of helicidine, a *Helix pomatia* extract, involves prostaglandin E2 release. Pathol. Biol..

[80] Sergysels R., Art G. (2001). A double-masked, placebo-controlled polysomnographic study of the antitussive effects of helicidine. Curr. Ther. Res. Clin. Exp..

[81] Erspamer V., Glasser A. (1957). The pharmacological actions of murexine (urocanylcholine). Br. J. Pharmacol. Chemother..

[82] Badiu D.L., Luque R., Dumitrescu E., Craciun A., Dinca D. (2010). Amino acids from Mytilus galloprovincialis (L.) and Rapana venosa molluscs accelerate skin wounds healing via enhancement of dermal and epidermal neoformation. Protein J..

[83] Gesheva V., Chausheva S., Stefanova N., Mihaylova N., Doumanova L., Idakieva K., Tchorbanov A. (2015). *Helix pomatia* hemocyanin - a novel bio-adjuvant for viral and bacterial antigens. Int. Immunopharmacol..

[84] Theilacker C., Coleman F.T., Mueschenborn S., Llosa N., Grout M., Pier G.B. (2003). Construction and characterization of a *Pseudomonas aeruginosa* mucoid exopolysaccharide-alginate conjugate vaccine. Infect. Immun..

[85] Mayer A.M. Clinical Pipeline: Marine Pharmacology. https://www.midwestern.edu/departments/marinepharmacology/clinical-pipeline.xml.

[86] McGivern J.G. (2007). Ziconotide: A review of its pharmacology and use in the treatment of pain. Neuropsychiatr. Dis. Treat..

[87] Scott L.J. (2017). Brentuximab vedotin: A review in CD30-positive Hodgkin lymphoma. Drugs.

[88] Deeks E.D. (2019). Polatuzumab vedotin: First global approval. Drugs.

[89] Rosenberg J.E., O’Donnell P.H., Balar A.V., McGregor B.A., Heath E.I., Yu E.Y., Galsky M.D., Hahn N.M., Gartner E.M., Pinelli J.M. (2019). Pivotal trial of enfortumab vedotin in urothelial carcinoma after platinum and anti-programmed death 1/programmed death ligand 1 therapy. J. Clin. Oncol..

[90] Benkendorff K., Rudd D., Nongmaithem B.D., Liu L., Young F., Edwards V., Avila C., Abbott C.A. (2015). Are the traditional medical uses of muricidae molluscs substantiated by their pharmacological properties and bioactive compounds?. Mar. Drugs.

[91] Bonnemain B. (2005). Helix and drugs: Snails for western health care from antiquity to the present. Evid. Based Complement. Altern. Med..

[92] Straus S.E. (2000). Complementary and alternative medicine: Challenges and opportunities for American medicine. Acad. Med..

[93] Cragg G.M., Boyd M.R., Cardellina J.H., Newman D.J., Snader K.M., McCloud T.G. (1994). Ethnobotany and drug discovery: The experience of the US National Cancer Institute. Ciba Found. Symp..

[94] Lee M.R. (2011). The history of Ephedra (ma-huang). J. R. Coll. Physicians Edinb..

[95] Zhukova N.V. (2019). Fatty acids of marine mollusks: Impact of diet, bacterial symbiosis and biosynthetic potential. Biomolecules.

[96] Sofi F., Abbate R., Gensini G.F., Casini A. (2010). Accruing evidence on benefits of adherence to the Mediterranean diet on health: An updated systematic review and meta-analysis. Am. J. Clin. Nutr..

[97] Lordan S., Ross R.P., Stanton C. (2011). Marine bioactives as functional food ingredients: Potential to reduce the incidence of chronic diseases. Mar. Drugs.

[98] Chatzi L., Kogevinas M. (2009). Prenatal and childhood Mediterranean diet and the development of asthma and allergies in children. Public Health Nutr..

[99] Sorlí-Aguilar M., Martín-Luján F., Santigosa-Ayala A., Piñol-Moreso J.L., Flores-Mateo G., Basora-Gallisà J., Arija-Val V., Solà-Alberich R. (2015). Effects of Mediterranean diet on lung function in smokers: A randomised, parallel and controlled protocol. BMC Public Health.

[100] Hageman J.H., Hooyenga P., Diersen-Schade D.A., Scalabrin D.M.F., Wichers H.J., Birch E.E. (2012). The impact of dietary long-chain polyunsaturated fatty acids on respiratory illness in infants and children. Curr. Allergy Asthma Rep..

[101] Shahar E., Folsom A.R., Melnick S.L., Tockman M.S., Comstock G.W., Gennaro V., Higgins M.W., Sorlie P.D., Ko W.-J., Szklo M. (1994). Dietary n-3 polyunsaturated fatty acids and smoking-related chronic obstructive pulmonary disease. N. Engl. J. Med..

[102] Zanjani N.T., Saksena M.M., Dehghani F., Cunningham A.L. (2018). From ocean to bedside: The therapeutic potential of molluscan hemocyanins. Curr. Med. Chem..

[103] Harris J.R., Markl J. (1999). Keyhole limpet hemocyanin (KLH): A biomedical review. Micron.

[104] Guan H.S., Wang S.G. (2009). Chinese Marine Materia Medica.

[105] WoRMS World Register of Marine Species. http://www.marinespecies.org/aphia.php?p=search.

[106] Asta Lakshmi S. (2011). Wonder molluscs and their utilities. Int. J. Pharm. Sci. Rev. Res..

[107] Neto N.A.L., Voeks R.A., Dias T.L.P., Alves R.R.N. (2012). Mollusks of Candomblé: Symbolic and ritualistic importance. J. Ethnobiol. Ethnomed..

[108] Herbert D., Hamer M., Mander M., Mkhize N., Prins F. (2003). Invertebrate animals as a component of the traditional medicine trade in KwaZulu-Natal, South Africa. Afr. Invertebr..

[109] Voultsiadou Eleni E. (2010). Therapeutic properties and uses of marine invertebrates in the ancient Greek world and early Byzantium. J. Ethnopharmacol..

[110] Sun B.N., Shen H.D., Wu H.X., Yao L.X., Cheng Z.Q., Diao Y. (2014). Determination of chemical constituents of the marine pulmonate slug, *Paraoncidium Reevesii*. Trop. J. Pharm. Res..

[111] Guo Y.F., Wu X.P., Liu F.Z. (1989). Antitumor effect of Limax in tumor-bearing mice. Chin. J. Mod. Dev. Tradit. Med..

[112] Barberis I., Bragazzi N.L., Galluzzo L., Martini M. (2017). The history of tuberculosis: From the first historical records to the isolation of Koch’s bacillus. J. Prev. Med. Hyg..

[113] Alves R.R., Alves H.N. (2011). The faunal drugstore: Animal-based remedies used in traditional medicines in Latin America. J. Ethnobiol. Ethnomed..

[114] Gopal R., Vijayakumaran M., Venkatesan R., Kathiroli S. (2008). Marine organisms in Indian medicine and their future prospects. Nat. Prod. Radiance.

[115] Jamir N., Lal P. (2005). Ethnozoological practices among Naga tribes. Indian J. Tradit. Knowl..

[116] Shilabin A.G., Kasanah N., Wedge D.E., Hamann M.T. (2007). Lysosome and HER3 (ErbB3) selective anticancer agent kahalalide F: Semisynthetic modifications and antifungal lead-exploration studies. J. Med. Chem..

[117] Lin Z., Koch M., Pond C.D., Mabeza G., Seronay R.A., Concepcion G.P., Barrows L.R., Olivera B.M., Schmidt E.W. (2014). Structure and activity of lobophorins from a turrid mollusk-associated Streptomyces sp.. J. Antibiot..

[118] Brieger J.E. (1960). Calcarea carbonica or Ostrea edulis?. Br. Homeopath. J..

[119] Ramchandani N.M. (2010). Homoeopathic treatment of upper respiratory tract infections in children: Evaluation of thirty case series. Complement. Ther. Clin. Pract..

[120] Colin P. (2006). Homeopathy and respiratory allergies: A series of 147 cases. Homeopathy.

[121] Alves R.R., Rosa I.L. (2007). Zootherapeutic practices among fishing communities in North and Northeast Brazil: A comparison. J. Ethnopharmacol..

[122] Meyer-Rochow V.B. (2017). Therapeutic arthropods and other, largely terrestrial, folk-medicinally important invertebrates: A comparative survey and review. J. Ethnobiol. Ethnomed..

[123] Alade G.O., Frank A., Ajibesin K.K. (2018). Animals and animal products as medicines: A survey of Epie-Atissa and Ogbia people of Bayelsa State, Nigeria. J. Pharm. Pharmacogn. Res..

[124] Meyerhof M., Sobhy G.P. (1932). The Abridged Version of “The Book of Simple Drugs”, of Ahmad Ibn Muhammad Al-Ghafiqi by Gregorius Abul-Farag.

[125] Prabhakar A.K., Roy S.P. (2009). Ethno-medicinal uses of some shell fishes by people of Kosi river basin of North-Bihar, India. Stud. Ethno Med..

[126] Nongmaithem B.D., Mouatt P., Smith J., Rudd D., Russell M., Sullivan C., Benkendorff K. (2017). Volatile and bioactive compounds in opercula from Muricidae molluscs supports their use in ceremonial incense and traditional medicines. Sci. Rep..

[127] Lev E., Zohar A. (2008). Practical Materia Medica of the Medieval Eastern Mediterranean according to the Cairo Genizah.

[128] Kim H., Song M.J. (2013). Ethnozoological study of medicinal animals on Jeju Island, Korea. J. Ethnopharmacol..

[129] Chinlampianga M., Singh R.K., Shukla A.C. (2013). Ethnozoological diversity of Northeast India: Empirical learning with traditional knowledge holders of Mizoram and Arunachal Pradesh. Indian J. Tradit. Knowl..

[130] Alves R.R., Rosa I.L. (2007). Zootherapy goes to town: The use of animal-based remedies in urban areas of NE and N Brazil. J. Ethnopharmacol..

[131] McHugh J. (2013). Blattes de byzance in India: Mollusk opercula and the history of perfumery. J. R. Asiat. Soc..

[132] Cragg G.M., Newman D.J. (2013). Natural products: A continuing source of novel drug leads. Biochim. Biophys. Acta Gen. Subj..

[133] HPUS (1878). Homeopathic Materia Medica of the United States.

[134] Immanuel G., Thaddaeus B.J., Usha M., Ramasubburayan R., Prakash S., Palavesam A. (2012). Antipyretic, wound healing and antimicrobial activity of processed shell of the marine mollusc *Cypraea moneta*. Asian Pac. J. Trop. Biomed..

[135] Hajji S., Younes I., Rinaudo M., Jellouli K., Nasri M. (2015). Characterization and in vitro evaluation of cytotoxicity, antimicrobial and antioxidant activities of chitosans extracted from three different marine sources. Appl. Biochem. Biotechnol..

[136] Shanmugam A., Kathiresan K., Nayak L. (2016). Preparation, characterization and antibacterial activity of chitosan and phosphorylated chitosan from cuttlebone of *Sepia kobiensis* (Hoyle, 1885). Biotechnol. Rep..

[137] Oakes F.R. (2005). Non-Lethal Method for Extracting Crude Hemocyanin from Gastropod Molluscs. U.S. Patent.

[138] Quevauviller A., Mainil J., Garcet S. (1953). Le mucus d’*Hélix pomatia* L.-préparation, composition, propriétés thérapeutiques et pharmacodynamiques. Rev. Pathol. Gen. Physiol. Clin..

[139] Greenberg A.K., Basu S., Hu J., Yie T.A., Tchou-Wong K.M., Rom W.N., Lee T.C. (2002). Selective p38 activation in human non-small cell lung cancer. Am. J. Respir. Cell Mol. Biol..

[140] Yan P.K., Lin G.Q., Luo Q.F., Xie J.K. (2011). Effect of Limax lyophilized powder on bronchial asthma. J. Chin. Med. Mater..

[141] Benkendorff K. (2013). Natural product research in the Australian marine invertebrate *Dicathais Orbita*. Mar. Drugs.

[142] Roseghini M., Severini C., Erspamer G.F., Erspamer V. (1996). Choline esters and biogenic amines in the hypobranchial gland of 55 molluscan species of the neogastropod Muricoidea superfamily. Toxicon.

[143] Westley C., Benkendorff K. (2008). Sex-specific Tyrian purple genesis: Precursor and pigment distribution in the reproductive system of the marine mollusc, *Dicathais Orbita*. J. Chem. Ecol..

[144] Cooksey C.J. (2001). Tyrian purple: 6, 6′-dibromoindigo and related compounds. Molecules.

[145] Bailey K.C. (1932). The Elder Pliny’s Chapters on Chemical Subjects.

[146] Edwards V., Benkendorff K., Young F. (2012). Marine compounds selectively induce apoptosis in female reproductive cancer cells but not in primary-derived human reproductive granulosa cells. Mar. Drugs.

[147] Esmaeelian B. (2014). In Vitro and In Vivo Testing of Purified Muricid Mollusc Extract on Colorectal Cancer. Ph.D. Thesis.

[148] Sklirou A.D., Gaboriaud-Kolar N., Papassideri I., Skaltsounis A.-L., Trougakos I.P. (2017). 6-bromo-indirubin-3′-oxime (6BIO), a Glycogen synthase kinase-3β inhibitor, activates cytoprotective cellular modules and suppresses cellular senescence-mediated biomolecular damage in human fibroblasts. Sci. Rep..

[149] Benkendorff K., Bremner J.B., Davis A.R. (2000). Tyrian purple precursors in the egg masses of the Australian muricid, *Dicathais orbita*: A possible defensive role. J. Chem. Ecol..

[150] Gesheva V., Idakieva K., Kerekov N., Nikolova K., Mihaylova N., Doumanova L., Tchorbanov A. (2011). Marine gastropod hemocyanins as adjuvants of non-conjugated bacterial and viral proteins. Fish Shellfish Immunol..

[151] Ahmad T.B., Rudd D., Kotiw M., Liu L., Benkendorff K. (2019). Correlation between fatty acid profile and anti-inflammatory activity in common Australian seafood by-products. Mar. Drugs.

[152] Reynolds P.D. (2002). The Scaphopoda. Adv. Mar. Biol..

[153] De Toledo-Piza A.R., de Oliveira M.I., Negri G., Mendonca R.Z., Figueiredo C.A. (2018). Polyunsaturated fatty acids from *Phyllocaulis boraceiensis* mucus block the replication of influenza virus. Arch. Microbiol..

[154] De Toledo-Piza A.R., Figueiredo C.A., de Oliveira M.I., Negri G., Namiyama G., Tonelotto M., Villar K.D., Rofatto H.K., Mendonca R.Z. (2016). The antiviral effect of mollusk mucus on measles virus. Antivir. Res..

[155] Szabó K., Amesbury J.R. (2011). Molluscs in a world of islands: The use of shellfish as a food resource in the tropical island Asia-Pacific region. Quat. Int..

[156] Dortch C.E., Kendrick G.W., Morse K. (1984). Aboriginal mollusc exploitation in southwestern Australia. Archaeol. Ocean..

[157] Bodeker G., Ong C.-K. (2005). WHO Global Atlas of Traditional, Complementary and Alternative Medicine.

[158] Allaertaert F.A., Villet S., Vincent S., Sauve L. (2018). Observational study on the dispensing of cough syrups to children with acute cough by community pharmacists in France. Minerva Pediatr..

[159] Fung F.Y., Linn Y.C. (2015). Developing traditional chinese medicine in the era of evidence-based medicine: Current evidences and challenges. Evid. Based Complement. Altern. Med..

[160] Morice A., Kardos P. (2016). Comprehensive evidence-based review on European antitussives. BMJ Open Respir. Res..

[161] Roch P. (1999). Defense mechanisms and disease prevention in farmed marine invertebrates. Aquaculture.

[162] Roch P., Hubert F., van Der Knaap W., Noël T. (1996). Present knowledge on the molecular basis of cytotoxicity, antibacterial activity and stress response in marine bivalves. Ital. J. Zool..

[163] Zhang G., Li X., Xue Z. (1999). Potential reasons and controlling strategies of mollusk dramatic death in China. Chin. Fish..

[164] Zannella C., Mosca F., Mariani F., Franci G., Folliero V., Galdiero M., Tiscar P.G., Galdiero M. (2017). Microbial diseases of bivalve mollusks: Infections, immunology and antimicrobial defense. Mar. Drugs.

[165] Yum H.K., Park I.N., Shin B.M., Choi S.J. (2014). Recurrent Pseudomonas aeruginosa infection in chronic lung diseases: Relapse or reinfection?. Tuberc. Respir. Dis..

[166] Sen-Kilic E., Blackwood C.B., Boehm D.T., Witt W.T., Malkowski A.C., Bevere J.R., Wong T.Y., Hall J.M., Bradford S.D., Varney M.E. (2019). Intranasal peptide-based fpva-klh conjugate vaccine protects mice from *Pseudomonas aeruginosa* acute murine pneumonia. Front. Immunol..

[167] Mitta G., Hubert F., Noel T., Roch P. (1999). Myticin, a novel cysteine-rich antimicrobial peptide isolated from haemocytes and plasma of the mussel *Mytilus galloprovincialis*. Eur. J. Biochem..

[168] Domeneghetti S., Franzoi M., Damiano N., Norante R., El Halfawy N.M., Mammi S., Marin O., Bellanda M., Venier P. (2015). Structural and antimicrobial features of peptides related to Myticin C, a special defense molecule from the Mediterranean mussel *Mytilus galloprovincialis*. J. Agric. Food Chem..

[169] Elshahawi S.I., Trindade-Silva A.E., Hanora A., Han A.W., Flores M.S., Vizzoni V., Schrago C.G., Soares C.A., Concepcion G.P., Distel D.L. (2013). Boronated tartrolon antibiotic produced by symbiotic cellulose-degrading bacteria in shipworm gills. Proc. Natl. Acad. Sci. USA.

[170] Maselli V., Galdiero E., Salzano A.M., Scaloni A., Maione A., Falanga A., Naviglio D., Guida M., Di Cosmo A., Galdiero S. (2020). OctoPartenopin: Identification and preliminary characterization of a novel antimicrobial peptide from the suckers of *Octopus vulgaris*. Mar. Drugs.

[171] Chand S., Karuso P. (2017). Isolation and total synthesis of two novel metabolites from the fissurellid mollusc *Scutus antipodes*. Tetrahedron Lett..

[172] Pani A., Marongiu M.E., Obino P., Gavagnin M., La Colla P. (1991). Antimicrobial and antiviral activity of xylosyl-methylthio-adenosine, a naturally occurring analogue of methylthio-adenosine from *Doris verrucosa*. Experientia.

[173] Chellaram C., Edward J. (2009). Anti-inflammatory potential of coral reef associated gastropod, *Drupa Margariticola*. Indian J. Sci. Technol..

[174] Pitt S.J., Graham M.A., Dedi C.G., Taylor-Harris P.M., Gunn A. (2015). Antimicrobial properties of mucus from the brown garden snail *Helix aspersa*. Br. J. Biomed. Sci..

[175] Periyasamy N., Srinivasan M., Balakrishnan S. (2012). Antimicrobial activities of the tissue extracts of *Babylonia spirata* Linnaeus, 1758 (Mollusca: Gastropoda) from Thazhanguda, southeast coast of India. Asian Pac. J. Trop. Biomed..

[176] Miller B.W., Torres J.P., Tun J.O., Flores M.S., Forteza I., Rosenberg G., Haygood M.G., Schmidt E.W., Concepcion G.P. (2020). Synergistic anti-methicillin-resistant *Staphylococcus aureus* (MRSA) activity and absolute stereochemistry of 7,8-dideoxygriseorhodin C. J. Antibiot..

[177] Maurice N.M., Bedi B., Sadikot R.T. (2018). *Pseudomonas aeruginosa* biofilms: Host response and clinical implications in lung infections. Am. J. Respir. Cell Mol. Biol..

[178] Gasu E.N., Ahor H.S., Borquaye L.S. (2019). Peptide mix from *Olivancillaria hiatula* interferes with cell-to-cell communication in *Pseudomonas aeruginosa*. BioMed Res. Int..

[179] Gasu E.N., Ahor H.S., Borquaye L.S. (2018). Peptide extract from *Olivancillaria hiatula* exhibits broad-spectrum antibacterial activity. BioMed Res. Int..

[180] Ishwarya R., Vaseeharan B., Iswarya A., Karthikeyan S. (2016). Haemolytic and antibiofilm properties of haemocyanin purified from the haemolymph of Indian white shrimp *Fenneropenaeus indicus*. Fish Shellfish Immunol..

[181] Ishwarya R., Iswarya A., Thangaviji V., Sivakamavalli J., Esteban M.A., Thangaraj M.P., Vaseeharan B. (2020). Immunological and antibiofilm property of haemocyanin purified from grooved tiger shrimp (*Penaeus semisulcatus*): An in vitro and in silico approach. Microb. Pathog..

[182] Ishwarya R., Vaseeharan B., Jayakumar R., Ramasubramanian V., Govindarajan M., Alharbi N.S., Khaled J.M., Al-anbr M.N., Benelli G. (2018). Bio-mining drugs from the sea: High antibiofilm properties of haemocyanin purified from the haemolymph of flower crab *Portunus pelagicus* (L.) (Decapoda: Portunidae). Aquaculture.

[183] Van der Poll T., Opal S.M. (2009). Pathogenesis, treatment, and prevention of pneumococcal pneumonia. Lancet.

[184] Chellaram C., Sreenivasan R.S., Jonesh S., Anand T.P., Edward J.K.P. (2009). In vitro antibiotic bustle of coral reef associated gastropod, *Drupa Margariticola* (Broderip, 1832) of tuticorin coastal waters, Southeastern India. Biotechnology.

[185] Ramasamy P., Subhapradha N., Srinivasan A., Shanmugam V., Krishnamoorthy J., Shanmugam A. (2011). In vitro evaluation of antimicrobial activity of methanolic extract from selected species of Cephalopods on clinical isolates. Afr. J. Microbiol. Res..

[186] Defres S., Marwick C., Nathwani D. (2009). MRSA as a cause of lung infection including airway infection, community-acquired pneumonia and hospital-acquired pneumonia. Eur. Respir. J..

[187] Notariale R., Basile A., Montana E., Romano N.C., Cacciapuoti M.G., Aliberti F., Gesuele R., De Ruberto F., Sorbo S., Tenore G.C. (2018). Protamine-like proteins have bactericidal activity: The first evidence in *Mytilus galloprovincialis*. Acta Biochim. Pol..

[188] Wei X.M., Yang D.L., Li H.Y., Jiang H.L., Liu X.Q., Zhang Q., Yang J.L. (2018). Sialic acid-binding lectins (SABLs) from Solen grandis function as PRRs ensuring immune recognition and bacterial clearance. Fish Shellfish Immunol..

[189] Cilia G., Fratini F. (2018). Antimicrobial properties of terrestrial snail and slug mucus. J. Complement. Integr. Med..

[190] Destoumieux-Garzon D., Rosa R.D., Schmitt P., Barreto C., Vidal-Dupiol J., Mitta G., Gueguen Y., Bachere E. (2016). Antimicrobial peptides in marine invertebrate health and disease. Philos. Trans. R. Soc. Lond. B Biol. Sci..

[191] Hoang V.L.T., Kim S.K. (2013). Antimicrobial peptides from marine sources. Curr. Protein Pept. Sci..

[192] Lazcano-Pérez F., Román-González S.A., Sánchez-Puig N., Arreguín-Espinosa R. (2012). Bioactive peptides from marine organisms: A short overview. Protein Pept. Lett..

[193] Ageitos J.M., Sánchez-Pérez A., Calo-Mata P., Villa T.G. (2017). Antimicrobial peptides (AMPs): Ancient compounds that represent novel weapons in the fight against bacteria. Biochem. Pharmacol..

[194] Tincu J.A., Taylor S.W. (2004). Antimicrobial peptides from marine invertebrates. Antimicrob. Agents Chemother..

[195] Sable R., Parajuli P., Jois S. (2017). Peptides, peptidomimetics, and polypeptides from marine sources: A wealth of natural sources for pharmaceutical applications. Mar. Drugs.

[196] Carriel-Gomes M.C., Kratz J.M., Müller V.D.M., Barardi C.R.M., Simões C.M.O. (2006). Evaluation of antiviral activity in hemolymph from oysters *Crassostrea rhizophorae* and *Crassostrea gigas*. Aquat. Living Resour..

[197] Dolashka-Angelova P., Lieb B., Velkova L., Heilen N., Sandra K., Nikolaeva-Glomb L., Dolashki A., Galabov A.S., Van Beeumen J., Stevanovic S. (2009). Identification of glycosylated sites in *Rapana* hemocyanin by mass spectrometry and gene sequence, and their antiviral effect. Bioconjugate Chem..

[198] Swaminathan A., Lucas R.M., Dear K., McMichael A.J. (2014). Keyhole limpet haemocyanin-a model antigen for human immunotoxicological studies. Br. J. Clin. Pharmacol..

[199] Gai Z., Matsuno A., Kato K., Kato S., Khan M.R.I., Shimizu T., Yoshioka T., Kato Y., Kishimura H., Kanno G. (2015). Crystal Structure of the 3.8-MDa Respiratory Supermolecule Hemocyanin at 3.0 Å Resolution. Structure.

[200] Chakraborty K., Joy M. (2020). High-value compounds from the molluscs of marine and estuarine ecosystems as prospective functional food ingredients: An overview. Food Res. Int..

[201] Kumar A., Kubota Y., Chernov M., Kasuya H. (2020). Potential role of zinc supplementation in prophylaxis and treatment of COVID-19. Med. Hypotheses.

[202] Te Velthuis A.J.W., van den Worm S.H.E., Sims A.C., Baric R.S., Snijder E.J., van Hemert M.J. (2010). Zn^2+^ inhibits coronavirus and arterivirus rna polymerase activity in vitro and zinc ionophores block the replication of these viruses in cell culture. PLoS Pathog..

[203] Wessels I., Rolles B., Rink L. (2020). The potential impact of zinc supplementation on COVID-19 pathogenesis. Front. Immunol..

[204] Das U.N., Ramos E.J., Meguid M.M. (2003). Metabolic alterations during inflammation and its modulation by central actions of omega-3 fatty acids. Curr. Opin. Clin. Nutr. Metab. Care.

[205] Black P.N., Sharpe S. (1997). Dietary fat and asthma: Is there a connection?. Eur. Respir. J..

[206] Calder P.C. (2018). Very long-chain n-3 fatty acids and human health: Fact, fiction and the future. Proc. Nutr. Soc..

[207] Mickleborough T.D., Vaughn C.L., Shei R.J., Davis E.M., Wilhite D.P. (2013). Marine lipid fraction PCSO-524™ (Lyprinol^®^/Omega XL^®^) of the New Zealand green lipped mussel attenuates hyperpnea-induced bronchoconstriction in asthma. Respir. Med..

[208] Wood L.G., Hazlewood L.C., Foster P.S., Hansbro P.M. (2010). Lyprinol reduces inflammation and improves lung function in a mouse model of allergic airways disease. Clin. Exp. Allergy.

[209] Lello J., Liang A., Robinson E., Leutenegger D., Wheat A. (2012). Treatment of children’s asthma with a lipid extract of the new Zealand green lipped mussel (*Perna canaliculus*) (Lyprinol^®^)—A double blind, randomized controlled trial in children with moderate to serve chronic obstructive asthma. Internet J. Asthma Allergy Immunol..

[210] Emelyanov A., Fedoseev G., Krasnoschekova O., Abulimity A., Trendeleva T., Barnes P.J. (2002). Treatment of asthma with lipid extract of New Zealand green-lipped mussel: A randomised clinical trial. Eur. Respir. J..

[211] Thien F.C.K., De Luca S., Woods R.K., Abramson M.J. (2000). Dietary marine fatty acids (fish oil) for asthma in adults and children. Cochrane Database Syst. Rev..

[212] Ahmad T.B., Rudd D., Smith J., Kotiw M., Mouatt P., Seymour L.M., Liu L., Benkendorff K. (2017). Anti-inflammatory activity and structure-activity relationships of brominated indoles from a marine mollusc. Mar. Drugs.

[213] Yazbeck R., Lindsay R., Abbott C.A., Benkendorff K., Howarth G.S. (2015). Combined effects of muricid extract and 5-fluorouracil on intestinal toxicity in rats. Evid. Based Complement. Altern. Med..

[214] Esmaeelian B., Abbott C.A., Le Leu R.K., Benkendorff K. (2014). 6-Bromoisatin found in muricid mollusc extracts inhibits colon cancer cell proliferation and induces apoptosis, preventing early stage tumor formation in a colorectal cancer rodent model. Mar. Drugs.

[215] Westley C.B., Benkendorff K., McIver C.M., Le Leu R.K., Abbott C.A. (2013). Gastrointestinal and hepatotoxicity assessment of an anticancer extract from muricid molluscs. Evid. Based Complement. Altern. Med..

[216] Joung H.J., Kim Y.S., Hwang J.W., Han Y.K., Jeong J.H., Lee J.S., Moon S.H., Jeon B.T., Park P.J. (2014). Anti-inflammatory effects of extract from *Haliotis discus hannai* fermented with *Cordyceps militaris* mycelia in RAW264.7 macrophages through TRIF-dependent signaling pathway. Fish Shellfish Immunol..

[217] Qian Z.-J., Kim S.-A., Lee J.S., Kim H.-J., Choi I.L.W., Jung W.-K. (2012). The antioxidant and anti-inflammatory effects of abalone intestine digest, *Haliotis discus hannai* in RAW 264.7 macrophages. Biotechnol. Bioprocess Eng..

[218] Chen Z.C., Wu S.S., Su W.Y., Lin Y.C., Lee Y.H., Wu W.H., Chen C.H., Wen Z.H. (2016). Anti-inflammatory and burn injury wound healing properties of the shell of *Haliotis diversicolor*. BMC Complement. Altern. Med..

[219] Bhattacharya S., Chakraborty M., Bose M., Mukherjee D., Roychoudhury A., Dhar P., Mishra R. (2014). Indian freshwater edible snail *Bellamya bengalensis* lipid extract prevents T cell mediated hypersensitivity and inhibits LPS induced macrophage activation. J. Ethnopharmacol..

[220] Kao Y.F., Wu Y.H.S., Chou C.H., Fu S.G., Liu C.W., Chai H.J., Chen Y.C. (2018). Manufacture and characterization of anti-inflammatory liposomes from jumbo flying squid (*Dosidicus gigas*) skin phospholipid extraction. Food Funct..

[221] Pereira R.B., Taveira M., Valentão P., Sousa C., Andrade P.B. (2015). Fatty acids from edible sea hares: Anti-inflammatory capacity in LPS-stimulated RAW 264.7 cells involves iNOS modulation. RSC Adv..

[222] Jung H.-J., Nam K.N., Son M.-S., Kang H., Hong J.-W., Kim J.W., Lee E.H. (2011). Indirubin-3′-oxime inhibits inflammatory activation of rat brain microglia. Neurosci. Lett..

[223] Kim J.K., Park G.M. (2012). Indirubin-3-monoxime exhibits anti-inflammatory properties by down-regulating NF-κB and JNK signaling pathways in lipopolysaccharide-treated RAW264.7 cells. Inflamm. Res..

[224] Man Y., Wang Y.X., Zhu S.Y., Yang S., Zhao D., Hu F., Li J.Y. (2012). Indirubin inhibits ATP-induced phagocytosis attenuation, ROS production and cell death of macrophages. Yao Xue Xue Bao.

[225] Salas S., Chakraborty K. (2020). Polyether macrocyclic polyketide from the muricid gastropod *Chicoreus ramosus* attenuates pro-inflammatory 5-lipoxygenase. Med. Chem. Res..

[226] Chakraborty K., Salas S. (2019). First report of antioxidant 1H-benzochromenone from muricid gastropod *Chicoreus ramosus* as dual inhibitors of pro-inflammatory 5-lipoxygenase and carbolytic enzymes. Nat. Prod. Res..

[227] Chakraborty K., Salas S., Joy M. (2018). An unreported bis-abeo cembrane-type diterpenoid with antioxidative and anti-lipoxygenase activities from the muricid gastropod mollusc *Chicoreus ramosus*. Nat. Prod. Res..

[228] Chakraborty K., Krishnan S., Joy M. (2020). Polygalactan from bivalve *Crassostrea madrasensis* attenuates nuclear factor-κB activation and cytokine production in lipopolysaccharide-activated macrophage. Carbohydr. Polym..

[229] Chakraborty K., Joy M. (2019). Characterization and bioactive potentials of secondary metabolites from mollusks *Crassostrea madrasensis* and *Amphioctopus marginatus*. Nat. Prod. Res..

[230] Chakraborty K., Joy M., Chakkalakal S.J. (2019). Antioxidant and antiinflammatory secondary metabolites from the Asian green mussel *Perna viridis*. J. Food Biochem..

[231] Wu Y., Hu X., Song L., Zhu J., Yu R. (2014). The inhibitory effect of a novel polypeptide fraction from *Arca subcrenata* on cancer-related inflammation in human cervical cancer HeLa cells. Sci. World J..

[232] Joy M., Chakraborty K. (2018). Antioxidative and anti-inflammatory pyranoids and isochromenyl analogues from Corbiculid bivalve clam, *Villorita cyprinoides*. Food Chem..

[233] Sarkar A., Gomes A., Gomes A. (2015). Anti-osteoarthritis, anti-nociception, anti-inflammatory activities of isolated fraction of flesh extract *Viviparous bengalensis* in experimental model. Int. J. Curr. Res. Acad. Rev..

[234] Sarkar A., Gomes A., Gomes A. (2002). Anti-osteoporosis activity of fresh water snail (*Viviparous bengalensis*) flesh extracted protein fraction vb-p4 in rat models. Int. J. Curr. Res. Biosci. Plant Biol..

[235] Sarkar A., Datta P., Gomes A., Gupta S.C.D., Gomes A. (2013). Anti-osteoporosis and anti-osteoarthritis activity of fresh water snail (*Viviparous bengalensis*) flesh extract in experimental animal model. Open J. Rheumatol. Autoimmune Dis..

[236] Akerkar A.S., Ponkshe C.A., Indap M.M. (2009). Evaluation of immunomodulatory activity of extracts from marine animals. Indian J. Mar. Sci..

[237] Ponkshe C.A., Indap M.M. (2002). In vivo and in vitro evaluation for immunomodulatory activity of three marine animal extracts with reference to phagocytosis. Indian J. Exp. Biol..

[238] Chellaram C., Anand T.P.P., Kuberan G., John A.A., Priya G., Kumar B.A. (2012). Anti-inflammatory and analgesic effects of coral reef associated gastropod, *Trochus tentorium* from Tuticorin coastal waters, Southeastern India. Afr. J. Biotechnol..

[239] Santhi V., Sivakumar V., Thangathirupathi A., Thilaga R. (2011). Analgesic, anti-pyretic and anti-inflammatory activities of chloroform extract of prosobranch mollusc *Purpura Persica*. Int. J. Pharm. Biol. Sci..

[240] Santhi V., Sivakumar V., Thilaga R., Thangathirupathi A. (2012). Analgesic, antipyretic and anti inflammatory activities of column fraction of *Babylonia zeylanica* (Bruguiere, 1789) in albino rats. Int. J. Pharm. Biol. Sci..

[241] Joseph S.M., George M., Nair J.R., Senan V.P., Pillai D., Sherief P. (2005). Effect of feeding cuttlefish liver oil on immune function, inflammatory response and platelet aggregation in rats. Curr. Sci..

[242] Fei L., Xu K. (2016). Zhikang capsule ameliorates dextran sodium sulfate-induced colitis by inhibition of inflammation, apoptosis, oxidative stress and MyD88-dependent TLR4 signaling pathway. J. Ethnopharmacol..

[243] Chakraborty M., Bhattacharya S., Bhattacharjee P., Das R., Mishra R. (2010). Prevention of the progression of adjuvant induced arthritis by oral supplementation of Indian fresh water mussel (*Lamellidens marginalis*) aqueous extract in experimental rats. J. Ethnopharmacol..

[244] Swapna P. (2015). Anti Inflammatory, wound healing and analgesic activities of fresh water mollusc *Parreysia cylindrica* in albino rats. Indian J. Appl. Res..

[245] Li G., Fu Y., Zheng J., Li D. (2014). Anti-inflammatory activity and mechanism of a lipid extract from hard-shelled mussel (*Mytilus coruscus*) on chronic arthritis in rats. Mar. Drugs.

[246] Mimura T., Itoh S., Tsujikawa K., Nakajima H., Satake M., Kohama Y., Okabe M. (1987). Studies on biological activities of melanin from marine animals. V. Anti-inflammatory activity of low-molecular-weight melanoprotein from squid (Fr. SM II). Chem. Pharm. Bull..

[247] Zheng Y., Liu Z., Wang L., Li M., Zhang Y., Zong Y., Li Y., Song L. (2020). A novel tumor necrosis factor in the Pacific oyster *Crassostrea gigas* mediates the antibacterial response by triggering the synthesis of lysozyme and nitric oxide. Fish Shellfish Immunol..

[248] Agrawal S., Chaugule S., More S., Rane G., Indap M. (2017). Methanolic extract of *Euchelus asper* exhibits in-ovo anti-angiogenic and in vitro anti-proliferative activities. Biol. Res..

[249] Meivelu M., Seedevi P., Vairamani S., Shanmugam A. (2019). Exploring the chemical composition and anticancer potential of oil from squid (*Loligo duvauceli*) liver waste from fish processing industry. Waste Biomass Valorization.

[250] Liu J.T., Deng Z.H., Huang J.H., Wang Y., Chang J., Zhu D. (2014). Apple snails polysaccharide extraction and pharmacological potential study in vitro. Appl. Mech. Mat..

[251] Zhang L., Fan X., Han L. (2005). Antitumor and immune regulation activities of the extracts of some Chinese marine invertebrates. Chin. J. Oceanol. Limnol..

[252] Wang C., Liu M., Cheng L., Wei J., Wu N., Zheng L., Lin X. (2012). A novel polypeptide from *Meretrix meretrix* Linnaeus inhibits the growth of human lung adenocarcinoma. Exp. Biol. Med..

[253] Shilabin A.G., Hamann M.T. (2007). In vitro and in vivo evaluation of select kahalalide F analogs with antitumor and antifungal activities. Bioorg. Med. Chem..

[254] Kalemkerian G.P., Ou X., Adil M.R., Rosati R., Khoulani M.M., Madan S.K., Pettit G.R. (1999). Activity of dolastatin 10 against small-cell lung cancer in vitro and in vivo: Induction of apoptosis and bcl-2 modification. Cancer Chemother. Pharmacol..

[255] Kobayashi M., Natsume T., Tamaoki S., Watanabe J., Asano H., Mikami T., Miyasaka K., Miyazaki K., Gondo M., Sakakibara K. (1997). Antitumor activity of TZT-1027, a novel dolastatin 10 derivative. Jap. J. Cancer Res..

[256] Janmaat M.L., Rodriguez J.A., Jimeno J., Kruyt F.A.E., Giaccone G. (2005). Kahalalide F induces necrosis-like cell death that involves depletion of ErbB3 and inhibition of Akt signaling. Mol. Pharmacol..

[257] Ciavatta M.L., Devi P., Carbone M., Mathieu V., Kiss R., Casapullo A., Gavagnin M. (2016). Kahalalide F analogues from the mucous secretion of Indian sacoglossan mollusc *Elysia ornata*. Tetrahedron.

[258] Teixidó C., Arguelaguet E., Pons B., Aracil M., Jimeno J., Somoza R., Marés R., Ramón Y., Cajal S., Hernández-Losa J. (2012). ErbB3 expression predicts sensitivity to elisidepsin treatment: In vitro synergism with cisplatin, paclitaxel and gemcitabine in lung, breast and colon cancer cell lines. Int. J. Oncol..

[259] Salazar R., Jones R.J., Oaknin A., Crawford D., Cuadra C., Hopkins C., Gil M., Coronado C., Soto-Matos A., Cullell-Young M. (2012). A phase I and pharmacokinetic study of elisidepsin (PM02734) in patients with advanced solid tumors. Cancer Chemother. Pharmacol..

[260] Pardo B., Paz-Ares L., Tabernero J., Ciruelos E., Garcia M., Salazar R., Lopez A., Blanco M., Nieto A., Jimeno J. (2008). Phase I clinical and pharmacokinetic study of kahalalide F administered weekly as a 1-hour infusion to patients with advanced solid tumors. Clin. Cancer Res..

[261] Hamann M.T. (2004). Technology evaluation: Kahalalide F, PharmaMar. Curr. Opin. Mol. Ther..

[262] PharmaMar PharmaMar reports new data on Kahalalide-F and Aplidin(R) at ESMO congress. Proceedings of the 31st European Society for Medical Oncology Congress (ESMO).

[263] Ling Y.H., Aracil M., Jimeno J., Perez-Soler R., Zou Y. (2009). Molecular pharmacodynamics of PM02734 (elisidepsin) as single agent and in combination with erlotinib; synergistic activity in human non-small cell lung cancer cell lines and xenograft models. Eur. J. Cancer.

[264] Poncet J. (1999). The dolastatins, a family of promising antineoplastic agents. Curr. Pharm. Des..

[265] Salazar R., Cortes-Funes H., Casado E., Pardo B., Lopez-Martin A., Cuadra C., Tabernero J., Coronado C., Garcia M., Matos-Pita A.S. (2013). Phase I study of weekly kahalalide F as prolonged infusion in patients with advanced solid tumors. Cancer Chemother. Pharmacol..

[266] Pereira R.B., Andrade P.B., Valentao P. (2016). Chemical diversity and biological properties of secondary metabolites from sea hares of *Aplysia* genus. Mar Drugs.

[267] Lee W.-j., Lim H.-S. (2005). Two patients of acute liver damage following the ingestion of a sea hare eggs. J. Agric. Med. Comm. Health.

[268] Jamil A., Kasi A. (2020). Cancer: Metastasis to the Lung.

[269] Riihimäki M., Hemminki A., Fallah M., Thomsen H., Sundquist K., Sundquist J., Hemminki K. (2014). Metastatic sites and survival in lung cancer. Lung Cancer.

[270] Krishnan K., Khanna C., Helman L.J. (2006). The molecular biology of pulmonary metastasis. Thorac. Surg. Clin..

[271] Punt J., Stranford S., Jones P., Owen J. (2018). Kuby Immunology.

[272] Pizarro-Bauerle J., Maldonado I., Sosoniuk-Roche E., Vallejos G., López M.N., Salazar-Onfray F., Aguilar-Guzmán L., Valck C., Ferreira A., Becker M.I. (2017). Molluskan hemocyanins activate the classical pathway of the human complement system through natural antibodies. Front. Immunol..

[273] Kellar A., Egan C., Morris D. (2015). Preclinical murine models for lung cancer: Clinical trial applications. BioMed Res. Int..

[274] Gomes A.M., Kozlowski E.O., Borsig L., Teixeira F., Vlodavsky I., Pavao M.S.G. (2015). Antitumor properties of a new non-anticoagulant heparin analog from the mollusk *Nodipecten nodosus: Effect* on P-selectin, heparanase, metastasis and cellular recruitment. Glycobiology.

[275] Dietrich C.P., de Paiva J., Moraes C.T., Takahashi H.K., Porcionatto M.A., Nader H.B. (1985). Isolation and characterization of a heparin with high anticoagulant activity from *Anomalocardia brasiliana*. Biochim. Biophys. Acta Gen. Subj..

[276] Dietrich C.P., Tersariol I.L., Toma L., Moraes C.T., Porcionatto M.A., Oliveira F.W., Nader H.B. (1998). Structure of heparan sulfate: Identification of variable and constant oligosaccharide domains in eight heparan sulfates of different origins. Cell Mol. Biol..

[277] Arumugam M., Shanmugam A. (2004). Extraction of heparin and heparin-like substance from marine mesogastropod mollusc *Turritella attenuata* (Lamarck, 1779). Indian J. Exp. Biol..

[278] Pejler G., Danielsson A., Björk I., Lindahl U., Nader H., Dietrich C. (1987). Structure and antithrombin-binding properties of heparin isolated from the clams *Anomalocardia brasiliana* and *Tivela mactroides*. J. Biol. Chem..

[279] Esmaeelian B., Benkendorff K., Johnston M.R., Abbott C.A. (2013). Purified brominated indole derivatives from *Dicathais orbita* induce apoptosis and cell cycle arrest in colorectal cancer cell lines. Mar. Drugs.

[280] Benkendorff K., McIver C.M., Abbott C.A. (2011). Bioactivity of the Murex homeopathic remedy and of extracts from an Australian muricid mollusc against human cancer cells. Evid. Based Complement. Altern. Med..

[281] Nicolaou K.A., Liapis V., Evdokiou A., Constantinou C., Magiatis P., Skaltsounis A.L., Koumas L., Costeas P.A., Constantinou A.I. (2012). Induction of discrete apoptotic pathways by bromo-substituted indirubin derivatives in invasive breast cancer cells. Biochem. Biophys. Res. Commun..

[282] Edwards V., Young F., Benkendorff K. (2013). Effects of Muricidae Extracts on Estrogen-Sensitive Breast Cancer and the Steroidogenic Pathway.

[283] Choi S.J., Moon M.J., Lee S.D., Choi S.-U., Han S.-Y., Kim Y.-C., Letters M.C. (2010). Indirubin derivatives as potent FLT3 inhibitors with anti-proliferative activity of acute myeloid leukemic cells. Bioorg. Med. Chem. Lett..

[284] Saito H., Tabata K., Hanada S., Kanda Y., Suzuki T., Miyairi S. (2011). Synthesis of methoxy-and bromo-substituted indirubins and their activities on apoptosis induction in human neuroblastoma cells. Bioorg. Med. Chem. Lett..

[285] Liu L., Nam S., Tian Y., Yang F., Wu J., Wang Y., Scuto A., Polychronopoulos P., Magiatis P., Skaltsounis L. (2011). 6-Bromoindirubin-3′-oxime inhibits JAK/STAT3 signaling and induces apoptosis of human melanoma cells. J. Cancer Res..

[286] Fiebig H.H., Schuler J., Meijer L., Guyard N., Skaltsounis A.L., Eisenbrand G. (2006). In vivo anti-tumor activity of indirubins. Indirubin: The red shade of Indigo.

[287] Westley C.B., McIver C.M., Abbott C.A., Le Leu R.K., Benkendorff K. (2010). Enhanced acute apoptotic response to azoxymethane-induced DNA damage in the rodent colonic epithelium by Tyrian purple precursors: A potential colorectal cancer chemopreventative. Cancer Biol. Ther..

[288] Rudd D.A., Benkendorff K., Chahal C., Guinan T., Gustafsson O.J.R., Esmaeelian B., Krysinska H., Pogson L., Voelcker N.H., Abbott C.A. (2019). Mapping insoluble indole metabolites in the gastrointestinal environment of a murine colorectal cancer model using desorption/ionisation on porous silicon imaging. Sci. Rep..

[289] Edwards V., Benkendorff K., Young F. (2014). An in vitro high-throughput assay for screening reproductive and toxic effects of anticancer compounds. Biotechnol. Appl. Biochem..

[290] Karthik R., Manigandan V., Saravanan R. (2017). Toxicity, teratogenicity and antibacterial activity of posterior salivary gland (PSG) toxin from the cuttlefish *Sepia pharaonis* (Ehrenberg, 1831). J. Chromatogr. B Analyt. Technol. Biomed Life Sci..

[291] Karthik R., Saravanan R., Ebenezar K.K., Sivamalai T. (2014). Isolation, purification and characterization of avian antimicrobial glycopeptide from the posterior salivary gland of *Sepia pharaonis*. Appl. Biochem. Biotechnol..

[292] Zhong J., Wang W., Yang X., Yan X., Liu R. (2013). A novel cysteine-rich antimicrobial peptide from the mucus of the snail of *Achatina fulica*. Peptides.

[293] Hemu X., Tam J.P. (2017). Macrocyclic antimicrobial peptides engineered from ω-conotoxin. Curr. Pharm. Des..

[294] Dolashka P., Moshtanska V., Borisova V., Dolashki A., Stevanovic S., Dimanov T., Voelter W. (2011). Antimicrobial proline-rich peptides from the hemolymph of marine snail *Rapana venosa*. Peptides.

[295] Bitaab M.A., Ranaei Siadat S.O., Pazooki J., Sefidbakht Y. (2015). Antibacterial and molecular dynamics study of the Dolabellanin B2 isolated from sea slug, *Peronia peronii*. Biosci. Biotechnol. Res. Asia.

[296] Borquaye L.S., Darko G., Ocansey E., Ankomah E. (2015). Antimicrobial and antioxidant properties of the crude peptide extracts of *Galatea paradoxa* and *Patella rustica*. SpringerPlus.

[297] Rohini B., Priya C.S., Lavanya A., Kalpana K., Karthika V. (2012). Potential of water and methanol extracts of *Lambis lambis* against fish and human pathogens. Biol. Rhythm Res..

[298] Kanchana S., Vennila R., Rajesh Kumar K., Arumugam M., Balasubramanian T. (2014). Antagonistic and cyto-toxicity activity of mollusc methanol extracts. J. Biol. Sci..

[299] Silveira-Dorta G., Sousa I.J., Fernandes M.X., Martín V.S., Padrón J.M. (2015). Synthesis and identification of unprecedented selective inhibitors of CK1ε. Eur. J. Med. Chem..

[300] Dickler M.N., Ragupathi G., Liu N.X., Musselli C., Martino D.J., Miller V.A., Kris M.G., Brezicka F.T., Livingston P.O., Grant S.C. (1999). Immunogenicity of a fucosyl-GM1-keyhole limpet hemocyanin conjugate vaccine in patients with small cell lung cancer. Clin. Cancer Res..

[301] Miles D., Roché H., Martin M., Perren T.J., Cameron D.A., Glaspy J., Dodwell D., Parker J., Mayordomo J., Tres A. (2011). Phase III multicenter clinical trial of the sialyl-TN (STn)-keyhole limpet hemocyanin (KLH) vaccine for metastatic breast cancer. Oncologist.

[302] Hasumi K., Aoki Y., Watanabe R., Hankey K.G., Mann D.L. (2011). Therapeutic response in patients with advanced malignancies treated with combined dendritic cell-activated T cell based immunotherapy and intensity-modulated radiotherapy. Cancers.

[303] Hou Y., Gu X.-X. (2003). Development of peptide mimotopes of lipooligosaccharide from nontypeable *Haemophilus influenzae* as vaccine candidates. J. Immunol..

[304] Tan H.X., Jegaskanda S., Juno J.A., Esterbauer R., Wong J., Kelly H.G., Liu Y., Tilmanis D., Hurt A.C., Yewdell J.W. (2019). Subdominance and poor intrinsic immunogenicity limit humoral immunity targeting influenza HA stem. J. Clin. Investig..

[305] Kyd J.M., Cripps A.W., Novotny L.A., Bakaletz L.O. (2003). Efficacy of the 26-kilodalton outer membrane protein and two P5 fimbrin-derived immunogens to induce clearance of nontypeable *Haemophilus influenzae* from the rat middle ear and lungs as well as from the chinchilla middle ear and nasopharynx. Infect Immun..

[306] Hughes E.E., Gilleland L.B., Gilleland H.E. (1992). Synthetic peptides representing epitopes of outer membrane protein F of *Pseudomonas aeruginosa* that elicit antibodies reactive with whole cells of heterologous immunotype strains of *P. aeruginosa*. Infect Immun..

[307] Hughes E.E., Gilleland H.E. (1995). Ability of synthetic peptides representing epitopes of outer membrane protein F of *Pseudomonas aeruginosa* to afford protection against *P. aeruginosa* infection in a murine acute pneumonia model. Vaccine.

[308] Sokol P.A., Kooi C., Hodges R.S., Cachia P., Woods D.E. (2000). Immunization with a *Pseudomonas aeruginosa* elastase peptide reduces severity of experimental lung infections due to *P. aeruginosa* or *Burkholderia cepacia*. J. Infect Dis..

[309] Shin H.J., Franco L.H., Nair V.R., Collins A.C., Shiloh M.U. (2017). A baculovirus-conjugated mimotope vaccine targeting *Mycobacterium tuberculosis* lipoarabinomannan. PLoS ONE.

[310] Tebbey P.W., Hagen M., Hancock G.E. (1998). Atypical pulmonary eosinophilia is mediated by a specific amino acid sequence of the attachment (G) protein of respiratory syncytial virus. J. Exp. Med..

[311] Wallmann J., Epstein M.M., Singh P., Brunner R., Szalai K., El-Housseiny L., Pali-Schöll I., Jensen-Jarolim E. (2010). Mimotope vaccination for therapy of allergic asthma: Anti-inflammatory effects in a mouse model. Clin. Exp. Allergy.

[312] Demoly P., Persi L., Dhivert H., Delire M., Bousquet J. (2002). Immunotherapy with keyhole limpet hemocyanin-conjugated decapeptide vaccine in cypress pollen allergy. Clin. Exp. Allergy.

[313] Arancibia S., Campo M.D., Nova E., Salazar F., Becker M.I. (2012). Enhanced structural stability of Concholepas hemocyanin increases its immunogenicity and maintains its non-specific immunostimulatory effects. Eur. J. Immunol..

[314] Schuyler M., Lyons C.R., Masten B., Bice D. (1997). Immunoglobulin response to intrapulmonary immunization of asthmatics. Immunology.

[315] Jansen H.M., The T.H., de Gast G.C., Esselink M.T., Pastoor G., Orie N.G. (1978). The primary immune response of patients with different stages of squamous-cell bronchial carcinoma. Thorax.

[316] Curtis J.E., Hersh E.M., Harris J.E., McBride C., Freireich E.J. (1970). The human primary immune response to keyhole limpet haemocyanin: Interrelationships of delayed hypersensitivity, antibody response and in vitro blast transformation. Clin. Exp. Immunol..

[317] Krieger S.M., Boverhof D.R., Woolhiser M.R., Hotchkiss J.A. (2013). Assessment of the respiratory sensitization potential of proteins using an enhanced mouse intranasal test (MINT). Food Chem. Toxicol..

[318] Riedl M.A., Landaw E.M., Saxon A., Diaz-Sanchez D. (2005). Initial high-dose nasal allergen exposure prevents allergic sensitization to a neoantigen. J. Immunol..

[319] Diaz-Sanchez D., Garcia M.P., Wang M., Jyrala M., Saxon A. (1999). Nasal challenge with diesel exhaust particles can induce sensitization to a neoallergen in the human mucosa. J. Allergy Clin. Immunol..

[320] Weller F.R., Weller H.H., Kallenberg C.G.M., The T.H., Orie N.G.M. (1986). Sensitivity to hydrocortisone is a relevant factor in the immunoendocrine relationship. I. The cell-mediated immune response in relation to blood levels and in vitro immunosuppressive effects of hydrocortisone in patients with asthma and healthy control subjects. J. Allergy Clin. Immunol..

[321] Gomes A.M., Kozlowski E.O., Pomin V.H., de Barros C.M., Zaganeli J.L., Pavão M.S.G. (2010). Unique extracellular matrix heparan sulfate from the bivalve *Nodipecten nodosus* (Linnaeus, 1758) safely inhibits arterial thrombosis after photochemically induced endothelial lesion. J. Biol. Chem..

[322] Cragg G.M., Baker J.T., Borris R.P., Carte B., Cordell G.A., Soejarto D.D., Gupta M.P., Iwu M.M., Madulid D.A. (1997). Interactions with source countries: Guidelines for members of the American Society of Pharmacognosy. J. Nat. Prod..

[323] Cordell G.A., Colvard M.D. (2005). Some thoughts on the future of ethnopharmacology. J. Ethnopharmacol..

