# Supplementary material for: Molluscan Compounds Provide Drug Leads for the Treatment and Prevention of Respiratory Disease
Source: Mar Drugs. 2020 Nov 19;18(11):570. doi: 10.3390/md18110570 (PMC7699502; doi:10.3390/md18110570)
Supplement: Supplementary file 1 [file marinedrugs-18-00570-s001.pdf]

Review

Molluscan Compounds Provide Drug Leads for the Treatment and Prevention of Respiratory Disease

Kate Summer, Jessica Browne, Lei Liu and Kirsten Benkendorff

Supplementary information

Database search criteria

Databases Scopus, Web of Science and PubMed were searched for biomedical literature using search strings "mollusc OR mollusk OR molluscan OR hemocyanin OR haemocyanin" AND "antimicrobial OR antibacterial OR antiviral OR anti-inflammatory OR vaccine OR (lung AND cancer)" AND "in-vitro OR in-vivo OR (clinical AND trial)", which returned 861 hits minus duplicates (as of October, 2020). Additional targeted searches included "mollusc OR mollusk OR hemocyanin OR klh" AND "asthma OR copd OR bronchitis OR influenza OR pneumonia OR tuberculosis OR (respiratory AND disease)" and key respiratory pathogens including "(mycobacterium AND tuberculosis) OR pneumoniae OR (haemophilus AND influenzae) OR rhinovirus OR adenovirus OR (moraxella AND catarrhalis) OR (pseudemonas AND aeruginosa)".

For literature regarding traditional medicinal applications, database searches used the words: "mollusc OR mollusk OR molluscan" AND "traditional OR folk OR Indigenous OR Aboriginal OR historical OR natural" AND "medicine OR ethnomedicine OR remedy" which returned 113 hits minus duplicates (as of July, 2020).

Table S1: Traditional medicinal uses of molluscs for respiratory related conditions used in ancient and modern cultures around the world appearing in ethnomedical texts and peer-reviewed articles.

Class<br>Family<br>*Species*	Region	Traditional remedy (rem) or local species (sp) name	Part used	Respiratory ailment/disease used to treat[1]	Preparation and application	Reference
**Bivalvia**						
Megalobulimidae						
Megalobulimus oblongus	South America	NA	Whole animal	Asthma	NA	[1]
Mytilidae						
Mytilus unguiculatus[2]	Korea	Honghap (sp)	Whole animal	Fever[3]	Decoction, taken orally	[2]
Ostreidae						
Crassostrea rhizophorae	South America	NA	Flesh and shell	Pneumonia; flu; tuberculosis; cancer[4]	NA	[1]
Ostrea edulis	Europe	Calcarea carbonica (homeopathic rem)	Shell (inner layer)	Catarrh; laryngismus; asthma; hypersensitivity (asthma and ENT allergies); upper respiratory tract infections in children	One pill or five drops of the remedy every hr for intense symptoms, or 4 hr for milder ones	[3-5]
<u>Pteriidae</u>						
Pinctada margaritifera (and other Pinctada sp.)	India	Mukta bhasma (rem)	Pearl (ash)	Cough; asthma; phthisis[5]; low fevers[6]	Pearls boiled in juices of *Sesbania* sp. leaves and flowers then calcined and powdered; transferred to a lemon and stored in paddy before being heated and ashed; ash given twice daily w honey; can be combined w other medicines	[6]
Teredinidae						
Neoteredo reynei	South America	NA	NA	Tuberculosis	NA	[1]
Lyrodus pedicellatus[7]	South America	NA	NA	Tuberculosis	NA	[1]
Veneridae						
Anomalocardia brasiliana[8]	South America	NA	Flesh and shell	Asthma; flu	NA	[1]
**Cephalopoda**						
Loligninidae						
Loligo sp.	South America (north/north-eastern Brazil)	Lula (sp)	Shell	Asthma	NA	[7]
Loliginidae, <u>Octopodidae</u>						
Loligo sp. *and Octopus* sp.	South America (Brazil)	NA	Shell and flesh	Asthma	Tea of toasted cuttlefish bones or octopi arms	[8]
<u>Octopodidae</u>						
Octopus vulgaris	Ancient Greece	Polypus (sp)	Flesh	Heavy nasal congestion w fever; infectious diseases; low immunity[9]	Flesh boiled or roasted	[9]
Sepiidae						
Sepia officinalis	Europe	Sepia (homeopathic rem)	Ink	ENT and pulmonary allergies in children and adults	NA; usually in combination w other drugs	[5]
S. officinalis	India	NA	Shell	Ear pain; inflammation; otorrhoea	Powdered, used as a dusting powder; fine scraping of bone added to sweet oil or sesame oil and instilled into ears	[6]
S. officinalis	Ancient Greece (~800 BC)	Sepia (sp)	Flesh	Low immunity	Flesh boiled	[9]
**Gastropoda**						
Achatinidae						

Abbreviations: NA: data not available; w: with; ENT: ear, nose and throat.

[1] Some traditional remedies have numerous applications and may be used to treat ailments/diseases other than those relating to the respiratory system; please refer to references for additional uses

[2] Listed as *Mytilus coruscus* (Gould 1861)

[3] Not specific to respiratory disease, although a common symptom of respiratory infection

[4] Not specific to respiratory cancer

[5] Also known as pulmonary tuberculosis

[6] Not specific to respiratory disease, although a common symptom of respiratory infection

[7] Listed as *Teredo pedicellata* (Quatrefages 1849)

[8] Listed as *Anomalocardia brasiliana* (Gmelin 1791)

[9] Generally written as "strengthens body's immune system"; not specific to respiratory disease, however may assist with prevention and reduced duration of infection; included for the purpose of this review to capture the range of applications for traditional molluscan medicines

Limicolaria aurora	Nigeria	Okoso (sp)	Mucus	Cough	Heat slightly; fluid taken orally from the shell 2-3 times daily	[10]
Ampullariidae						
Lanistes ovum	Nigeria	Okpaamu (sp)	Mucus	Cough	Puncture the flesh to obtain fluid; take 1 tablespoonful twice daily	[10]
Pomacea lineata	South America	Arua (sp)	Ova (eggs)	Asthma	NA	[10]
Plia globosa	Nagaland (northern India)	Different sp. names among tribes[10]	Flesh	Asthma; tuberculosis	Flesh cooked and eaten	[11]
Aplysiidae						
Aplysia depilans	Middle East (Middle ages)	NA	NA	Dyspnoea; dry cough; haemoptysis	NA	[12]
Cassidae						
Cassis tuberosa	South America	NA	NA	Asthma	NA	[1]
Cypraeidae						
Monetaria moneta[11]	India	Cowrie bhasma (rem), Kapardika (sp.)	Shell	Asthma; cough; ear ache; used as expectorant in chronic bronchitis	Calx (powder) prepared from shell taken orally; instillation of ash with lemon juice for ear ache	[6]
Charoniidae						
Charonia tritonis	Ancient Greece	Keryx (sp)	Flesh and shell	Ear pain; low immunity; parotid gland swelling	Flesh applied directly for ear pain; flesh taken internally to strengthen immune system; ashes of burned shell for gland swelling[12]	[9]
Limacidae						
Limax sp.	China (since 1596)	NA	Body	Wheeze, pharyngitis, asthma	NA	[13]
Helicidae						
Helix pomatia (and other large terrestrial snail sp.)	Europe (particularly France) 1800's – modern day	Snail syrup (rem) later Helicine (or pertussidine or pomaticine) (rem)	Mucus (or whole)	Cough and cold; whooping cough; chronic bronchitis; tonsillitis; pharyngitis; hoarseness; sore throat; influenza; croup; nervous cough in children; pneumonia and pulmonary phthisis; anthrax; acute and chronic chest ailments; weakness; cough associated w measles, fever and other inflammatory conditions; scrofula	Helicine- transparent yellow oil extract taken orally; snail formulations also include snail sugar, tablets, syrup, paste, chocolate and ointment; snail water for TB (between one and six ounces); snail paste with donkey milk	[14] and references therein
Littorinidae						
Littorina littorea	Nigeria	Esiemu (sp)	Mucus	Inner ear[13]/sinus inflammation	Crack the shell at the tail of the live animal to obtain fluid; instill 1-3 drops in ear twice daily	[10]
Littoraria angulifera[14]	South America	Mela-pau (sp)	Flesh	Chesty cough; shortness of breath	NA	[1]
Lymnaeidae						
Lymnaea sp.	North-eastern India	Chengkawl (sp)	Flesh	Measles	Boiled in water, then flesh is removed and eaten	[15]
Muricidae						
Unidentified sp.	Ancient southern India	Nakhi (rem)	Opercula	Remove phlegm; destroy poison	Used in incense and medicinal oil, heated in clarified butter or cooked w honey	[16] [17]
Chicoreus virgineus	Medieval Eastern Mediterranean Genizah (Cairo)	Blatta (rem)	Opercula	Tumors; eye and ear diseases	NA	[16] [18]
Hexaplex trunculus, *Bolinus brandaris* and *Stramonita haemastoma*[15]	Ancient Greece	Banded dye murex, spiny dye murex, rock-shell (sp)	Shell and operculum	Parotid gland swelling and hearing loss; inflammatory conditions	Operculum pulverised and mixed w oil and vinegar	[9]
Onchidiidae						
Onchidium reevesii	China	NA	Body	Asthma	NA	[19]
Turbinellidae						

[10] Jemna, Noula, Achokibo, Mongkoum, Kyakyiro, Shunyaknaloong, Nuyushu, Chakuthe, Yebo, Khapjo
[11] Listed as *Cypraea moneta* (Linnaeus 1758)
[12] Unclear whether applied directly or taken internally
[13] Listed a treatment for inner ear inflammation, though more likely a treatment for middle ear inflammation/infection relating to topical application to tympanic membrane
[14] Listed as *Littorina angulifera* (Lamarck 1822)
[15] Listed as *Thais haemastoma* (Linnaeus 1767)

Turbinella rapa or *T. pyrum*[16]	India	Sankha (sp), Sankha bhasma (rem)	Shell (ash), flesh	Asthma; tuberculosis and tumours (flesh); excessive mucus, sore throat, earache, high fevers and cough (shell powder)	Shells incinerated to powder (calcined); prepared into pills, salves and pastes	[6]
Veronicellidae						
Unidentified sp.	South America (north/north-eastern Brazil)	Lesma (sp)	Whole animal	Sore throat	NA	[20]
Viviparidae						
Filopaludina sp.	North Bihar, India	NA	Foot	Asthma	Soup prepared from foot and taken orally	[21]

[16] Listed as *Xancus pyrum* (Linnaeus 1767)

Table S2: Traditional Chinese medicines (TCMs) derived from molluscs relevant to the treatment of respiratory disease listed in the Chinese *Marine Materia Medica* [22] presented directly as translated by L. Liu.

**Class**<br>Family<br>*Species*[1]	**Traditional remedy name**	**Part used**	**Respiratory ailment/disease used to treat**	**Preparation and application**
**Bivalvia**				
Anomiidae, Placunidae				
Placuna placenta, P. ephippium, Enigmonia aenigmatica, Anomia chinensis	Hai Yue	Flesh and shell	Measles; retention of sputum[2]	Boil flesh and eat (30-60 g each time); grind powder for infusion and ingest
Arcidae				
Anadara inaequivalvis	Bi Na Han	Shell	Tuberculosis of lymph nodes[3]; cough	Decoct the crushed shell and ingest (15-50 g each time)
Arcidae, Noetiidae, Limidae				
17 sp.	Wa Leng Zi	Shell	Phlegm in hypochondrium; chronic cough; scrofula	Decoct and ingest (9-15 g each time); grind into powder for infusion (1.5-3 g); ustulate (scorch) the shell, grind into powder and apply externally
Donacidae				
Donax faba	Dou Fu Ge Ke	Shell	Tuberculosis of lymph nodes; cough and phlegm	Decoct and ingest (15-50 g each time)
D. faba	Fu Ge Ke	Shell	Tuberculosis of lymph nodes	Ustulate the shell, decoct and ingest.
Glauconomidae				
Glauconome chinensis	Lu Lang Ke	Shell	Tuberculosis of lymph nodes	Decoct and ingest (15-50 g each time)
Lucinidae, Carditidae, Semelidae, Glossidae				
9 sp.	Man Yue Ge Ke	Shell	Tuberculosis of lymph nodes; cough and phlegm	Grind into powder and ingest, or decoct and ingest (15-50 g)
Mactridae, Astartidae				
Mactra quadrangularis (or *Astarte borealis*)[4], *Lutraria rhynchaena*[5], *M. grandis*[6], *M. inaequalis*	Ge Li/Ge Li Fen	Flesh and shell	Retention of phlegm; asthmatic cough; scrofula; edema[7]	Decoct crushed shell and ingest (50–100 g each time), grind into powder and ingest or apply externally
Mactridae, Psammobiidae				
Mactra antiquata, Haitula diphos	Xi Shi She	Shell	Tuberculosis of lymph nodes; conjunctive congestion with swelling and pain[8]; pharynalgia	Ustulate the shell, decoct and ingest.
Myidae				
Mya arenaria	Sha Hai Lang	Shell	Tuberculosis of lymph nodes	Decoct and ingest (15-30 g each time)
Ostreidae, Grypaeidae				
12 sp.	Mu Li	Flesh and shell	Tuberculosis of lymph nodes; cancer and neoadjuvant treatment[9]; scrofula	Smash flesh and apply externally
Pharidae				
Sinonovacula constricta	Cheng Ke	Shell	Sore throat	Ingest calcined and pulverised powder (3-6 g) with water
Psammobiidae				
Gari maculosa, Gari radiata, G. elongata[10], *Asaphis violascens, Nuttallia obscurata*[11], *S. virescens, S. chinensis*	Zi Yun Ge	Shell	Tuberculosis of lymph nodes; cough and phlegm	Decoct and ingest (15-50 g each time)
Pteriidae				
Isognomon isognomum, I. perna, I. nucleus, I. legumen	Qian Ge	Adductor muscle	High fever and convulsion (children)[12]; tuberculosis of lymph nodes; cough and phlegm	Decoct and ingest (15-25 g each time) or grind into powder and ingest

[1] For the purpose of this table, the complete list of species is provided where the remedy comprises ≤8 sp. For those remedies comprising >8 sp, a comprehensive list of species is available upon request of the authors
[2] Retention of sputum, retention of phlegm and retention of fluid in chest also known as pulmonary congestion
[3] The bacteria that cause pulmonary tuberculosis can cause symptoms outside the lungs; "tuberculosis of lymph nodes" is one of the most common extrapulmonary manifestations of tuberculosis, whereas "scrofula" (or cervical lymphadenopathy) refers specifically to tuberculosis of the lymph nodes in the neck (Jha et al 2001). Although these definitions may be interchangeable, the information presented remains directly as translated (Liu, pers. com).
[4] Listed as *M. veneriformis* (Reeve 1854/Wood 1828)
[5] Listed as *L. australis* (Reeve 1854)
[6] Listed as *M. mera* (Reeve 1854)
[7] Not specific to respiratory system though included as lung edema may accompany other ailments listed
[8] Typically a secondary manifestation of respiratory infection
[9] Not specific to cancer of the respiratory system
[10] Listed as *Sanguinolaria elongata,* (Lamarck 1818)
[11] Listed as *S. olivacea* (Jay 1857)
[12] Not specific to respiratory disease, however may accompany respiratory infection

Pinctada imbricata[13], *P. margaritifera, P. maxima, P. chemnitzi, Electroma alacorvi*[14], *Pteria heteroptera*[15], *P. penguin*	Zhen Zhu/Zhen Zhu Mu	Pearl/shell hypostracum and prismatic layer	Pharyngitis; retention of fluid in chest; cough and regurgitation; conjunctive congestion; bleeding from five aperture or subcutaneous tissue (e.g. eye, ear, nose, teeth, tongue)[16]	Grind pearl into powder and ingest (0.3-1 g each time) or apply externally; grind shell into powder and apply externally
Solenidae, Pharidae, Solecurtidae				
13 sp.	Ma Dao	Shell	Sore throat; retention of phlegm and fluid	Decoct and ingest (5-15 g)
Tellinidae				
15 sp.	Ying Ge Ke	Shell	Tuberculosis of lymph nodes; cough and phlegm	Ustulate the shell, grind into powder and ingest
Veneridae				
10 sp.	Hai Ge Ke	Shell	Asthma; scrofula	Decoct and ingest (10-25 g each time); grind into powder and apply externally
Ruditapes philippinarum, R. variegatus[17] (or *Venerupis aspera*)	Ge Zai	Flesh and shell	Asthmatic cough	Boil and eat flesh, decoct and ingest; or ustulate (scorch) the shell, grind into powder and apply externally
Mercenaria mercenaria	Ying Ke Ge	Shell	Dyspnoea with cough; scrofula	Decoct and ingest (10-15 g each time)
Paphia amabilis	He Ai Ba Fei Ge	Shell	Phlegmatic heat and cough; tuberculosis of lymph nodes; pain in sternum[18]	Decoct and ingest (10-15 g each time)
Mactra chinensis	Ke	Shell	Cough and phlegm	Decoct the crushed shell and ingest (20-50 g each time), grind into powder and apply externally
13 sp.	Wen Ge Rou	Flesh	Cough with dyspnoea and chest stuffiness; scrofula	Boil and eat flesh (30-60 g)
Paphia lirata	Wen Ban Ba Fei Ge	Shell	Cough and phlegm; chronic tracheitis	Decoct and ingest (10-15 g)
10 sp.	Ge Ke	Shell	Phlegmatic heat and cough; scrofula; pain in sternum	Decoct the crushed shell and ingest (10–15 g each time) or grind into powder and apply externally
Meretrix lamarckii	Fu Wen Ge/Fu Wen Ge Ke	Shell and flesh	Scrofula; cough	Decoct and ingest (6-15 g each time) or grind into powder and apply externally
Protapes gallus	Ju Chi Ba Fei Ge	Shell	Scrofula	Decoct and ingest (10-15 g each time)
**Cephalopoda**				
Sepiidae				
9 sp.	Hai Piao Xiao	Cuttlebone	Bleeding from five aperture or subcutaneous tissue (e.g. eye, ear, nose, teeth, tongue)	Decoct and ingest (10-30 g)
**Gastropoda**				
Aplysiidae				
Bursatella leachii, Aplysia argus, A. kurodai, A. dactylomela, Dolabella auricularia	Hai Fen	Egg masses	Xeropulmonary cough[19]; dyspnoea with cough; tuberculosis; scrofula; tuberculosis of lymph nodes; epistaxis[20]	Decoct and ingest (30-60 g each time)
Buccinidae, Nassariidae				
Neptunea cumingii, Phos senticosus	Xiang Luo	Shell	Tuberculosis of lymph nodes	Decoct and ingest (15-25 g each time) or apply externally
Conidae				
23 sp.	Yu Luo Ke	Shell	Tuberculosis of lymph nodes	Decoct and ingest (15-25 g each time) or ustulate the shell, grind into powder and ingest (3–6 g)
Cypraeidae				
11 sp.	Bai Bei	Shell	Acute and chronic sinusitis	Decoct and ingest (5-15 g each time)
10 sp.	Zi Bei	Shell	Children with fever; conjunctive congestion with swelling and pain; acute and chronic sinusitis; measles; heat toxicity; headache[21]	Decoct and ingest (15-25 g each time)
Lyncina carneola	Rou Se Bao Bei	Shell	Acute and chronic sinusitis; high fever	Grind into powder, decoct and ingest (5-15 g each time)

[13] Listed as *Pinctada fucata martensii* (Dunker 1880)
[14] Listed as *Electroma ovata* (Quoy & Gaimard 1835)
[15] Listed as *Pteria brevialata* (Dunker 1872)
[16] Unclear whether specific to respiratory disease; may be caused by secondary infection
[17] Listed as *R. variegata* [sic]
[18] Not specific to respiratory disease, however may be symptomatic of inflammation or persistent cough
[19] Also known as dry cough
[20] Arises from burst blood vessels in nose; may be due to allergies, sinus and nasal infections, coughing and sneezing, as well as non-respiratory ailments or trauma
[21] Heat toxicity (?) and headache may accompany other listed respiratory ailments

Naria erosa[22]	Yan Qiu Bei	Shell	Phlegm; tuberculosis of lymph nodes; conjunctive congestion with swelling and pain; fullness sensation in chest and shortness of breath	Grind into powder, decoct and ingest (5-15 g each time)
Fasciolariidae				
Filifusus filamentosus[23], *Pleuroploca trapezium*	Xi Lei Luo Ke	Shell	Phlegm; cough	Decoct and ingest (15-30 g each time)
Ficidae				
Ficus gracilis, F. subintermedia[24], *F. ficus*	Pi Ba Luo Ke	Shell	Children with fever; night sweating; chronic tracheitis; feverish sensation in chest	Grind into powder, decoct and ingest (15-25 g each time)
Haliotidae				
9 sp.	Bao Yu	Flesh	Cough	Boil and eat the flesh or decoct and ingest (fresh 6-9 g each time or sun-dried 15–50 g each time)
Haminoeidae				
Bullacta caurina[25]	Tu Tie	Flesh	Pharyngitis; tuberculosis; cough	Boil flesh and eat moderate amount (about 30 – 200 g each time)
Harpidae, Cassidae				
Morum cancellatum, Phalium areola, Cassis cornuta, Semicassis bisulcata, P. flammiferum, P. glaucum	Guan Luo Ke	Shell	Tuberculosis of lymph nodes	Grind into powder, decoct and ingest (15-50 g each time)
Melongenidae				
Hemifusus tuba, Brunneifusus ternatanus[26]	Jiao Luo/Jiao Luo Yan	Flesh/shell	Otitis media	Grind into powder, decoct and ingest (5-15 g each time); apply flesh externally
Muricidae				
Rapana bezoar, R. rapiformis, R. venosa	Hai Luo/Hai Luo Ke	Flesh/shell	Chest and abdomen heat and pain; scrofula	Boil flesh and eat moderate amount (30-60 g); decoct the shell and ingest (15-30 g); used as medicinal powder- ustulate the shell, grind into powder, mix with sesame oil and apply externally.
Indothais gradata, Reishia luteostoma	La Luo	Shell	Clear heat[27]; scrofula; phlegm and cough	Decoct the crushed shell and ingest (15-25 g)
Murex aduncospinosus, M. pecten[28], *Vokesimurex rectirostris, M. ternispina, M. trapa, Nassa francolina*	Gu Luo	Shell	Clear heat; otitis media	Decoct the shell and ingest; ustulate the shell, grind into powder and apply externally.
12 sp.	Liao Luo	Shell	Clear heat; scrofula	Decoct the shell (15-50 g) and ingest; ustulate the shell, grind into powder and apply externally; used for making pills or medicinal powder
Chicoreus ramosus	Ji Luo	Shell	Clear heat; scrofula	Decoct the crushed shell and ingest; or ustulate the shell, grind into powder and apply externally
Chicoreus brunneus	He Ji Luo	Shell	Scrofula	Decoct the shell and ingest
Rapana rapiformis	Hong Luo	Shell	Scrofula	Decoct the shell (15-25 g) and ingest
Nacellidae				
Cellana toreuma, C. testudinaria	Jia Qi	Shell	Conjunctive congestion with swelling and pain; scrofula; phlegm	Decoct and ingest (10-15 g each time)
Naticidae				
14 sp.	Yu Luo Ke	Shell	Tuberculosis of lymph nodes	Decoct and ingest (15-50 g each time)
Ranellidae, Personidae				
10 sp.	Qian Xian Luo Ke	Shell	Tuberculosis of lymph nodes	Grind into powder, decoct and ingest (15-50 g each time)
Strombidae				
10 sp.	Feng Luo Ke	Shell	Tuberculosis of lymph nodes	Decoct and ingest (15-25 g each time)
Tonnidae				
Malea pomum, Tonna chinensis[29], *T. galea, T. perdix, T. sulcosa*	Chun Luo Ke	Shell	Tuberculosis of lymph nodes; hypertension	Grind into powder or ustulate the shell, decoct and ingest (15-30 g each time)
Turritellidae				
Neohaustator fortilirata[30], *Turritella terebra, T. bacillum*	Zhui Luo Yan	Operculum	Conjunctive congestion with swelling and pain	Decoct and ingest (15-30 g each time)
**Polyplacophora**				

[22] Listed as *Erosaria erosa* (Linnaeus 1758)
[23] Listed as *Pleuroploca filamentosa* (Roding 1798)
[24] Listed as *F. ficus (subintermedius)*
[25] Listed as *Bullacta exarata* (Philippi 1849)
[26] Listed as *H. ternatanus* (Gmeline 1791)
[27] Unclear whether "clear heat" refers to fever or otherwise; medicine use to "clear heat" only included in this review if also used for respiratory diseases/symptoms
[28] Listed as *M. pecten pecten* (Lightfoot 1786)
[29] Also listed as *T. chinensis (magnifica)* (G. B. Sowerby III 1904)
[30] Listed as *Turritella fortilirata* (G. B. Sowerby III 1914)

Acanthochitonidae				
Acanthochiton rubrolineata	Hai Shi Bie	Whole	Asthma; pulmonary tuberculosis; bronchitis	Grind into powder and prepared as infusion, or made into capsule or tablet for oral administration (2-6 g each time)
Ischnochitonidae				
Ischnochiton hakodadensis, Lepidozona coreanica, I. boninensis, I. comptus	Cuo Shi Bie	Whole	Asthma; pulmonary tuberculosis; bronchitis	Grind into powder and infusion for oral administration (1-3 g each time)

Table S3: Human clinical trials and *in vivo* animal models using molluscan hemocyanins as vaccine adjuvants/conjugates for respiratory disease.

**Mollusc class**<br>Family	**Model and study design**[1]	**Main findings**	**Effective concentrations**[2]	**Reference**
**Gastropoda**				
Fissuerllidae	Clinical trial involving 13 small-cell lung cancer (SCLC) patients (10 completed) receiving 5-6 s.c. immunisations w Fuc-GM1 (SCLC ganglioside) 30 µg conjugated to KLH (696:1) w QS-21 adjuvant	Fuc-GM1 immunogenicity enhanced by conjugation to KLH and mixture w QS-21 adjuvant; increased IgM and IgG Ab titers despite prior chemotherapy treatment; well-tolerated and non-toxic; antisera antibodies from 6/10 patients bound to H146 (human lung carcinoma) cells	30 µg antigen-KLH conjugate (696:1)	[23][3]
	Randomised, double-blind, placebo-controlled clinical trial involving 60 patients w cypress pollen allergy (allergic rhinitis); 3x i.m. immunisations w 250 µg peptide-KLH conjugate (1:2) vaccine (or sucrose placebo) at 28-d intervals + booster; challenged w lyophilised *Cupressus sempervirens* pollen i.n.	Nasal challenge threshold dose and skin sensitivity not sig different between vac and placebo; IgE lower but not statistically; IgG sig elevated; anti-KLH antibodies not detectable; overall positive Ab response, however the vaccine was not effective against cypress pollen allergy symptoms	250 µg antigen-KLH conjugate (1:2)	[24]
	Double blind, randomised, phase III multicentre clinical trial of Sialyl-TN (STn)-KLH vaccine for metastatic breast cancer involving 1028 women, 38% w metastatic lung cancer (~350 patients) immunised i.m. w STn-KLH conjugate (9%) (or 100 µg KLH controls)[4] (monthly-quarterly treatments for up to 50 mo)	High anti-OSM and anti-STN Ab titers in STn-KLH group, none in KLH control group; anti-KLH Ab responses present in both groups, but at lower median levels in the STn-KLH group- higher response to the hapten (STn) than to the carrier (KLH) (appropriately designed vaccine); higher IgM than IgG; no sig dif in median time to progression or survival time between groups; well tolerated	100 µg antigen-KLH conjugate (9%)	[25]
	Combination cancer therapy clinical safety/feasibility study involving 26 patients w advanced treatment refractory cancer (including 4 lung); immature dendritic cells (iDC) mixed w lymphocyte conditioned media + 1 mg KLH injected into metastatic lesions (i.t.t.) followed by i.v. activated T-cells and radiation treatment	Increase in anti-KLH Abs after 36 d (p<0.05); frequency of KLH immunity sig. higher in the complete response group (58.3%) compared to recurrent and progressive disease groups; KLH immunity had better overall survival; no toxicity observed; DCs can acquire co-injected antigens and generate an adaptive immune response	1 mg KLH	[26]
	Haemophilus influenzae (NTHi) immunisation model using NZ white rabbits (n=2/group) immunised 3x s.c. w one of 3x synthetic NTHi lipo-oligosaccharide (LOS)-related peptides conjugated to KLH in CFA/IFA (or KLH alone)[5]	All 3 peptide-KLH conjugates elicited high anti-LOS IgM, IgG Ab titers; KLH alone showed lower binding reactivity and Ab titers; other results not specific to KLH treatment	500 µg KLH	[27]
	Acute pneumonia immunisation model using mice (n=20/experiment) immunised w one of 10x *P. aeruginosa*-derived peptides conjugated to KLH; peptide-KLH w alum administered s.c. twice (+2x boosters); KLH or peptide alone controls (same concs)	Higher anti-peptide IgG Ab titers and opsonic/phagocytic activity w conjugates compared to peptides or KLH alone (p<0.05); KLH alone showed higher Ab titers than some conjugate treatments and increased Ab and opsonic/phagocytic response though not sig dif to naïve mouse sera	150 µg KLH alone; 150 µg antigen-KLH (25:20)	[28]
	Acute pneumonia immunisation-infection model using mice (n=30-50/group) immunised w 50 µg of one of 4x peptide-KLH (or -BSA) conjugates w CT[6] administered 2x i.n., before *P. aeruginosa* pneumonia challenge; carrier only controls- KLH followed by BSA	Higher anti-peptide IgG, IgA Ab titers with conjugation; 47-78% survival depending on protein; survival with conjugates (up to 78%) higher than KLH/BSA alone (46%) (p<0.04)	150 µg KLH alone; 150 µg antigen-KLH (25:20)	[29]
	Influenza immunisation-infection model using C57BL/6 or Balb/c mice (n=5-15/group) immunised i.m. w 5 µg influenza hemagglutinin (HA) stem domain (stemHA), stemHA-KLH or full-length HA w CFA/IFA; challenged i.n. w 50 µL $TCID_{50}$ influenza virus (clinical strains)	stemHA-KLH or full-length HA immunisation resulted in high Ab titers and strong recruitment of stem-specific B cells (p<0.01 compared to stem HA alone); poor immune responses to stem HA- relieved by covalent coupling to KLH; can confer broad protection to different strains	5 µg antigen-KLH (ratio NA)	[30]
	Allergic asthma immunotherapy model using Babl/c mice (n=5/group) sensitised i.p. to recombinant grass pollen antigen (rPh1) then challenged i.n. to aerosol rPh1; 6x s.c. immunisations w Ph1 mimotope coupled to KLH, KLH alone, or rPh1 in PBS and re-challenged w aerosol rPh1	Ph1 mimotope-KLH and rPh1 decreased inflammatory cell infiltration, mucus secretion, Th2 cytokine expression (p<0.05) compared to mimotope or KLH alone; KLH alone caused higher BALF/spleen eosinophil and goblet cell counts and cytokine (IL-4, IL-5, IFN-y) levels compared to PBS treatment (p<0.05); anti-Ph1 IgG1, IgE Ab titers not sig dif between treatments	antigen-KLH (~1:50); KLH alone (10 µg/100 µL)	[31]
	Acute pneumonia/*P. aeruginosa* (Pa) infection model using CD-1 mice (n=13-14/group) administered i.n. FpvA (Pa membrane receptor) conjugated to KLH, or FpvA only or whole-cell Pa vaccine) (or FpvA, PBS or KLH alone) w curdlan adjuvant, booster at 21 d, i.n. bacterial challenge at day 34 w *P. aeruginosa* PAO1 strain	FpvA-KLH higher anti-Pa/anti-FpvA IgG, IgM, IgA Ab titers, >CD11+ dendritic cells and memory CD4+ cells, <bacterial burden and lung edema, >Ab response to both clinical CF Pa isolates and laboratory strains (p=0.0001-0.05 compared to PBS or FpvA only); comparable activity to whole cell vaccine; induced a humoral and IL-17-type cellular immune response	30 µg antigen-KLH (1:1)	[32]

Abbreviations: Ab: antibody; Hc: hemocyanin; w: with; KLH: keyhole limpet hemocyanin; CFA: Complete Freund's Adjuvant; IFA: Incomplete Freund's Adjuvant; BSA: Bovine serum albumin; BALF: bronchoalveolar lavage fluid.

[1] Administration routes: i.p.- intraperitoneal, i.n.- intranasal, s.c.- subcutaneous, i.v.- intravenous, i.m.- intramuscular, i.t.- intratracheal, i.c.- intracutaneous, i.b.- intrabullar

[2] All hemocyanins derived from hemolymph, and all laboratory grade unless indicated as experimentally purified (*).

[3] Dickler et al. (1999), and several similar studies (e.g. Babatz, 2006; Krug et al., 2004a, 2004b, 2012), are examples of the use of KLH in conjugate preparations to improve vaccine immunogenicity, however not provide specific information on KLH bioactivity and are therefore not comprehensively reviewed in this table

[4] Initial four immunisations with Enhanzyn™ adjuvant, no adjuvant thereafter

[5] Also included a passive immunisation-infection model using Balb/c mice (n=10/group) immunised with 40 or 80% rabbit antisera before NTHi challenge, though did not used antisera from control rabbit treated with KLH only

[6] Cholera toxin subunit used as stimulatory adjuvant, known to activate mast cells

	Tuberculosis immunisation model using Swiss Webster outbred mice (n=5-15/group) immunised w *M. tuberculosis* cell wall peptide (LAM) mimotopes (e.g. HS, SG, P1 peptides) conjugated to KLH (s.c.) or AcMNPV baculovirus (i.n.) (or KLH or PBS alone) + 3x boosters[7]	Only mice immunised w HCpeptide-KLH generated Abs that cross-reacted w LAM (p<0.0001); peptide-KLH higher anti-LAM IgG Ab titers as more available cysteine residues for conjugation + immune stimulation	10 μg antigen-KLH (1:1)	[33]
	Respiratory syncytial virus (RSV) infection-immunisation-pulmonary eosinophilia model using Balb/c mice (n=5/group) immunised i.m. 2x w RSV Gpeptides conjugated to KLH or purified Gprotein (no KLH) (or PBS or KLH alone) w QS-21 adjuvant, challenged w RSV i.n. 2 wk after	KLH effective carrier of smaller Gprotein functional peptide; higher BALF eosinophil counts w Gprotein and peptide15-KLH compared to KLH or PBS alone (p<0.05; no difference between KLH and PBS)	250 μg antigen-KLH (50–80:1)	[34]
	Pseudomonas aeruginosa immunisation-infection model using C3H-HeN mice (n=5/group) and NZ white rabbit (n=1) immunised s.c. w *P. aeruginosa* polysaccharide (MEP) w KLH; mice: MEP, MEP + KLH mixture, or MEP-KLH conjugate (no adjuvant) x3 s.c. vaccinations; rabbit: MEP-KLH w CFA/IFA x2 s.c. vaccinations + 4x i.v. boosters	Mixture induced significant IgM but failed to induce IgG (T-cell-independent response); conjugate elicited both IgM and IgG and higher antisera opsonophagocytic killing (p<0.01) correlating w high lung clearance; both mixture and conjugate better result than MEP alone	1 μg antigen-KLH conjugate; >10 μg antigen + KLH mixture	[35]
	Pseudemonas aeruginosa and *Burkholderia cepacia* immunisation-infection model using Sprague-Dawley rats (n=9/group) immunised i.m. w pep15-KLH, pep42-KLH, or KLH in CFA + 2x boosters; bacterial challenge	High anti-Pa Ab titers after 3rd immunisation; BALF inflammatory cell counts and lung damage sig. reduced (50-70%) in peptide-KLH groups compared to KLH alone; pep15-KLH most effective; no sig dif in lung bacterial clearance among groups	antigen-KLH conjugate (5/7:1)	[36]
	Non-typeable *Haemophilus influenzae* (NtHi) immunisation-infection-clearance models using DA and Sprague Dawley rats (n=5/group) immunised mucosally (i.t.) w recombinant lipoprotein peptides (LP) conjugated to KLH, BSA or full-LP protein, and combinations thereof; challenge using 4x NtHi viral strains i.t. or i.b.[8]	Anti-LP IgG, IgA Ab titers were not sig dif. between KLH conjugates and other groups, peptide-full-length conjugate and mixtures resulted in highest titers; KLH conjugation enhanced bacterial clearance compared to BSA conjugates, KLH/BSA alone and naïve mice (P<0.05)	10-20 μg antigen-KLH (NA ratio)	[37]
Helicidae	Influenza (H3N2) immunisation model using Balb/c mice (n=5-8/group) immunised i.p. w HpH (or PBS) no adjuvants or peptides; other groups immunised w influenza peptide (IP), IP w CFA, IP w alum, IP w HpH, or IP w alum; boosted 2 x; separate tetanus (TT) toxoid model	IP+HpH produced high anti-IP Abs (p<0.01 compared to control), though IP w alum most effective (p<0.005); higher cytokine levels w HpH+IP compared to alum+IP (p<0.005); high anti-HpH Abs at all doses (p<0.005); IP+KLH stronger cytotoxicity against infected cells compared to other groups (p<0.0005); strong B and T cell proliferation	100 μg HpH*; 50 μg antigen + 100 μg HpH* mixture	[38]
Muricidae[9], Fissuerllidae	Influenza immunisation model using Balb/c mice (n=6-8/group) immunised i.p. w commercial flu vaccine (influenza hemagglutinin 15 μg) mixed w RvH or KLH (100 μg) or vaccine in CFA + 2x boosters (or PBS or RvH alone controls)	Anti-Vac IgM Ab treatments > controls (p<0.0005); IgG significantly higher in the Vac+KLH treated group (p<0.005) and Vac+RvH (p<0.01 compared to Vac, Vac+CFA and PBS); lower cytokine levels (IL4 and IFN-y) w Vac+KLH and Vac+RtH (p<0.05) (Th1/Th2 immune response); Vac+RvH highest cytotoxicity against infected cells (p<0.01 compared to control)	15 μg antigen + 100 μg RvH*/KLH mixture	[39]

[7] Also included a separate infection model (same vaccination protocol) for challenge with aerosol *M. tuberculosis* (Erdman strain), though did not use KLH
[8] Also included an NtHi and adenovirus immunisation-infection model using Chinchillas which did not use KLH
[9] *Rapana venosa* originally listed as *R. thomasiana* (Crosse 1861)

Table S4: *In vivo* animal models and human studies using molluscan hemocyanins as a model antigen for research investigating respiratory diseases.

Mollusc class<br>Family	Study type and design[1]	Main findings	Effective concentrations[2]	Reference
**Gastropoda**				
Fissuerllidae	Model of acute airway inflammation and antioxidant response to nitrated antigens using Balb/c mice (n=3-4/group) sensitised w OVA + alum i.p., challenged i.n. w 50 µg nitrated OVA (nOVA) or nitrated KLH (nKLH) in PBS, or OVA, KLH or PBS alone (controls); challenged w MCh for lung function test	KLH or nKLH decreased glutathione oxidation and superoxide dismutase gene expression ($p<0.05$); no lung function abnormalities w KLH or nKLH challenge; increased inflammatory cells (particularly lymphocytes and macrophages) in KLH and nKLH groups ($p<0.001$); OVA/nOVA produced stronger effects than KLH/nKLH	50 µg	[40]
	Pulmonary immune response model investigating the role of alveolar macrophages (AM) using (C3D2) F1 mice (n=2-4/group) challenged w 80 µg trinitrophenyl-KLH in PBS i.t. w or w/out 10 µg liposome Cl_2MDP pre-treatment (for AM elimination)	Loss of AMs caused enhanced immune responses- increased antigen presenting cells and prolonged response, increased IgG, IgA and IgE Abs; no increase in response after antigen dose increased >800 µg; AMs have a suppressive role limiting possible damage caused by severe responses in lung tissue	80 µg antigen-KLH	[41]
	Allergic asthma model investigating the role of IL-1 family cytokines using C129S7(4Get) mice (n=4/group) sensitised i.n. to 100 µg aerosolised KLH (or short ragweed extract) w or w/out 100 ng IL-33 twice before 3x i.n. challenge exposures to KLH; main component of study uses OVA challenge	IL-33+KLH increased IL-4, IL-5, IL-13 production, BALF inflammatory cell counts and IL-4eGFP expression by CD4+ cells ($p<0.01$); KLH alone comparable to PBS; IL-33 facilitates sensitisation to antigens producing asthma-like lung changes upon re-exposure; KLH does not induce memory-type immune responses	100 µg	[42]
	Allergic asthma models (x3) using BDF1 mice (n=4-6/group) immunised i.n. w 0.05-5 µg (exp 1) or 0.5-25 µg (exp 2) aerosolised sensitisation proteins including KLH, OVA, subtilin (SUB), beta-lactoglobulin (BLG) and mouse serum albumin (MSA) (saline or naiive mice controls); challenged 29 d later w MCh (exp 1) or sensitisation proteins (exp 2); passive immunisation model using naiive mice immunised w antisera then challenged w proteins i.v. (exp 3)	KLH induced a dose-dependent increase in BALF total cells, eosinophil, macrophage and counts ($p<0.05$; higher anti-KLH (and other protein) Ab titers w sensitisation treatment ($p<0.05$); Penh response not sig dif to controls; KLH lowest IgG1:IgG2 ratio (more protective than allergenic); relative immunogenic potency: SUB>OVA≥KLH>BLG>MSA	0.5 - 25 µg	[43]
	Model investigating the influence of stress on the pulmonary immune response using Wistar rats (n=6/group) primed w 10 µg trinitrophenyl-KLH in saline i.p. subjected to mild electric foot shock stress challenged w 150 µg i.t. TNP-KLH; assays 10 d later	Higher anti-TNP Ab titers w stress treatment ($p<0.05$); acute emotional stress can contribute to the onset or severity of allergic asthma by lowering the threshold of induction of antigen-specific IgE production in the lungs	10 µg antigen-KLH	[44]
	Model of inherited pulmonary immunity post lung transplant using Beagle dogs (n=8 total); donors sensitised w 10 mg KLH instilled into left cardiac lung lobe (and control lobe), repeated KLH challenge; lungs transplanted 6 d after challenge; Abs monitored	Anti-KLH IgG, IgM, IgA Abs produced in recipient, gradually declining over 15 d; IgA and IgG continued to be produced by the donor for as long as 320 d; immune cells from asthmatic donor lungs could confer asthmatic responses in recipients	10 mg sens, 1mg challenge	[45]
	In vivo human study of 9 atopic asthma patients (+ 9 healthy subjects) immunised w 500 µg KLH injected into superior lingula division of lung (and control lobe) to characterise normal vs atypical immune function	Abs higher for asthmatic group, IgG4 sig ($p<0.05$), consistent w a Th2 response; serum/BALF Ab levels correlated ($p<0.05$); no sig dif in inflammatory cell counts; higher IgA1, IgM Abs in immunised lobe ($p<0.05$); >MCh reactivity in asthmatics; no adverse effects of KLH treatment	500 µg	[46]
	In vivo human study involving 51 healthy atopic subjects (likely to develop allergy) (n=4-8/group) sensitised w 0.1, 10, 1000 or 100 000 µg KLH (day 0) w successive 100 µg doses (day 14, 28) w or w/out adjuvant diesel exhaust particles (DEPs) (0.3mg) (day 1, 13 and 27) all delivered i.n. over 33-d study period	IgE decreased w increasing sensitisation dose ($p<0.003$); no specific IgE in 0.1 µg group; all developed detectable IgG and IgG4 Abs, sig higher for 100,000 µg group; IgE:IgG/IgG4 ratios decreased w increasing dose ($p=0.0004$); high dose overcame proallergic adjuvant effects of DEPs; initial high dose exposure to KLH is more likely to result in allergic tolerance rather than sensitisation	10-100,000 µg sens, 100 µg challenge	[47]
	In vivo human study involving 25 healthy atopic subjects challenged w 0.3mg DEPs i.n. 24 hr before aerosol KLH administration i.n. (1 mg or 100 µg in 200 µL saline), or KLH w/out prior DEP exposure, 3 x over 32-d study period	Abs elevated after 14-d and w each subsequent challenge; IgG and IgA similar in DEP+KLH and KLH alone, IgG4 and IgE higher in DEP+KLH ($p<0.01$); IgE did not occur w/out DEP pre-treatment; IL-4 increased w DEP+KLH, otherwise cytokines not sig different compared to pre-immunisation; DEP drives sensitisation to KLH in atopic subjects under conditions when KLH alone does not have this effect	1 mg or 100 µg	[48]
	In vivo human study involving healthy atopic and normal subjects (5 groups re normal, atopic or history of respiratory disease/allergy, n=10-14/group) treated w 300 µg aerosol KLH weekly administered via nebuliser over 2 mo + 600 µg booster or; monthly i.d. injections of 20 or 2 µg KLH and monthly i.c. injections of 22 µg KLH for skin tests	Skin reactivity higher in subjects w existing allergy/family history of respiratory disease (61% aerosol, 53% i.d.) compared to normal (25% aerosol, 30% i.d.) after 2 mo ($p<0.05$); developed more quickly in atopics (after 1 month); normal and atopic individuals develop skin hypersensitivity to KLH after repeated exposure, particularly to i.n. aerosol administration; no adverse symptoms	300 µg aerosol or 2-20 µg i.d.	[49]

Abbreviations: Ab: antibody; Hc: hemocyanin; w: with; KLH: keyhole limpet hemocyanin; PBS: phosphate buffer solution; OVA: ovalbumin; MCh: methacholine (bronchoconstrictor); BALF: bronchoalveolar lavage fluid; ELISA: enzyme-linked immunosorbent assay; DEPs: diesel exhaust particles; TNP: trinitrophenyl.

[1] Administration routes: s.c.- subcutaneous, i.p.- intraperitoneal, i.n.- intranasal, p.o.- per oral

[2] All hemocyanins derived from hemolymph; KLH laboratory grade, HpH experimentally purified

	In vivo human study of 10 cancer patients including those w bronchial and nasopharynx carcinoma (plus 14 normal subjects) immunised s.c. w 0.01, 0.1 or 5 mg KLH	KLH induced non-specific lymphocyte stimulation independent of dose in cancer patients and controls; Ab and lymphocyte response sig depressed in advanced disease (Group 2); KLH can assist in cancer treatment during which chemotherapy or the disease itself can impair the immune response	0.01 - 5 mg KLH*	[50]
	In vivo human study of 12 asthmatic children immunised s.c. w 320 µgN (~2 mg protein) KLH (0 and 14 d), 6 children receiving prednisolone (+ other steroid) treatment; also included 2 adult men immunised w 100 µgN KLH (0 d), 40 µgN (day 14), 350 µgN (44 d)	All subjects produced anti-KLH Ab titers; steroid treatment had no effect on antibody levels formed; no delayed hypersensitivity skin reactions or adverse reactions to mollusc food	2 mg KLH*	[51]
	Asthma model using Balb/c mice (n=7-10/group) immunised s.c. w 100 µg KLH and separately w fused/non-fused Fc proteins (5 mg/kg) (or proteins alone) challenged w 0.5% aerosol OVA and treated w Fc proteins i.p. (5 mg/kg) (KLH not used for challenge)	KLH with protein immunisation elicited a stronger anti-KLH immune response than protein alone; strongest effects on anti-KLH IgM, IgG and IgG1 Abs, lymphocytes, IL-10 and IFN-γ; B7RP-1-Fc identified as stimulant (>cytokine, inflammatory cell and anti-KLH Ab expression); CTLA-4 identified as suppressant (<anti-KLH Abs)	100 µg KLH	[52]
Helicidae	*In vivo* human study of 11 asthmatic patients (and 10 normal subjects) immunised s.c. w 1.0 mg HpH, monitored over 32 d	IgE and IgG Ab titers sig higher in asthmatics (p<0.01); IgM higher at 7 d (p<0.05) and IgA generally higher though not sig; increased humoral responsiveness of asthmatic patients is not restricted to the IgE class	1.0 mg HpH*	[53]
	In vivo human study of 30 patients w squamous cell bronchial carcinoma and 15 w chronic bronchitis (smokers w no carcinoma as controls) immunised w 1 mg HpH s.c. at time of diagnosis	Advanced disease stages showed sig lower IgA and IgG Ab responses (=<0.001), IgM not different from controls; Stage I and III disease lower lymphocyte transformation (p<0.01); SQBC causes impaired humoral and cellular immune responses and a defective Ab response	1mg HpH*	[54]
	In vivo human study of 11 asthma patients (plus 9 healthy subjects) immunised s.c. w 1 mg HpH	Blood hydrocortisone higher in asthmatics (not sig); hydrocortisone had an inhibitory effect on HpH-induced lymphocyte proliferation; endogenous hydrocortisone plays an important immunosuppressive role in asthma	1mg HpH*	[55]

Table S5: Selected patents relating to pharmaceutical compounds derived from molluscs for the treatment of respiratory disease.

Class<br>Family	Derivative part	Specific extract/compound	Bioactivity	Related respiratory disease	Patent title	Country, patent ID
**Bivalvia**						
Mactridae	NA	Spisulosine and related compounds	Anticancer	Lung cancer; ORI	"Spisulosine compounds."	United States. US6800661B1 (2004)
Mytilidae	Body	Lipid extract	Anti-inflammatory	Asthma; pulmonary inflammation and allergy	a) "A product containing anti-inflammatory principles from green mussel *Perna viridis* L. and a process thereof."<br>b) "Lipid extract of mussels and method for preparation thereof"<br>c) "An extract"	a) India. IP2066/CHE/2010 (2013)<br>b) International. WO2006128244A1 (2006)<br>c) International. WO2008075978A2. (2008)
	Hemolymph	Mytilin	Antiviral	Viral infection	"Mytilin as well as preparation method and application thereof in preparation of medicines and feed additives"	China. CN104418946A (2015)
**Gastropoda**						
Aplysiidae	Body	Dolastatin 10, 15 and related compounds (e.g. Tasidotin, Elisidepsin, Soblidotin)	Anticancer	Lung cancer	a) "Derivatives of Dolastatin 10 and auristatins."<br>b) "Three-dimensional structure of new antitumor agent Soblidotin."	a) United States. US20160083420A1 (2016)<br>b) Japan. JP2004262799A (2004)
		Vedotin compounds	Anticancer	Lung cancer	a) "Carrier-binding agent compositions and methods of making and using the same."	a) Australia. AU2016308337A1. (2019)
Fissurellidae	Hemolymph	Hemocyanin	Immunomodulatory (cancer vaccine conjugate), antimicrobial	ORI; lung cancer	a) "Adjuvant enhanced immunotherapy."<br>b) "Composition for delivering bioactive agents for immune response and its preparation."<br>c) "Preparation methods for a novel generation of biological safe KLH products used for cancer treatment, for the development of conjugated therapeutic vaccines and as challenging agents."<br>d) "Purified hemocyanin obtained from *Fissurella latimarginata, F. cumingi* or *F. maxima*; subunit of purified hemocyanin; use of hemocyanin, its subunit or immunogenic fragments and compositions containing the same."	a) Australia. AU2003224989B2 (2008)<br>b) United States. US6024983A (2000)<br>c) International. WO/2015/188868 (2019)<br>d) United States. US20100255017A1 (2009)
Haliotidae	Hemolymph	Hemocyanin or crude hemolymph	Antiviral	Viral infection (including rhinovirus, influenza, common cold)	"Anti-viral nutraceutical."	International. WO2009129561A1 (2009)
Muricidae	Hemolymph	Hemocyanin (specific subunits or extract)	Immunomodulatory (cancer vaccine conjugate), antimicrobial	ORI; lung cancer	a) "Product and composition containing a *Concholepas concholepas* hemocyanin (cch) subunit a, and a method of use thereof."<br>b) "Hemocyte extract loco (*Concholepas concholepas*) with antimicrobial activity, pharmaceutical composition comprising said extract; method for producing the extract; use of the extract that serves food additive or aids to retain and prevent contamination with microorganisms."	a) International. WO2005014647A1 (2005)<br>b) Chile. CL2013000660A1. (2013)
	Hypobranchial gland (also in egg masses)	Indirubin and indole derivatives (e.g. indirubin-3'-oxime; 3',6-substituted indirubins [6-BIO]; 6-(3-phenylpropyl) amino-2-benzoxazolone; 'Natura'; E804)	Anticancer, antimicrobial, anti-inflammatory	ORI; lung cancer; pulmonary inflammation and allergy	a) "Indole derivatives as antiallergy and anti-inflammatory agents."<br>b) "Derivatives of isoindigo, indigo and indirubin and methods of treating cancer."<br>c) "3',6-substituted indirubins and their biological applications."<br>d) "Methods and pharmaceutical compositions for inhibiting influenza viruses replication."	a) United States. 5290788 (1994)<br>b) United States. US6933315B2 (2005)<br>c) United States. US8829203B2 (2014)<br>d) United States. US9168236B2. (2015)
Muricidae, Helicidae	Hemolymph	Hemocyanin (specific subunits or extract)	Antimicrobial	ORI	a) "A biologically active commodity."<br>b) "Bio components from snails."	a) Bulgaria. BG110495A (2011)<br>b) Bulgaria. BG110665A (2011)
Plakobranchidae	Body	Kahaladide F (and related compounds)	Anticancer and antiviral	Lung cancer (and viral infections)	"Cytotoxic and antiviral compound."	Europe. EP0610078A1 (1994)

Abbreviations: ORI: opportunistic respiratory infection

Table S6: Classes of purified compounds tested against respiratory pathogens, cancers and inflammatory diseases.

Purified compound	Compound class	Bioactivity	Reference
3 protamine-like proteins	Protein	Antibacterial	[56]
Myticin, Myticin B, Myticin C and 9 peptides	Peptide	Antibacterial, antifungal	[57], [58]
Tartrolon E	Polyketide	Antibacterial	[59]
OctoPartenopin + 6 HPLC fractions + 5 synthesised fractions	Peptide	Antibacterial	[60]
Mytimicin-AF	Peptide	Antibacterial	[61]
Conotoxin MVIIA and 9 analogues	Peptide	Antibacterial	[62]
5'-deoxy-5'-methylthio-adenosine (MTA) + two naturally-occurring analogues (xylo-MTA, xylo-A)	Protein	Antibacterial	[63]
Scutinin A and B	Polyketide	Antibacterial	[64]
Purified Hc from *Helix aspera* (βc-HaH subunit + 8 functional units)	Glycoprotein, peptide	Antibacterial	[65]
Purified Hc from *Rapana venosa* (RvH), glycosylated (RvH-c) + non-glycosylated (RvH-b) subunits + 4 functional units and 11 protein fractions	Glycoprotein, protein, peptide	Antiviral	[66], [67], [68]
Purified Hc from *Helix pomatia* (HpH)	Glycoprotein	Anticancer, immunomodulatory	[68]
Tyriverdin, tyrindoleninone, Tyrian purple, 6-bromoisatin	Brominated indole/isatin derivatives	Antibacterial, anticancer, anti-inflammatory	[69], [70]
Dolabellanin B2	Peptide	Antibacterial	[71],
2 sialic acid-binding lectin recombinant proteins (rSgSABL-1, -2)	Protein	Antibacterial, immunomodulatory	[72]
Kahalalide F and derivatives (e.g. Elisidepsin/PM02734, KZ1, KZ2)	Peptide	Antibacterial, antifungal, anticancer	[73], [74], [75], [76], [77], [78], [79, 80], [81]
7,8-dideoxygriseorhodin C	Aromatic polyketide	Antibacterial	[82]
Four mucus fractions (PUFA 39, 40, 49, 50)	Fatty acid derivative	Antiviral	[83]
Three mucus fractions (PUFA 39, 40, 49)	Fatty acid derivative	Antiviral	[84]
Spisulosine	Amino acid	Anticancer	[85]
Tumor necrosis factor (*Cg*TNF-2)	Protein	Anticancer	[86]
$(NH_4)_2SO_4$ fractionated peptide ('Mere15')	Peptide	Anticancer	[87]
Experimentally purified Hc (HpH)	Glycoprotein	Anticancer	[38]
'Lyprinol'	Lipid formulation	Anti-inflammatory	[88], [89], [90], [91]
Heparin sulfate analog (HS)	Polysaccharide	Anticancer	[92]
Dolastatin-10 and derivatives (e.g. Solibidotin/TZT-1027)	Peptide	Anticancer	[93], [94]

Abbreviations: Hc: hemocyanin; PUFA: polyunsaturated fatty acid.

References

1. Alves, R. R.; Alves, H. N., The faunal drugstore: animal-based remedies used in traditional medicines in Latin America. J Ethnobiol Ethnomed 2011, 7, (1), 9.
2. Kim, H.; Song, M. J., Ethnozoological study of medicinal animals on Jeju Island, Korea. J Ethnopharmacol 2013, 146, (1), 75-82.
3. Brieger, J. E., Calcarea carbonica or Ostrea edulis? Br Homeopath J 1960, 49, (01), 41-43.
4. Ramchandani, N. M., Homoeopathic treatment of upper respiratory tract infections in children: evaluation of thirty case series. Complement Ther Clin Pract 2010, 16, (2), 101-108.
5. Colin, P., Homeopathy and respiratory allergies: a series of 147 cases. Homeopathy 2006, 95, (2), 68-72.
6. Gopal, R.; Vijayakumaran, M.; Venkatesan, R.; Kathiroli, S., Marine organisms in Indian medicine and their future prospects. Indian J Nat Prod Resour 2008, 7, (2), 139-145.
7. Alves, R. R.; Rosa, I. L., Zootherapeutic practices among fishing communities in North and Northeast Brazil: a comparison. J Ethnopharmacol 2007, 111, (1), 82-103.
8. Meyer-Rochow, V. B., Therapeutic arthropods and other, largely terrestrial, folk-medicinally important invertebrates: a comparative survey and review. J Ethnobiol Ethnomed 2017, 13, (1).
9. Voultsiadou Eleni, E., Therapeutic properties and uses of marine invertebrates in the ancient Greek world and early Byzantium. J Ethnopharmacol 2010, 130, (2), 237-247.
10. Alade, G. O.; Frank, A.; Ajibesin, K. K., Animals and animal products as medicines: a survey of Epie-Atissa and Ogbia people of Bayelsa State, Nigeria. J Pharm Pharmacogn Res 2018, 6, (6), 483-502.
11. Jamir, N.; Lal, P., Ethnozoological practices among Naga tribes. Indian J Trad Knowl 2005, 4, (1), 100-104.
12. Meyerhof, M.; Sobhy, G. P., The abridged version of "The Book of Simple Drugs", of Ahmad Ibn Muhammad Al-Ghafiqi by Gregorius Abul-Farag. Al Ettemad Printing Press and Publising House: Cairo, Egypt, 1932; p 224–228.
13. Liang, X.; Wang, J.; Guan, R.; Zhao, L.; Li, D.; Long, Z.; Yang, Q.; Xu, J.; Wang, Z.; Xie, J.; Lu, W., Limax extract ameliorates cigarette smoke-induced chronic obstructive pulmonary disease in mice. Int Immunopharmacol 2018, 54, 210-220.
14. Bonnemain, B., Helix and drugs: snails for western health care from antiquity to the present. Evid Based Complement Alternat Med 2005, 2, (1), 25-28.
15. Chinlampianga, M.; Singh, R. K.; Shukla, A. C., Ethnozoological diversity of Northeast India: empirical learning with traditional knowledge holders of Mizoram and Arunachal Pradesh. Indian J Trad Knowl 2013, 12, (1), 18-30.
16. Nongmaithem, B. D.; Mouatt, P.; Smith, J.; Rudd, D.; Russell, M.; Sullivan, C.; Benkendorff, K., Volatile and bioactive compounds in opercula from Muricidae molluscs supports their use in ceremonial incense and traditional medicines. Sci Rep 2017, 7, (1), 1-14.
17. McHugh, J., Blattes de byzance in India: mollusk opercula and the history of perfumery. J R Asiat Soc 2013, 23, (1), 53-67.
18. Lev, E.; Zohar, A., Practical materia medica of the Medieval Eastern Mediterranean according to the Cairo Genizah (Vol 7). Brill Online Books and Journals: Leiden, The Netherlands, 2008.
19. Sun, B. N.; Shen, H. D.; Wu, H. X.; Yao, L. X.; Cheng, Z. Q.; Diao, Y., Determination of chemical constituents of the marine pulmonate slug, Paraoncidium reevesii. Tropic J Pharm Res 2014, 13, (12), 2071-2074.
20. Alves, R. R.; Rosa, I. L., Zootherapy goes to town: the use of animal-based remedies in urban areas of NE and N Brazil. J Ethnopharmacol 2007, 113, (3), 541-555.
21. Prabhakar, A. K.; Roy, S. P., Ethno-medicinal uses of some shell fishes by people of Kosi river basin of North-Bihar, India. Studies on Ethno-Medicine 2009, 3, (1), 1-4.
22. Guan, H.; Wang, S., Chinese Marine Materia Medica. Shanghai Scientific and Technical Publishers, China Ocean Press, and Chemical Industry Press: Shanghai, Beijing, China, 2009; p 371.
23. Dickler, M. N.; Ragupathi, G.; Liu, N. X.; Musselli, C.; Martino, D. J.; Miller, V. A.; Kris, M. G.; Brezicka, F. T.; Livingston, P. O.; Grant, S. C., Immunogenicity of a fucosyl-GM1-keyhole limpet hemocyanin conjugate vaccine in patients with small cell lung cancer. Clin Cancer Res 1999, 5, (10), 2773-2779.
24. Demoly, P.; Persi, L.; Dhivert, H.; Delire, M.; Bousquet, J., Immunotherapy with keyhole limpet hemocyanin-conjugated decapeptide vaccine in cypress pollen allergy. Clin Exp Allergy 2002, 32, (7), 1071-1076.
25. Miles, D.; Roché, H.; Martin, M.; Perren, T. J.; Cameron, D. A.; Glaspy, J.; Dodwell, D.; Parker, J.; Mayordomo, J.; Tres, A.; Murray, J. L.; Ibrahim, N. K., Phase III multicenter clinical trial of the sialyl-TN (STn)-keyhole limpet hemocyanin (KLH) vaccine for metastatic breast cancer. Oncologist 2011, 16, (8), 1092-1100.
26. Hasumi, K.; Aoki, Y.; Watanabe, R.; Hankey, K. G.; Mann, D. L., Therapeutic response in patients with advanced malignancies treated with combined dendritic cell-activated T cell based immunotherapy and intensity-modulated radiotherapy. Cancers (Basel) 2011, 3, (2), 2223-2242.
27. Hou, Y.; Gu, X.-X., Development of peptide mimotopes of lipooligosaccharide from nontypeable Haemophilus influenzae as vaccine candidates. J Immunol 2003, 170, (8), 4373-4379.

28. Hughes, E. E.; Gilleland, L. B.; Gilleland Jr, H. E., Synthetic peptides representing epitopes of outer membrane protein F of Pseudomonas aeruginosa that elicit antibodies reactive with whole cells of heterologous immunotype strains of P. aeruginosa. Infect Immun 1992, 60, (9), 3497-3503.
29. Hughes, E. E.; Gilleland Jr, H. E., Ability of synthetic peptides representing epitopes of outer membrane protein F of Pseudomonas aeruginosa to afford protection against P. aeruginosa infection in a murine acute pneumonia model. Vaccine 1995, 13, (18), 1750-1753.
30. Tan, H. X.; Jegaskanda, S.; Juno, J. A.; Esterbauer, R.; Wong, J.; Kelly, H. G.; Liu, Y.; Tilmanis, D.; Hurt, A. C.; Yewdell, J. W.; Kent, S. J.; Wheatley, A. K., Subdominance and poor intrinsic immunogenicity limit humoral immunity targeting influenza HA stem. J Clin Invest 2019, 129, (2), 850-862.
31. Wallmann, J.; Epstein, M. M.; Singh, P.; Brunner, R.; Szalai, K.; El-Housseiny, L.; Pali-Schöll, I.; Jensen-Jarolim, E., Mimotope vaccination for therapy of allergic asthma: anti-inflammatory effects in a mouse model. Clin Exp Allergy 2010, 40, (4), 650-658.
32. Sen-Kilic, E.; Blackwood, C. B.; Boehm, D. T.; Witt, W. T.; Malkowski, A. C.; Bevere, J. R.; Wong, T. Y.; Hall, J. M.; Bradford, S. D.; Varney, M. E.; Damron, F. H.; Barbier, M., Intranasal peptide-based fpva-klh conjugate vaccine protects mice from Pseudomonas aeruginosa acute murine pneumonia. Front Immunol 2019, 10, (OCT).
33. Shin, H. J.; Franco, L. H.; Nair, V. R.; Collins, A. C.; Shiloh, M. U., A baculovirus-conjugated mimotope vaccine targeting Mycobacterium tuberculosis lipoarabinomannan. PLoS One 2017, 12, (10).
34. Tebbey, P. W.; Hagen, M.; Hancock, G. E., Atypical pulmonary eosinophilia is mediated by a specific amino acid sequence of the attachment (G) protein of respiratory syncytial virus. J Exp Med 1998, 188, (10), 1967-1972.
35. Theilacker, C.; Coleman, F. T.; Mueschenborn, S.; Llosa, N.; Grout, M.; Pier, G. B., Construction and characterization of a Pseudomonas aeruginosa mucoid exopolysaccharide-alginate conjugate vaccine. Infect Immun 2003, 71, (7), 3875-3884.
36. Sokol, P. A.; Kooi, C.; Hodges, R. S.; Cachia, P.; Woods, D. E., Immunization with a Pseudomonas aeruginosa elastase peptide reduces severity of experimental lung infections due to P. aeruginosa or Burkholderia cepacia. J Infect Dis 2000, 181, (5), 1682-1692.
37. Kyd, J. M.; Cripps, A. W.; Novotny, L. A.; Bakaletz, L. O., Efficacy of the 26-kilodalton outer membrane protein and two P5 fimbrin-derived immunogens to induce clearance of nontypeable Haemophilus influenzae from the rat middle ear and lungs as well as from the chinchilla middle ear and nasopharynx. Infect Immun 2003, 71, (8), 4691-4699.
38. Gesheva, V.; Chausheva, S.; Stefanova, N.; Mihaylova, N.; Doumanova, L.; Idakieva, K.; Tchorbanov, A., Helix pomatia hemocyanin - a novel bio-adjuvant for viral and bacterial antigens. Int Immunopharmacol 2015, 26, (1), 162-168.
39. Gesheva, V.; Idakieva, K.; Kerekov, N.; Nikolova, K.; Mihaylova, N.; Doumanova, L.; Tchorbanov, A., Marine gastropod hemocyanins as adjuvants of non-conjugated bacterial and viral proteins. Fish Shellfish Immunol 2011, 30, (1), 135-142.
40. Hochscheid, R.; Schreiber, N.; Kotte, E.; Weber, P.; Cassel, W.; Yang, H.; Zhang, Y.; Pöschl, U.; Müller, B., Nitration of protein without allergenic potential triggers modulation of antioxidant response in type II pneumocytes. J Toxicol Environ Health - Part A: Current Issues 2014, 77, (12), 679-695.
41. Thepen, T.; Van Rooijen, N.; Kraal, G., Alveolar macrophage elimination in vivo is associated with an increase in pulmonary immune response in mice. J Exp Med 1989, 170, (2), 499-509.
42. Kobayashi, T.; Iijima, K.; Checkel, J. L.; Kita, H., IL-1 family cytokines drive Th2 and Th17 cells to innocuous airborne antigens. Am J Respir Cell Mol Biol 2013, 49, (6), 989-998.
43. Krieger, S. M.; Boverhof, D. R.; Woolhiser, M. R.; Hotchkiss, J. A., Assessment of the respiratory sensitization potential of proteins using an enhanced mouse intranasal test (MINT). Food Chem Toxicol 2013, 59, 165-176.
44. Persoons, J. H. A.; Berkenbosch, F.; Schornagelb, K.; Thepen, T.; Kraal, G., Increased specific IgE production in lungs after the induction of acute stress in rats. J Allergy Clin Immunol 1995, 95, (3), 765-770.
45. Bice, D. E.; Williams, A. J.; Muggenburg, B. A., Long-term Antibody Production in Canine Lung Allografts: Implications in Pulmonary Immunity and Asthma. Am J Respir Cell Mol Biol 1996, 14, (4), 341-347.
46. Schuyler, M.; Lyons, C. R.; Masten, B.; Bice, D., Immunoglobulin response to intrapulmonary immunization of asthmatics. Immunol 1997, 91, (2), 167-175.
47. Riedl, M. A.; Landaw, E. M.; Saxon, A.; Diaz-Sanchez, D., Initial high-dose nasal allergen exposure prevents allergic sensitization to a neoantigen. J Immunol 2005, 174, (11), 7440-7445.
48. Diaz-Sanchez, D.; Garcia, M. P.; Wang, M.; Jyrala, M.; Saxon, A., Nasal challenge with diesel exhaust particles can induce sensitization to a neoallergen in the human mucosa. J Allergy Clin Immunol 1999, 104, (6), 1183-1188.
49. Salvaggio, J.; Castro-Murillo, E.; Kundur, V., Immunologic response of atopic and normal individuals to keyhole limpet hemocyanin. J Allergy 1969, 44, (6), 344-354.
50. Curtis, J. E.; Hersh, E. M.; Harris, J. E.; McBride, C.; Freireich, E. J., The human primary immune response to keyhole limpet haemocyanin: interrelationships of delayed hypersensitivity, antibody response and in vitro blast transformation. Clin Exp Immunol 1970, 6, (4), 473-491.

51. Tuchinda, M.; Newcomb, R. W.; DeVald, B. L., Effect of prednisone treatment on the human immune response to keyhole limpet hemocyanin. Int Arch Allergy Immunol 1972, 42, (4), 533-544.
52. Guo, J.; Stolina, M.; Bready, J. V.; Yin, S.; Horan, T.; Yoshinaga, S. K.; Senaldi, G., Stimulatory effects of B7-related protein-1 on cellular and humoral immune responses in mice. J Immunol 2001, 166, (9), 5578-5584.
53. Weller, F. R.; Kallenberg, C. G. M.; Jansen, H. M.; Torensma, R.; Klaassen, R. J. L.; Weller, H. H.; Orie, N. G. M.; The, T. H., The primary immune response in bronchial asthma. I. A kinetic study of elix pomatia hemocyanin-specific IgE, IgG, IgA, and IgM antibody responses in patients with asthma and in matched controls. J Allergy Clin Immunol 1985, 76, (1), 29-34.
54. Jansen, H. M.; The, T. H.; de Gast, G. C.; Esselink, M. T.; Pastoor, G.; Orie, N. G., The primary immune response of patients with different stages of squamous-cell bronchial carcinoma. Thorax 1978, 33, (6), 755-760.
55. Weller, F. R.; Weller, H. H.; Kallenberg, C. G. M.; The, T. H.; Orie, N. G. M., Sensitivity to hydrocortisone is a relevant factor in the immunoendocrine relationship. I. The cell-mediated immune response in relation to blood levels and in vitro immunosuppressive effects of hydrocortisone in patients with asthma and healthy control subjects. J Allergy Clin Immunol 1986, 78, (3 PART 1), 423-430.
56. Notariale, R.; Basile, A.; Montana, E.; Romano, N. C.; Cacciapuoti, M. G.; Aliberti, F.; Gesuele, R.; De Ruberto, F.; Sorbo, S.; Tenore, G. C.; Guida, M.; Good, K. V.; Ausio, J.; Piscopo, M., Protamine-like proteins have bactericidal activity: the first evidence in Mytilus galloprovincialis. Acta Biochim Pol 2018, 65, (4), 585-594.
57. Domeneghetti, S.; Franzoi, M.; Damiano, N.; Norante, R.; M. El Halfawy, N.; Mammi, S.; Marin, O.; Bellanda, M.; Venier, P., Structural and antimicrobial features of peptides related to Myticin C, a special defense molecule from the Mediterranean mussel Mytilus galloprovincialis. J Agric Food Chem 2015, 63, (42), 9251-9259.
58. Mitta, G.; Hubert, F.; Noel, T.; Roch, P., Myticin, a novel cysteine-rich antimicrobial peptide isolated from haemocytes and plasma of the mussel Mytilus galloprovincialis. Eur J Biochem 1999, 265, (1), 71-78.
59. Elshahawi, S. I.; Trindade-Silva, A. E.; Hanora, A.; Han, A. W.; Flores, M. S.; Vizzoni, V.; Schrago, C. G.; Soares, C. A.; Concepcion, G. P.; Distel, D. L.; Schmidt, E. W.; Haygood, M. G., Boronated tartrolon antibiotic produced by symbiotic cellulose-degrading bacteria in shipworm gills. Proc Natl Acad Sci U S A 2013, 110, (4), E295-E304.
60. Maselli, V.; Galdiero, E.; Salzano, A. M.; Scaloni, A.; Maione, A.; Falanga, A.; Naviglio, D.; Guida, M.; Di Cosmo, A.; Galdiero, S., OctoPartenopin: Identification and preliminary characterization of a novel antimicrobial peptide from the suckers of Octopus vulgaris. Mar Drugs 2020, 18, (8).
61. Zhong, J.; Wang, W.; Yang, X.; Yan, X.; Liu, R., A novel cysteine-rich antimicrobial peptide from the mucus of the snail of Achatina fulica. Peptides 2013, 39, (1), 1-5.
62. Hemu, X.; Tam, J. P., Macrocyclic antimicrobial peptides engineered from ω-conotoxin. Curr Pharm Des 2017, 23, (14), 2131-2138.
63. Pani, A.; Marongiu, M. E.; Obino, P.; Gavagnin, M.; La Colla, P., Antimicrobial and antiviral activity of xylosyl-methylthio-adenosine, a naturally occurring analogue of methylthio-adenosine from Doris verrucosa. Experientia 1991, 47, (11-12), 1228-1229.
64. Chand, S.; Karuso, P., Isolation and total synthesis of two novel metabolites from the fissurellid mollusc Scutus antipodes. Tetrahedron Lett 2017, 58, (10), 1020-1023.
65. Dolashka, P.; Dolashki, A.; Van Beeumen, J.; Floetenmeyer, M.; Velkova, L.; Stevanovic, S.; Voelter, W., Antimicrobial activity of molluscan hemocyanins from Helix and Rapana snails. Curr Pharm Biotechnol 2016, 17, (3), 263-270.
66. Dolashka-Angelova, P.; Lieb, B.; Velkova, L.; Heilen, N.; Sandra, K.; Nikolaeva-Glomb, L.; Dolashki, A.; Galabov, A. S.; Van Beeumen, J.; Stevanovic, S.; Voelter, W.; Devreese, B., Identification of glycosylated sites in Rapana hemocyanin by mass spectrometry and gene sequence, and their antiviral effect. Bioconj Chem 2009, 20, (7), 1315-1322.
67. Dolashka, P.; Moshtanska, V.; Borisova, V.; Dolashki, A.; Stevanovic, S.; Dimanov, T.; Voelter, W., Antimicrobial proline-rich peptides from the hemolymph of marine snail Rapana venosa. Peptides 2011, 32, (7), 1477-1483.
68. Gesheva, V.; Chausheva, S.; Mihaylova, N.; Manoylov, I.; Doumanova, L.; Idakieva, K.; Tchorbanov, A., Anti-cancer properties of gastropodan hemocyanins in murine model of colon carcinoma. BMC Immunol 2014, 15, (1).
69. Benkendorff, K.; Bremner, J. B.; Davis, A. R., Tyrian purple precursors in the egg masses of the Australian muricid, Dicathais orbita: a possible defensive role. J Chem Ecol 2000, 26, (4), 1037-1050.
70. Ahmad, T. B.; Rudd, D.; Benkendorff, K.; Mahdi, L. K.; Pratt, K. A.; Dooley, L.; Wei, C.; Kotiw, M., Brominated indoles from a marine mollusc inhibit inflammation in a murine model of acute lung injury. PLoS One 2017, 12, (10).
71. Bitaab, M. A.; Ranaei Siadat, S. O.; Pazooki, J.; Sefidbakht, Y., Antibacterial and molecular dynamics study of the Dolabellanin B2 isolated from sea slug, Peronia peronii. Biosci Biotechnol Res Asia 2015, 12, (3), 2023-2035.
72. Wei, X. M.; Yang, D. L.; Li, H. Y.; Jiang, H. L.; Liu, X. Q.; Zhang, Q.; Yang, J. L., Sialic acid-binding lectins (SABLs) from Solen grandis function as PRRs ensuring immune recognition and bacterial clearance. Fish Shellfish Immunol 2018, 72, 477-483.

73. Shilabin, A. G.; Hamann, M. T., In vitro and in vivo evaluation of select kahalalide F analogs with antitumor and antifungal activities. Bioorg Med Chem 2007, 19, (22), 6628-6632.
74. Janmaat, M. L.; Rodriguez, J. A.; Jimeno, J.; Kruyt, F. A. E.; Giaccone, G., Kahalalide F induces necrosis-like cell death that involves depletion of ErbB3 and inhibition of Akt signaling. Mol Pharmacol 2005, 68, (2), 502-510.
75. Teixidó, C.; Arguelaguet, E.; Pons, B.; Aracil, M.; Jimeno, J.; Somoza, R.; Marés, R.; Ramón Y Cajal, S.; Hernández-Losa, J., ErbB3 expression predicts sensitivity to elisidepsin treatment: in vitro synergism with cisplatin, paclitaxel and gemcitabine in lung, breast and colon cancer cell lines. Int J Oncol 2012, 41, (1), 317-324.
76. Ling, Y. H.; Aracil, M.; Jimeno, J.; Perez-Soler, R.; Zou, Y., Molecular pharmacodynamics of PM02734 (elisidepsin) as single agent and in combination with erlotinib; synergistic activity in human non-small cell lung cancer cell lines and xenograft models. Eur J Cancer 2009, 45, (10), 1855-1864.
77. Ciavatta, M. L.; Devi, P.; Carbone, M.; Mathieu, V.; Kiss, R.; Casapullo, A.; Gavagnin, M., Kahalalide F analogues from the mucous secretion of Indian sacoglossan mollusc Elysia ornata. Tetrahedron 2016, 72, (5), 625-631.
78. Pardo, B.; Paz-Ares, L.; Tabernero, J.; Ciruelos, E.; Garcia, M.; Salazar, R.; Lopez, A.; Blanco, M.; Nieto, A.; Jimeno, J.; Izquierdo, M. A.; Trigo, J. M., Phase I clinical and pharmacokinetic study of kahalalide F administered weekly as a 1-hour infusion to patients with advanced solid tumors. Clin Cancer Res 2008, 14, (4), 1116-1123.
79. Hamann, M. T., Technology evaluation: Kahalalide F, PharmaMar. Curr Opin Mol Ther 2004, 6, (6), 657-665.
80. PharmaMar, PharmaMar reports new data on Kahalalide-F and Aplidin(R) at ESMO congress. Presented at the 31st European Society for Medical Oncology Congress (ESMO); Istanbul, Turkey. September 29 - October 3, 2006., 2006.
81. Salazar, R.; Jones, R. J.; Oaknin, A.; Crawford, D.; Cuadra, C.; Hopkins, C.; Gil, M.; Coronado, C.; Soto-Matos, A.; Cullell-Young, M.; Iglesias Dios, J. L.; Evans, T. R. J., A phase I and pharmacokinetic study of elisidepsin (PM02734) in patients with advanced solid tumors. Cancer Chemother Pharmacol 2012, 70, (5), 673-681.
82. Miller, B. W.; Torres, J. P.; Tun, J. O.; Flores, M. S.; Forteza, I.; Rosenberg, G.; Haygood, M. G.; Schmidt, E. W.; Concepcion, G. P., Synergistic anti-methicillin-resistant Staphylococcus aureus (MRSA) activity and absolute stereochemistry of 7,8-dideoxygriseorhodin C. J Antibiot 2020, 73, 290-298.
83. de Toledo-Piza, A. R.; Figueiredo, C. A.; de Oliveira, M. I.; Negri, G.; Namiyama, G.; Tonelotto, M.; Villar, K. D.; Rofatto, H. K.; Mendonca, R. Z., The antiviral effect of mollusk mucus on measles virus. Antiviral Res 2016, 134, 172-181.
84. de Toledo-Piza, A. R.; de Oliveira, M. I.; Negri, G.; Mendonca, R. Z.; Figueiredo, C. A., Polyunsaturated fatty acids from Phyllocaulis boraceiensis mucus block the replication of influenza virus. Arch Microbiol 2018, 200, (6), 961-970.
85. Silveira-Dorta, G.; Sousa, I. J.; Fernandes, M. X.; Martín, V. S.; Padrón, J. M., Synthesis and identification of unprecedented selective inhibitors of CK1ε. Eur J Med Chem 2015, 96, 308-317.
86. Zheng, Y.; Liu, Z.; Wang, L.; Li, M.; Zhang, Y.; Zong, Y.; Li, Y.; Song, L., A novel tumor necrosis factor in the Pacific oyster Crassostrea gigas mediates the antibacterial response by triggering the synthesis of lysozyme and nitric oxide. Fish Shellfish Immunol 2020, 98, 334-341.
87. Wang, C.; Liu, M.; Cheng, L.; Wei, J.; Wu, N.; Zheng, L.; Lin, X., A novel polypeptide from Meretrix meretrix Linnaeus inhibits the growth of human lung adenocarcinoma. Exp Biol Med 2012, 237, (4), 442-450.
88. Wood, L. G.; Hazlewood, L. C.; Foster, P. S.; Hansbro, P. M., Lyprinol reduces inflammation and improves lung function in a mouse model of allergic airways disease. Clin Exp Allergy 2010, 40, (12), 1785-1793.
89. Emelyanov, A.; Fedoseev, G.; Krasnoschekova, O.; Abulimity, A.; Trendeleva, T.; Barnes, P. J., Treatment of asthma with lipid extract of New Zealand green-lipped mussel: a randomised clinical trial. Eur Respir J 2002, 20, (3), 596-600.
90. Lello, J.; Liang, A.; Robinson, E.; Leutenegger, D.; Wheat, A., Treatment of children's asthma with a lipid extract of the new Zealand green lipped mussel (Perna canaliculus) (Lyprinol®)—A double blind, randomized controlled trial in children with moderate to serve chronic obstructive asthma. Internet J Asthma Allergy Immunol 2012, 8.
91. Mickleborough, T. D.; Vaughn, C. L.; Shei, R. J.; Davis, E. M.; Wilhite, D. P., Marine lipid fraction PCSO-524™ (Lyprinol®/Omega XL®) of the New Zealand green lipped mussel attenuates hyperpnea-induced bronchoconstriction in asthma. Respir Med 2013, 107, (8), 1152-1163.
92. Gomes, A. M.; Kozlowski, E. O.; Borsig, L.; Teixeira, F.; Vlodavsky, I.; Pavao, M. S. G., Antitumor properties of a new non-anticoagulant heparin analog from the mollusk Nodipecten nodosus: effect on P-selectin, heparanase, metastasis and cellular recruitment. Glycobiology 2015, 25, (4), 386-393.
93. Kobayashi, M.; Natsume, T.; Tamaoki, S.; Watanabe, J.; Asano, H.; Mikami, T.; Miyasaka, K.; Miyazaki, K.; Gondo, M.; Sakakibara, K.; Tsukagoshi, S., Antitumor activity of TZT-1027, a novel dolastatin 10 derivative. Jap J Cancer Res 1997, 88, (3), 316-327.
94. Kalemkerian, G. P.; Ou, X.; Adil, M. R.; Rosati, R.; Khoulani, M. M.; Madan, S. K.; Pettit, G. R., Activity of dolastatin 10 against small-cell lung cancer in vitro and in vivo: induction of apoptosis and bcl-2 modification. Cancer Chemother Pharmacol 1999, 43, (6), 507-15.
